# No excessive mutations in transcription activator-like effector nuclease-mediated α-1,3-galactosyltransferase knockout Yucatan miniature pigs

**DOI:** 10.5713/ajas.19.0480

**Published:** 2019-08-23

**Authors:** Kimyung Choi, Joohyun Shim, Nayoung Ko, Joonghoon Park

**Affiliations:** 1 Optipharm Inc., Cheongju 28158, Korea; 2 Department of Animal Science and Biotechnology, Chungnam National University, Daejeon 34134, Korea; 3 Department of International Agricultural Technology, Graduate School of International Agricultural Technology, Seoul National University, Pyeongchang 25354, Korea; 4 Institute of Green Bio Science and Technology, Seoul National University, Pyeongchang 25354, Korea

**Keywords:** Transcription Activator-like Effector Nuclease (TALEN), Off-target, α-1, 3-Galactosyltransferase (GGTA1), Yucatan Miniature Pig, Whole-genome Sequencing, RNA Sequencing

## Abstract

**Objective:**

Specific genomic sites can be recognized and permanently modified by genome editing. The discovery of endonucleases has advanced genome editing in pigs, attenuating xenograft rejection and cross-species disease transmission. However, off-target mutagenesis caused by these nucleases is a major barrier to putative clinical applications. Furthermore, off-target mutagenesis by genome editing has not yet been addressed in pigs.

**Methods:**

Here, we generated genetically inheritable α-1,3-galactosyltransferase (*GGTA1*) knockout Yucatan miniature pigs by combining transcription activator-like effector nuclease (TALEN) and nuclear transfer. For precise estimation of genomic mutations induced by TALEN in *GGTA1* knockout pigs, we obtained the whole-genome sequence of the donor cells for use as an internal control genome.

**Results:**

In-depth whole-genome sequencing analysis demonstrated that TALEN-mediated GGTA1 knockout pigs had a comparable mutation rate to homologous recombination-treated pigs and wild-type strain controls. RNA sequencing analysis associated with genomic mutations revealed that TALEN-induced off-target mutations had no discernable effect on RNA transcript abundance.

**Conclusion:**

Therefore, TALEN appears to be a precise and safe tool for generating genome-edited pigs, and the TALEN-mediated *GGTA1* knockout Yucatan miniature pigs produced in this study can serve as a safe and effective organ and tissue resource for clinical applications.

## INTRODUCTION

Genome editing is known to permanently modify DNA sequences at a particular genomic site. Previously, such editing was achieved via a homologous recombination process using a DNA template containing long homologous arms extending to the target sequence at both sides, as well as host nucleases for induction and recovery of DNA breaks. Despite its high precision and specificity, homologous recombination is a time-consuming process that requires multiple steps and has limited efficiency in many mammalian cell types [1].

A breakthrough in genome editing occurred with the discovery of endonucleases that can specifically recognize and cleave target DNA sequences. The first endonuclease was zinc finger nuclease (ZFN). A method was devised based on two ZFNs engineered to recognize different DNA sequences located close to the target site, thus allowing simultaneous recognition and binding of both ZFNs. Therefore, ZFN appears to have an intrinsic benefit in limiting off-target mutagenesis [2]. The second endonuclease was transcription activator-like effector nuclease (TALEN), which is a fusion protein consisting of a bacterial TALE protein and FokI endonuclease [3]. It has a similar mechanism to ZFN, where TALE motifs are linked to FokI endonuclease, which requires dimerization for DNA cleavage to occur. This implies that the binding of two different TALENs at opposite strands in close vicinity to the target DNA is fundamental for TALEN activity. The third endonuclease-based genome editing method devised was the clustered regularly interspaced short palindromic repeats (CRISPR)/CRISPR-associated protein-9 nuclease (CRISPR-Cas9) system [4]. Recognition of the DNA site in the CRISPR-Cas9 system is controlled by RNA-DNA interactions. Compared to the previous endonuclease systems, CRISPR-Cas9 offers various advantages, such as facilitating guide-RNA design and off-target prediction. Furthermore, CRISPR-Cas9 allows simultaneous modification of several genomic sites.

Designing genome-editing endonucleases to reduce off-target mutagenesis has proven a major challenge not only in terms of basic scientific research, but also for putative clinical and industrial applications. A significant complication associated with these endonucleases is the binding of the nuclease to unintended genomic sites that share sequence homology with the on-target site. The delivery of ZFN, either *in vivo* or *in vitro*, can lead to toxicity or lethality due to binding at off-target sites and the induction of undesired DNA cleavage [5]. Both TALEN and CRISPR-Cas9 are known to provide relatively high target-site specificity. However, the single-guide RNAs in the CRISPR-Cas9 system can tolerate up to five mismatches with unintended target sites [6], and CRISPR-Cas9 itself has been shown to physically associate with many off-target sites in the genome [7]. In addition, several studies have reported very high levels of small insertion/deletion (indel) formation at unintended sites [6], although others suggest that initial estimates of off-target mutagenesis may have been overestimations [8,9]. By contrast, off-target activity appears to be less of an issue for TALEN. TALEN has an extremely long DNA binding site, and TALE motifs are expected to be rarely found in genomes. Consequently, there is little evidence of mismatch tolerance or off-target activity induced by TALEN [10].

Pigs are considered important source animals for agricultural applications and clinical xenotransplantation [11]. However, several concerns have been raised regarding pig-to-human immune incompatibility, and controlling the risk of cross-species transmission of infectious porcine diseases is a major challenge when using pigs, especially for organ xenotransplantation. Advances in genome editing in pigs have contributed to the attenuation of xenograft rejection and cross-species disease transmission. However, off-target mutagenesis by genome editing has not yet been addressed in this species.

In this study, we generated genetically inheritable α-1,3-galactosyltransferase (*GGTA1*) knockout Yucatan miniature pigs by combining TALEN and nuclear transfer. For precise estimation of genomic mutations induced by TALEN in *GGTA1* knockout pigs, we obtained the whole-genome sequence of the donor cells for use as an internal control genome. In-depth whole genome sequencing analysis demonstrated that TALEN-mediated *GGTA1* knockout pigs had comparable mutation rate as low as homologous recombination-treated pig and wild type strain controls. RNA sequencing analysis associated with genomic mutations also revealed that few TALEN-induced off-target mutations had no discernable effect on RNA transcript abundance. Therefore, TALEN appears to be a precise and safe method for generating genome-edited pigs, which could serve as source animals for organ xenotransplantation.

## MATERIALS AND METHODS

### Ethics statement

All animal procedures were carried out after approval of the Institutional Animal Care and Use Committee (IACUC) of Optipharm, Inc., Life Science Institute (IACUC approval No. OPT-140103-1).

### Generation of *GGTA1* knockout Yucatan miniature pigs by TALEN

TALE sequences and TALEN target site was identified by using CHOPCHOP, a web tool for selecting target sites for TALEN-directed mutagenesis (http://chopchop.cbu.uib.no) [12]. Exon 9 of porcine *GGTA1* gene (GenBank accession no. AH010595.2) was chosen for TALEN construct with 5′-GAG GAG TTC TTA A-3′ for left TALE, 5′-GGC CAC AAA GTC ATC TTT TAC A-3′ for right TALE. Constructed TALEN was transfected into primary ear skin fibroblasts of Yucatan male miniature pig at 3-month old using nucleofector, Amaxa-4D (Lonza, Köln, Cologne, Germany). After transfection, 1×10^4^ transfected cells were seeded in a 100-mm tissue culture plate (Corning, NY, USA) for single colony culture. Genomic DNA was extracted from each colony using DNeasy Blood and Tissue kit (Qiagen, Redwood City, CA, USA). Target sequence of TALEN in Exon 9 of *GGTA1* was amplified with 5′-AGA ATC ACC AGT CAG GTA AGC CAC TCC-3′ (forward primer) and 5′-TTG GAA GAC CTG ATC CAC GTC CAT GCA G-3′ (reverse primer), and the amplicon was subject to Sanger sequencing to analyze the mutagenesis in target site.

Oocyte preparation, nuclear transfer, embryo culture and embryo transfer were performed according to Choi et al [13]. Briefly, Cumulus-oocyte complexes were collected from prepubertal gilts at a local abattoir and maturated in TCM 199 medium (Gibco, Waltham,, MA, USA) supplemented with: 0.5 μg/mL luteinizing hormone, 0.5 μg/mL follicle stimulating hormone, 10 ng/mL epidermal growth factor, plus 0.1% polyvinyl alcohol, 3.05 mM D-glucose, 0.91 mM sodium pyruvate, 0.57 mM cysteine, 75 μg/mL penicillin G, and 50 μg/mL streptomycin) at 39°C with 5% CO_2_ air condition. After maturation, oocytes were freed from cumulus and enucleated by aspiration. TALEN-treated donor cells were cultured for 3 days in Dulbecco’s modified eagle medium supplemented with 0.5% fetal bovine serum, and a single donor cell was placed in the perivitelline space of an oocyte. Fusion and activation were induced simultaneously with two successive DC pulses of 1.1 kV/cm for 30 μs (BTX, Holliston, MA, USA). Reconstructed embryos were allowed for 1 or 2 days of additional culture in North Carolina State University (NCSU)-23 medium supplemented with 0.4% bovine serum albumin, and surgically transferred to the one lateral oviduct of a naturally cycling sow on the first day of standing estrus. Pregnancy status was monitored using an ultrasound scanner (Medison Co., Pangyo, Korea). F2 piglets of GGTA1 knockout pigs were produced by backcross between TALEN-mediated founder and F1 piglets. Ear skin fibroblasts were harvested from F2 piglets and wild type strain controls. The cells were stained with 1 μL of Alexa 488-conjugated isolectin GS-IB4 against (Invitrogen, Waltham, MA, USA) in 100 μL of Dulbecco’s phosphate-buffered saline (Gibco, USA) for 1 h at 4°C to detect alpha-gal epitope. After staining and washing, the cells were subject to flow cytometry analysis using FACSCallibur (Becton, Dickinson and Company, Franklin Lakes, NJ, USA).

### Whole genome sequencing

Genomic DNA was extracted from ear skin of *GGTA1* knockout pigs (CB1, CB3, CJ1) and wild type strain controls (WT1-3) using DNeasy Blood & Tissue Kit (Qiagen, USA). One microgram of genomic DNA sample was subjected to library preparation. Whole genome sequencing libraries with short inserts of 350 to 450 bp for paired-end reads were prepared using Truseq DNA sample prep kit (Illumina, San Diego, CA, USA) according to the manufacturer’s instruction. Whole genomic DNA was sequenced using Illumina HiSeq 2500 with an adaptation of the pairwise end-sequencing strategy. Post-sequencing analysis was performed using Theragen Etex’ workflow (www.theragenetex.com). Briefly, sequencing reads were aligned with Burrows-Wheeler aligner [14] with default parameters to The Swine Genome Sequencing Consortium Sscrofa 10.2 (https://doi.org/www.ncbi.nlm.nih.gov/assembly/GCF_000003025.5/). Alignments were sorted, merged and de-duplicated with SAMtools [15]. Genome Analysis Tool Kit was applied for variant calling with default parameters [16]. Variants were annotated by SnpEff [17] and depth of each base was calculated by BEDTools [18]. Genome-wide distribution of variant calls was visualized by using Circa (http://omgenomics.com/circa/).

### RNA sequencing

Total RNA was extracted from whole blood of *GGTA1* knockout pigs (CB1, CB3, CJ1) and wild type strain control (NC) using QIAamp RNA Blood Mini Kit (Qiagen, USA). RNA concentration and purity were determined using the Agilent 2100 BioAnalyzer (Agilent, Palo Alto, CA, USA), and RNA samples with RNA integrity number ≥7.0 were subject to library preparation. RNA sequencing libraries were prepared using 2 μg of total RNA as paired-end reads with a length of 100 bases using the TruSeq RNA Sample Preparation Kit (Illumina, USA). After quantification and qualification of the libraries using the Agilent 2100 BioAnalyzer (Agilent, USA) and the KAPA library quantification kit (Kapa Biosystems, Wilmington, MA, USA), flow cells were sequenced as paired-end reads (2×100 bp) using the Illumina HiSeq 2500 (Illumina, USA). Read filtering process and differentially expressed gene analysis were performed using Theragen Etex’ workflow (www.theragenetex.com). Briefly, after filtering out the low-quality reads, Trinity method was applied to assemble short reads [19], CD-HIT-EST to select the final unigenes from the assembled transcripts with identities of 94% [20]. Incomplete and redundant assembled transcripts were removed by selecting representative sequences [21]. Gene expression level was calculated based on fragments per kilobase of exon per million mapped reads (FPKM) using Cufflinks from The Swine Genome Sequencing Consortium Sscrofa 10.2. Differentially expressed genes were identified at a fold change cutoff of 2. Multiplex literature mining was performed using PubMatrix [22] with pair-wise comparisons of differentially expressed genes and modifier terms including nuclear transfer, lung, alveologenesis, and pig. False-positive findings were removed by manual curation.

## RESULTS

### Generation of TALEN-mediated *GGTA1* knockout Yucatan miniature pigs

**Figure 1:**
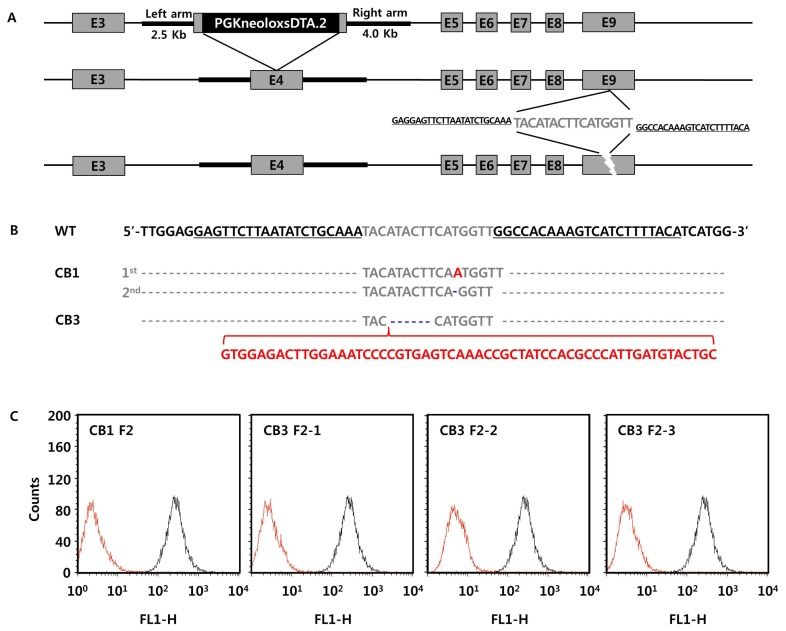
Generation of *GGTA1* knockout Yucatan miniature pigs. (A) Schematic diagrams of targeting pig *GGTA1* gene by homologous recombination (up) or by TALEN (down). Homologous recombination knockout vector contained 2.5 Kb left arm, knockout plasmid, and 4.0 Kb left arm. TALEN vector contained 20-mer TALE at each side (underlined). (B) Sequencing results of TALEN-mediated *GGTA1* knockout pigs. Underlined sequences in wild type control (WT) indicate TALE sequences, gray characters indicate target sequence. Red characters indicate inserted sequences, and blue deleted sequences in TALEN-treated pigs (CB1 and CB3). (C) Expression analyses of GGTA1 protein in F1 generation of TALEN-treated pigs by flow cytometry. Red lines indicate GGTA1 expression in TALEN-treated pigs, black lines indicate those in wild type strain control. *GGTA1*, α-1,3-galactosyltransferase; TALEN, transcription activator-like effector nuclease.

Heterozygous *GGTA1* knockout pig ear skin fibroblasts were generated by homologous recombination at exon 4 of the gene, and used to generate *GGTA1* knockout pigs by nuclear transfer (piglet ID = CJ1) [13]. The same pig ear skin fibroblast clones were subjected to TALEN treatment for mutation of exon 9 (Figure 1A). After verification of genetic interruption and reduced expression of GGTA1 protein in the TALEN-mediated pig ear skin fibroblasts, the cells were used as donor cells for nuclear transfer, and reconstructed embryos were transferred to recipient miniature pigs. After full-term development, TALEN-mediated *GGTA1* knockout piglets were successfully delivered (piglet IDs = CB1 and CB3). Sequencing analysis of genomic DNA isolated from the ear skin of the TALEN-mediated *GGTA1* knockout piglets demonstrated that CB1 had various mutant genotypes, such as a single base (A) insertion at chr1:293631881 and a single base deletion (T) at chr1:293631880 in exon 9 of *GGTA1* gene. In CB3, there was a 6-base deletion (ATA CTT) and a 47-base insertion (GTG GAG ACT TGG AAA TCC CCG TGA GTC AAA CCG CTA TCC ACG CCC ATT GAT GTA CTG C) at chr1: 293631888 – chr1:293631883 in exon 9 of the *GGTA1* gene (Figure 1B). The inserted sequence in CB3 was not identified by the National Center for Biotechnology Information Basic Local Alignment Search Tool (https://doi.org/blast.ncbi.nlm.nih.gov/Blast.cgi). Small indels occurred between the left and right TALE sequences. To confirm germline transmission of the genetic traits of the TALEN-mediated *GGTA1* knockout pigs, one F2 offspring of CB1 (piglet ID = CB1 F2) and three F2 offspring of CB3 (piglet IDs = CB F2-1 to 3) were produced by backcross. Flow cytometry analysis showed that the expression levels of GGTA1 protein in fibroblasts derived from the ear skin of F2 piglets were significantly lower than those of the wild-type strain control (Figure 1C). These results demonstrated that TALEN is an efficient and precise genome-editing method to modulate target gene activity, and that TALEN-mediated gene mutations are heritable to the next generation.

### Genomic mutation profile in *GGTA1* knockout Yucatan miniature pigs

**Figure 2:**
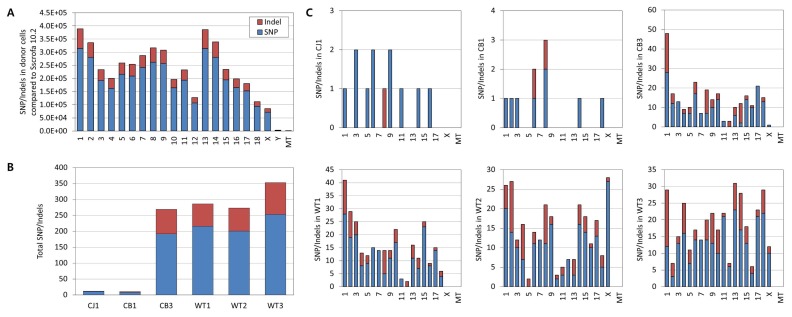
Chromosomal frequency of genomic mutations. (A) Frequency of SNPs and indels in donor cell in comparison with porcine reference genome Sscrofa 10.2. (B) Total SNPs and indels found in individual pigs in comparison with donor cells. (C) Chromosomal frequency of SNPs and indels in individual pigs in comparison with donor cells. CJ1 indicates homologous recombination-edited *GGTA1* knockout pig. CB1 and CB3 indicate TALEN-mediated *GGTA1* knockout pigs. Red stacked bars indicate indel frequency and blue bars SNPs. MT means mitochondrial genome. SNP, single nucleotide polymorphism; *GGTA1*, α-1,3-galactosyltransferase; TALEN, transcription activator-like effector nuclease.

Although *GGTA1* was successfully disrupted by TALEN, we could not exclude the possibility of off-target mutations in genetic elements not targeted by TALEN. To assess this, we performed whole-genome sequencing to determine the presence of indels and single nucleotide polymorphisms (SNPs) in the TALEN-mediated *GGTA1* knockout pigs. We first compared the whole-genome sequence of donor cells to the pig reference genome Sscrofa 10.2, and found 802,662 indels and 3,878,732 SNPs throughout the chromosomes (Figure 2A). The unexpectedly high frequency of genomic mutations in the donor cells implied that the pig reference genome would be inappropriate for identification of TALEN-induced, true off-target mutations by sequence comparison. Therefore, we determined the whole-genome sequence of the donor cells for use as an internal control genome, to obtain a precise estimation of genomic mutations induced by TALEN in *GGTA1* knockout pigs.

Mutation profiling using the internal control genome revealed that TALEN-mediated *GGTA1* knockout pigs had few indels and SNPs, with numbers comparable to the homologous recombination-treated knockout pig and the wild-type strain controls (Figure 2B). The average sequencing depth was 230× for TALEN-treated pigs, 200× for homologous recombination-treated pigs, and 204× for wild-type controls, which confirmed the accuracy of the whole-genome sequencing analyses. The variant calling pipeline revealed that the TALEN-treated pig CB1 harbored one base insertion (T) at chr8: 33762665, one base deletion (T) at chr6:81281307, and eight SNPs, while CB3 had 70 indels and 192 SNPs. No off-target mutations induced by TALEN were predicted by CHOPCHOP [12] in the basis of Sscrofa 10.2. Compared to the TALEN-treated pigs, the homologous recombination-treated pig CJ1 had a single deletion (T) at chr8: 33762665 and 11 SNPs, while the wild-type controls had an average of 80 indels and 223 SNPs. The mutation rates detected in the TALEN-mediated *GGTA1* knockout pigs were similar or higher than those in the homologous recombination-treated pig, but lower than the frequency of spontaneous germline variants observed in wild-type strain controls.

The genomic mutations were distributed evenly throughout the chromosomes in TALEN- and homologous recombination-treated pigs, and were comparable to those of germline variants in wild-type strain controls (Figure 2C). Among the 10 genomic mutations found in CB1, one indel and two SNPs were in intron regions of protein-coding genes. Of the 20 indels in CB3, two were found in the upstream region, one in the 3′ untranslated region (UTR), and 17 in the intron region. Out of 59 SNPs, six were located in the upstream region, one in the 3′ UTR, and 52 in the intron regions of protein-coding genes. Similarly, SNPs detected in CJ1 were located in the intron regions of protein-coding genes. None of the mutations in the TALEN- or homologous recombination-treated pigs were expected to have deleterious effects (Table 1, Supplementary Tables S1–6). Taken together, the genomic analysis of *GGTA1* knockout pigs demonstrated that an internal control genome is important for precise detection of true genomic mutations due to genome editing, and that TALEN might not induce excessive off-target mutations, with frequencies comparable to those associated with homologous recombination.

### Differentially expressed genes in *GGTA1* knockout Yucatan miniature pigs

As described above, TALEN-induced mutations in *GGTA1* knockout pigs were not excessive compared to the internal control genome, and most of the mutations were located in non-coding regions of various genes; however, this does not exclude the possibility of unintended effects of TALEN-induced mutations on the physiological and genomic integrity of *GGTA1* knockout pigs. To address this issue, we performed transcriptomic analysis of *GGTA1* knockout pigs, and identified differentially expressed genes in those pigs by comparison with the wild-type strain control.

**Figure 3:**
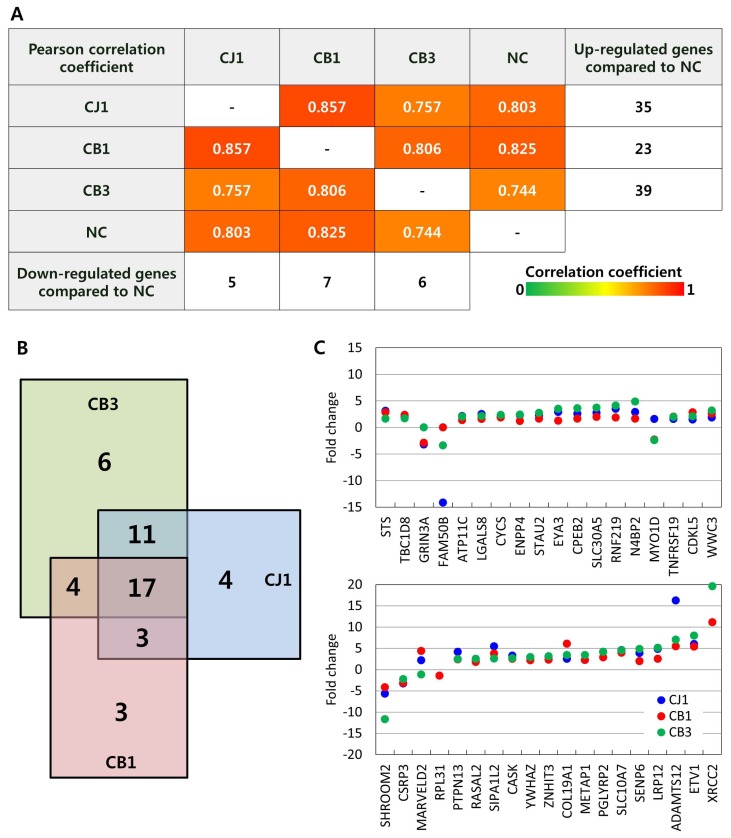
Global gene expression of *GGTA1* knockout Yucatan miniature pigs. (A) Pearson correlation coefficient among expression profiles of *GGTA1* knockout pigs and wild type strain control (NC). (B) Venn Diagram of differentially expressed genes in each *GGTA1* knockout pigs compared to wild type strain control. Red square indicates differentially expressed genes observed in CB1, green in CB3, and blue in CJ1. (C) Fold changes of differentially expressed genes (fold change ≥2) observed in individual *GGTA1* knockout pigs. Blue dots indicate differentially expressed genes in CJ1, red in CB1, and green in CB3 compared to wild type strain control. SNP, single nucleotide polymorphism; *GGTA1*, α-1,3-galactosyltransferase.

Expression values of an average of 56,038 transcripts from 22,852 genes were obtained from *GGTA1* knockout and wild-type pigs (Supplementary Table S7). Correlation analysis of the expressed transcripts showed that the global gene expression profiles of individual knockout pigs and the wild-type pig were well-correlated (correlation coefficient of 0.744 to 0.857; Figure 3A). In addition, few genes were differentially expressed in *GGTA1* knockout pigs compared to the wild-type pig (fold change ≥2). CB1 had 23 up- and seven down-regulated genes compared to the wild-type pig. Likewise, CB3 had 39 up- and six down-regulated genes. The numbers of differentially expressed genes in the TALEN-mediated *GGTA1* knockout pigs were comparable to those in the homologous recombination-treated CJ1 pig, which had 35 up- and five down-regulated genes.

We compared the differentially expressed genes of the individual knockout pigs, and determined that three, six, and four genes were exclusively expressed in CB1, CB3, and CJ1, respectively. In contrast, 35 genes were commonly expressed in *GGTA1* knockout pigs (Figure 3B), including Shroom family member 2 (*SHROOM2*), cysteine and glycine rich protein 3, and MARVEL domain-containing 2 (*MARVELD2*) (Figure 3B). When we compared the fold-changes of the genes commonly differentially expressed in *GGTA1* knockout pigs, most showed similar directions of change (up- or down-regulation) compared to the wild-type pig (Figure 3C). Literature mining revealed that several differentially expressed genes in *GGTA1* knockout pigs are associated with DNA methylation in gene regulation, including family with sequence similarity 50, member B (*FAM50B*) [23,24], cytochrome C, somatic [25], *SHROOM2* [26], protein tyrosine phosphatase, non-receptor type 13 (*PTPN13*) [27], and LDL receptor-related protein 12 (*LRP12*) [28]. Other differentially expressed genes are known to be involved in respiratory diseases, such as lung cancer, including *FAM50B* [24], ATPase phospholipid transporting 11C [29], WWC family member 3 [30–32], *PTPN13* [27], RAS protein activator-like 2 [33], tyrosine 3-monooxygenase/tryptophan 5-monooxygenase activation protein zeta [34,35], and *LRP12* [28]. Dysregulated DNA methylation and lung pathophysiology are well known abnormalities in pigs cloned by somatic cell nuclear transfer [36]. Therefore, it appears that the differential gene expression in *GGTA1* knockout pigs is caused by the nuclear transfer procedure itself, rather than off-target effects of TALEN.

### Gene expression associated with genomic mutations in *GGTA1* knockout miniature pigs

**Figure 4:**
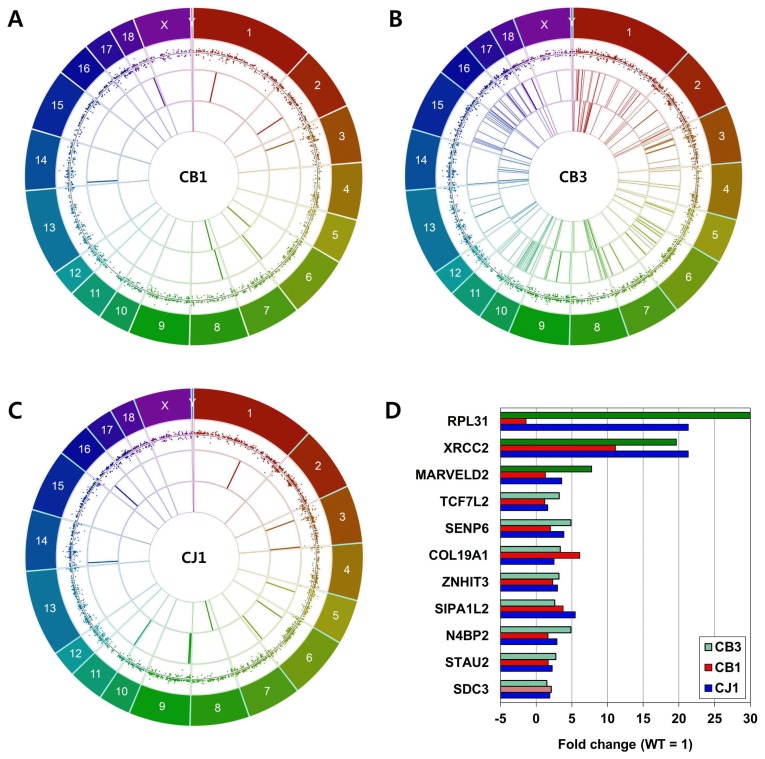
Correlation between genomic mutations and gene expression. (A) Circos plot of CJ1, (B) CB1, and (C) CB3. Outer layers indicate pig reference genome Sscrofa 10.2. First inner layers indicate differentially expressed transcripts compared to wild type strain control (log2|fold change| ≥2). Horizontal lines indicate fold change = 1. Second inner layers indicate SNPs, and third inner layers indels. Quality of read sequencing ≥1,000, and total depth ≥100. (D) Gene expression associated with genomic mutations. Bars indicate fold change of gene expression compared to wild type control. Blue bars indicate relative gene expression levels in CJ1, red in CB1, and green in CB3. Expression levels of genes harboring indel were depicted in light red or light green bars, SNP in deep green bars. SNP, single nucleotide polymorphism.

To obtain insight into the association between TALEN-induced genomic mutations and gene expression, we generated multi-layer Circos plots with differentially expressed transcripts, SNPs, and indels. *GGTA1* knockout pigs produced by TALEN had 1,873 differentially expressed transcripts covering seven annotated genes (CB1), and 3,722 transcripts with 16 annotated genes (CB3) (log2|fold change|≥2). In addition, CB1 had eight SNPs and two indels, while CB3 had 192 SNPs and 70 indels. All of the differentially expressed transcripts, SNPs, and indels from TALEN-mediated *GGTA1* knockout pigs were distributed evenly throughout the genome (Figure 4A, B). Likewise, the *GGTA1* knockout pig produced by homologous recombination had 3,142 differentially expressed transcripts covering 14 annotated genes, 11 SNPs, and one indel, which were distributed evenly throughout the genome (Figure 4C). Among the genomic mutations, one SNP (c.376 +331C>T) at chr3:33134141 in the intron region of ENSSSCT 00000008657 was common to both CB3 and CJ1, and one insertion (c.820-502_820-501insT) at chr6:81281307 in the intron region of syndecan-3 (*SDC3*) was common to CB1 and CB3. There were no other common genomic mutations among the *GGTA1* knockout pigs.

Next, we investigated the possible effects of genomic mutations on gene expression by comparing the expression levels of protein-coding genes harboring SNPs or indels with those of intact genes (Figure 4D). As described above, CB1 and CB3 shared a single base insertion in the intron region of *SDC3*; their expression levels were comparable to that of *SDC3* in CJ1, which had no mutation in that region. Likewise, CB3 had indels in non-coding regions of Staufen double-stranded RNA-binding protein 2, NEDD4-binding protein 2, signal-induced proliferation-associated 1-like 2, zinc finger HIT-type containing 3, collagen type XIX alpha 1 chain, SUMO-specific peptidase 6, and transcription factor 7-like 2; the expression levels of these genes were comparable to those in CB1 and CJ1, which had no genomic mutations in these genetic elements. Furthermore, no differences in expression levels were observed among genes harboring SNPs in non-coding regions in CB3 compared to CB1 and CJ1, including *MARVELD2*, X-ray repair cross-complementing 2, and ribosomal protein L31. These results imply that TALEN-induced off-target mutations in *GGTA1* knockout pigs had no discernable effects on RNA transcript abundance.

## DISCUSSION

Whole-genome sequencing of TALEN-mediated *GGTA1* knockout Yucatan miniature pigs revealed numerous SNPs and indels compared to the pig reference genome Sscrofa 10.2. However, it is questionable whether the pig reference genome is appropriate for identifying true TALEN-induced mutations. Recently, a comparative genomic analysis of pigs by Kim et al [37] revealed that Yucatan miniature pigs have the largest genetic distance from conventional pig breeds, including Yorkshire, Landrace, and Duroc. In addition, genes involved in protein amino acid phosphorylation, cell proliferation, microtubule-based processes, and the cell cycle show haplotype diversity in Yucatan miniature pigs, especially compared to the Duroc breed. The pig reference genome Sscrofa 10.2 was built based on the Duroc breed (https://doi.org/www.ncbi.nlm.nih.gov/assembly/GCF_000003025.5/), and therefore it is reasonable to suspect that the unexpected mutations may originate from strain differences rather than TALEN treatment. Furthermore, an emerging body of evidence indicates that proper references should be adopted, that is, parental lines when available, to identify genome editing-induced mutations [38–40].

To overcome false discovery and the limited applicability of the pig reference genome, we generated an internal reference genome from donor cells of the Yucatan miniature pig. This approach revealed that most of the observed mutations were due to strain differences, and not to TALEN treatment, and few putative mutations induced by TALEN in non-coding regions of genes were identified. The *GGTA1* knockout pigs were produced using two different methods: TALEN and homologous recombination. Homologous recombination is a well-established gene targeting technique considered as the gold standard for generating genetically engineered animals, due to its highly precise genome editing [1]. In this study, the mutation frequency in TALEN-mediated pigs was comparable to that of homologous recombination-treated pigs. Furthermore, we used wild-type strain controls for genome comparison, and revealed that the off-target mutations found in TALEN-treated pigs were within *de novo* variants of the Yucatan breed. Therefore, these results demonstrated that the use of an internal control genome from the parental line is effective for precisely estimating genome editing-induced off-target mutations, and TALEN editing can precisely edit the genome without introducing excessive, unintended, off-target mutations.

Although the mutation frequency for TALEN was comparable to those for homologous recombination and germline variants, there were still large differences in the SNPs and indels observed in individual TALEN-mediated pigs. We used the same donor cells from Yucatan miniature pigs at different passages to generate *GGTA1* knockout pigs; therefore, the variability may have originated from spontaneous mutations accumulated during donor cell culture, or from the uneven nature of TALEN-induced mutagenesis. It is known that somatic mutations can continuously occur in normal cells and contribute to the generation of a lineage-specific genomic signature [41]. In addition, mutation processes are subject to different biological influences. For example, growing cells at extremely low cell density resulted in an enhanced mutation rate [42], and the cell lineage or cell division rate could also influence the mutation rate [41]. In this study, we used donor cells derived from pig ear skin fibroblasts, which have a limited capacity for cell division. Therefore, we cannot exclude the possibility that incomplete culture conditions induced the accumulation of sporadic somatic mutations, leading to genomic heterogeneity and resulting in interruption of the intrinsic cellular program for survival and proliferation.

It is also well known that the degree of mutagenesis induced by genome editing can differ, especially the frequency of single nucleotide variants. Consequently, some cell clones might be at higher risk of off-target mutations [43]. In this study, the TALEN-treated pig CB3 had a higher number of variants (70 indels and 192 SNPs) compared to CB1 (2 indels and 8 SNPs), and these animals were generated using independent TALEN-treated donor cells. Therefore, it appears that TALEN may induce different degrees of mutagenesis during independent genome editing processes. In combination with spontaneous induction of heterogeneity, differential sensitivity to TALEN-induced mutagenesis may result in large differences in the genetic background of the donor cells. However, consistent with previous reports, the incidence of likely off-target mutations induced by TALEN in *GGTA1* knockout pigs was sufficiently low to not be of significant concern for disease modeling and other applications.

TALEN is recognized for its high degree of precision and control, while CRISPR-Cas9 is renowned for its simple and robust genome editing. Consequently, several clinical trials using TALEN or CRISPR-Cas9 as standalone therapies, or as an integral part of another application, such as chimeric antigen receptor-T (CAR-T) therapy, are underway [44]. For example, clinical treatments of HPV-related cervical intraepithelial neoplasia (NCT03057912) and cervical precancerous lesions (NCT03226470) using TALEN are currently being investigated. Using CRISPR-Cas9, studies of programmed cell death 1 knockout engineered T cells for advanced esophageal cancer (NCT03081715) or metastatic non-small cell lung cancer (NCT02793856), CD19 and CD20 or CD22 dual-specific CAR-T cell immunotherapy for relapsed or refractory leukemia and lymphoma (NCT03398967), universal CART019 for patients with relapsed or refractory CD19-positive leukemia and lymphoma (NCT03166878), and transplantation of C-C motif chemokine receptor 5-modified CD34-positive cells for HIV-infected subjects with hematological malignances (NCT03164135) are in progress (https://doi.org/clinicaltrials.gov).

Therefore, it appears that because of its simplicity and robustness, CRISPR-Cas9 has overtaken TALEN as the leading genome editing therapy. However, there is still some controversy about the use of CRISPR-Cas9 as a standalone therapy due to uncertainty regarding its effects on genomic integrity, despite all the clinical trials that have been performed. Regarding off-target mutagenesis, TALEN usually covers up to 40 bases, which provides higher specificity for genome editing. CRISPR-Cas9, on the other hand, recognizes 20 bases, increasing the likelihood of off-target effects [45,46]. It is well-established that CRISPR-Cas9 does not induce excessive off-target small indels. Nonetheless, large deletions and more complex genomic rearrangements at the targeted sequence by CRISPR-Cas9 have been observed, which raises concerns regarding the possible pathogenic consequences of CRISPR-Cas9 in clinical applications [47]. In this study, we analyzed TALEN-mediated pig genomes in great depth (>200× coverage), and confirmed the consistent specificity of TALEN and rareness of off-target mutagenesis, with no pathogenic consequences. Therefore, TALEN appears to be a precise and safe tool for generating genome-edited pigs, which could serve as source animals for xenotransplantation approaches.

Pigs have genetic, anatomical, and physiological similarities to humans, and can be bred in large numbers [48]. Moreover, biomedical use of pigs is relatively free from ethical issues. For these reasons, pigs are considered an important animal species not only as an agricultural resource, but also for biomedical applications. Particularly for clinical applications, pigs are considered to be the most suitable source animals for xenotransplantation, to address the lack of human organs for clinical transplantation [11]. However, there are several concerns associated with using pigs for xenotransplantation of organs. First, immunological incompatibility between the pig organ and the human recipient is a major challenge. Second, the risk of cross-species transmission of porcine infectious disease acts as another barrier.

The main reason for xenograft failure is the hyperacute immune response against alpha-galactosyl epitopes on the surface of porcine endothelial cells caused by GGTA1-mediated catabolism [49]. Therefore, intensive research has been carried out to generate *GGTA1* knockout pigs [50–53], and the graft survival period after xenotransplantation using *GGTA1* knockout pigs has been significantly extended in non-human primates [54,55]. Besides, The transmission of porcine endogenous retroviruses (PERVs) is another major concern [56]. Complete elimination of PERVs is very challenging because they are integrated in many locations of the porcine genome. The recent production of PERV knockout pigs, achieved by combining genome editing and nuclear transfer, alleviates this safety concern [57]. Thus, advances in genome editing in pigs should help to attenuate xenograft rejection and cross-species disease transmission. All of these achievements point to the likelihood of significant further advances in xenotransplantation within the next few years.

In conclusion, this study demonstrated that TALEN is a precise and safe method to generate genome-edited pigs without excessive off-target mutations and potentially deleterious effects. Most importantly, the TALEN-mediated *GGTA1* knockout Yucatan miniature pigs produced in this study can serve as safe and effective animal organ and tissue resources for clinical applications.

## Supplementary Data















## Figures and Tables

**Table 1:** Summary of indels in coding genes in *GGTA1* knockout Yucatan miniature pigs

Sample	Chr	Pos	Ref	Alt	Quality	Total depth	Effect	Codon	Gene symbol
CJ1	------------------------------------------------------------------------------ Not found ----------------------------------------------------------------------------------								
CB1	6	81281307	G	GT	2,736.0	183	Intron variant	c.820-502_820-501insT	*SDC3*
CB3	1	109000000	C	CA	1,282.4	229	Intron variant	c.16+1468_16+1469insT	*ACAA2*
CB3	1	56505569	AT	A	2,632.1	212	Intron variant	c.2398-1185delT	*COL19A1*
CB3	1	101000000	CA	C	1,316.4	185	Intron variant	c.151-2454delT	*SENP6*
CB3	1	106000000	ATT	A	1,997.0	186	Upstream gene variant	c.-160_-160delAA	ENSSSCG00000021219
CB3	2	67265991	AT	A	3,801.1	236	3′UTR variant	c.*243delT	ENSSSCG00000013715
CB3	4	67994103	TTG	T	1,837.9	214	Intron variant	c.814+7317_814+7318delTG	*STAU2*
CB3	5	9089370	C	CT	2,230.0	216	Intron variant	c.3101-81_3101-80insA	*MYH9*
CB3	6	81281307	G	GT	2,736.0	183	Intron variant	c.820-502_820-501insT	*SDC3*
CB3	8	32890209	C	CT	1,085.9	213	Intron variant	c.406+1180_406+1181insT	*N4BP2*
CB3	8	39555553	T	TTA	4,723.5	227	Intron variant	c.2411+1756_2411+1757insTA	*CORIN*
CB3	8	39652185	GAGA	G	1,633.5	200	Intron variant	c.1012+13959_1012+13961delTCT	*CORIN*
CB3	8	138000000	AT	A	2,739.7	169	Intron variant	c.1510-27278delA	ENSSSCG00000022986
CB3	8	138000000	A	ATG	4,606.9	212	Intron variant	c.1509+897_1509+898insCA	ENSSSCG00000022986
CB3	9	100000000	T	TGG	1,325.4	223	Intron variant	c.11403+301_11403+302insGG	*DNAH11*
CB3	9	100000000	CTG	C	2,495.5	210	Intron variant	c.6902+405_6902+406delTG	*DNAH11*
CB3	12	39592974	T	TC	1,977.2	262	Upstream gene variant	c.-87_-87insG	*ZNHIT3*
CB3	13	403773	C	CA	3,243.7	202	Intron variant	c.180+44121_180+44122insA	ENSSSCG00000023343
CB3	14	62454044	GT	G	2,509.5	237	Intron variant	c.3350+733delA	*SIPA1L2*
CB3	14	135000000	T	TA	1,830.7	235	Intron variant	c.93+37916_93+37917insA	*TCF7L2*
CB3	18	40749240	G	GA	1,047.3	198	Intron variant	c.-141+11760_-141+11761insA	*ELMO1*

*GGTA1*, α-1,3-galactosyltransferase; *SDC3*, syndecan-3; *ACAA2*, acetyl-CoA Acyltransferase 2; *COL19A1*, collagen type XIX alpha 1 chain; *SENP6*, SUMO-specific peptidase 6; *STAU2*, Staufen double-stranded RNA-binding protein 2; *MYH9*, myosin heavy chain 9; *N4BP2*, NEDD4-binding protein 2; *CORIN*, corin, serine peptidase; *DNAH11*, dynein axonemal heavy chain 11; *ZNHIT3*, zinc finger HIT-type containing 3; *SIPA1L2*, signal-induced proliferation-associated 1-like 2; *TCF7L2*, transcription factor 7-like 2; *ELMO1*, engulfment and cell motility 1.
