# Supplementary material for: No excessive mutations in transcription activator-like effector nuclease-mediated α-1,3-galactosyltransferase knockout Yucatan miniature pigs
Source: Asian-Australas J Anim Sci. 2019 Aug 23;33(2):360–72. doi: 10.5713/ajas.19.0480 (PMC6946973; doi:10.5713/ajas.19.0480)
Supplement: Supplementary file 1 [file ajas-19-0480-suppl1.pdf]

Supplementary Table 1. Variant calls of TALEN-edited pig CJ1

#CHROM	POS	ID	REF	ALT	QUAL	FILTER	DP	Donor	CJ1	CB1	CB3	WT1	WT2	WT3	EFF[*].EFFECT	EFF[*].IMPACT	EFF[*].FUNCLASS	EFF[*].CODON	EFF[*].AA	EFF[*].AA_LEN	EFF[*].GENE	EFF[*].BIOTYPE	EFF[*].CODING	EFF[*].TRID	EFF[*].RANK
1	190448139		A	G	1167.65		122	0/0	1/1	0/1	1/1	1/1	1/1	1/1	intergenic_	MODIFIER	NONE			-1	5S_rRNA-U6				-1
3	33134141		C	T	1105.93		145	0/0	1/1	0/1	1/1	1/1	0/1	1/1	intron_vari	MODIFIER	NONE	c.376+331C>T		-1	ENSSSCG00000007901	protein_coding		ENSSSCT0	5
3	142488536		A	C	1213.04		702	1/1	0/0	./.	1/1	1/1	1/1	./.	intergenic_	MODIFIER	NONE			-1	ACP1-ENSSSCG00000008661				-1
5	28871833		A	G	1001.13		160	1/1	0/0	1/1	1/1	1/1	0/1	1/1	intergenic_	MODIFIER	NONE			-1	U6-ENSSSCG00000000457				-1
6	29004053		G	C	1151.74		119	1/1	0/0	1/1	1/1	1/1	1/1	1/1	intergenic_	MODIFIER	NONE			-1	ENSSSCG00000002834-TOX3				-1
6	145141339		C	T	1024.87		151	1/1	0/0	0/0	1/1	1/1	0/1	1/1	intergenic_	MODIFIER	NONE			-1	PPAP2B-ENSSSCG00000024345				-1
8	33762665		GT	G	1159.43		216	0/0	1/1	1/1	1/1	1/1	1/1	0/1	intergenic_	MODIFIER	NONE			-1	NSUN7-U6				-1
9	4683739		A	G	1065.02		152	0/0	1/1	0/1	1/1	1/1	1/1	1/1	intergenic_	MODIFIER	NONE			-1	ENSSSCG00000014673-TRIM6				-1
9	10086967		A	G	1858.4		186	1/1	0/0	1/1	1/1	1/1	1/1	1/1	intron_vari	MODIFIER	NONE	c.98-701T>C		-1	CHRDL2	protein_coding		ENSSSCT0	1
11	2234773		T	C	1143.95		132	1/1	0/0	0/1	1/1	1/1	0/0	1/1	intron_vari	MODIFIER	NONE	c.55+21048A>G		-1	ENSSSCG00000009293	protein_coding		ENSSSCT0	1
14	11250816		G	T	1477.15		177	0/0	1/1	1/1	0/1	0/1	1/1	1/1	intergenic_	MODIFIER	NONE			-1	ENSSSCG00000009655-PPP2R2A				-1
16	27256330		C	T	1007.81		138	1/1	0/0	0/1	0/1	1/1	1/1	1/1	intron_vari	MODIFIER	NONE	c.-23-263G>A		-1	C6	protein_coding		ENSSSCT0	2

Head	Note
#CHROM	Chromosome
POS	Position
ID	Identification
REF	Reference seq (Sscrofa 10.2)
ALT	Alternative sequence
QUAL	Quality
FILTER	
DP	Total depth
EFF[*].EFFE	Genetic element
EFF[*].IMP	Functional annotation
EFF[*].FUNCLASS	
EFF[*].CODON	
EFF[*].AA	
EFF[*].AA_LEN	
EFF[*].GENE	
EFF[*].BIOTYPE	
EFF[*].CODING	
EFF[*].TRID	
EFF[*].RANK	
./.	Not called
0/0	Homogeneous to REF
0/1	Heterogeneous to REF
1/1	Homogeneous to ALT
