# Supplementary material for: No excessive mutations in transcription activator-like effector nuclease-mediated α-1,3-galactosyltransferase knockout Yucatan miniature pigs
Source: Asian-Australas J Anim Sci. 2019 Aug 23;33(2):360–72. doi: 10.5713/ajas.19.0480 (PMC6946973; doi:10.5713/ajas.19.0480)
Supplement: Supplementary file 2 [file ajas-19-0480-suppl2.pdf]

Supplementary Table 2. Variant calls of TALEN-edited pig CB1

#CHROM	POS	ID	REF	ALT	QUAL	FILTER	DP	Donor	CJ1	CB1	CB3	WT1	WT2	WT3	EFF[*].EFFECT	EFF[*].IMPACT	EFF[*].FUNCLASS	EFF[*].CODON	EFF[*].AA	EFF[*].AA_LEN	EFF[*].GENE	EFF[*].BIOTYPE	EFF[*].CODING	EFF[*].TRID	EFF[*].RANK
1	91512902		T	A	1472.75		101	1/1	./.	0/0	1/1	1/1	1/1	1/1	intergenic_	MODIFIER	NONE			-1	HS3ST5-FRK				-1
2	100139787		C	A	1181.05		1467	1/1	1/1	0/0	0/1	1/1	1/1	1/1	intron_vari	MODIFIER	NONE	c.38+3267C>A		-1	ENSSSCG00000023484	protein_coding		ENSSSCT0	3
3	36315111		A	G	1089.21		132	1/1	0/1	0/0	1/1	1/1	0/1	1/1	intergenic_	MODIFIER	NONE			-1	RBFOX1-U6				-1
6	81281307		G	GT	2735.98		183	0/0	0/1	1/1	1/1	0/1	0/1	1/1	intron_vari	MODIFIER	NONE	c.820-502_820-501ins		-1	SDC3	protein_coding		ENSSSCT0	2
6	145141339		C	T	1024.87		151	1/1	0/0	0/0	1/1	1/1	0/1	1/1	intergenic_	MODIFIER	NONE			-1	PPAP2B-ENSSSCG00000024345				-1
8	21945383		A	G	1116.71		139	0/0	0/1	1/1	1/1	1/1	0/1	1/1	intergenic_	MODIFIER	NONE			-1	7SK-ENSSSCG00000008763				-1
8	21945384		T	C	1257.37		140	0/0	0/1	1/1	1/1	1/1	1/1	1/1	intergenic_	MODIFIER	NONE			-1	7SK-ENSSSCG00000008763				-1
8	33762665		GT	G	1159.43		216	0/0	1/1	1/1	1/1	1/1	1/1	0/1	intergenic_	MODIFIER	NONE			-1	NSUN7-U6				-1
14	11250816		G	T	1477.15		177	0/0	1/1	1/1	0/1	0/1	1/1	1/1	intergenic_	MODIFIER	NONE			-1	ENSSSCG00000009655-PPP2R2A				-1
18	55245294		T	C	1405.56		122	0/0	0/1	1/1	0/0	0/1	1/1	1/1	intron_vari	MODIFIER	NONE	c.273+8194A>G		-1	CCM2	protein_coding		ENSSSCT0	3

Head	Note
#CHROM	Chromosome
POS	Position
ID	Identification
REF	Reference seq (Sscrofa 10.2)
ALT	Alternative sequence
QUAL	Quality
FILTER	
DP	Total depth
EFF[*].EFFE	Genetic element
EFF[*].IMP	Functional annotation
EFF[*].FUNCLASS	
EFF[*].CODON	
EFF[*].AA	
EFF[*].AA_LEN	
EFF[*].GENE	
EFF[*].BIOTYPE	
EFF[*].CODING	
EFF[*].TRID	
EFF[*].RANK	
./.	Not called
0/0	Homogeneous to REF
0/1	Heterogeneous to REF
1/1	Homogeneous to ALT
