# Supplementary material for: No excessive mutations in transcription activator-like effector nuclease-mediated α-1,3-galactosyltransferase knockout Yucatan miniature pigs
Source: Asian-Australas J Anim Sci. 2019 Aug 23;33(2):360–72. doi: 10.5713/ajas.19.0480 (PMC6946973; doi:10.5713/ajas.19.0480)
Supplement: Supplementary file 3 [file ajas-19-0480-suppl3.pdf]

Supplementary Table 3. Variant calls of TALEN-edited pig CB3

#CHROM	POS	ID	REF	ALT	QUAL	FILTER	DP	Donor	CJ1	CB1	CB3	WT1	WT2	WT3	EFF[*].EFFECT	EFF[*].IMPACT	EFF[*].FUNCLASS	EFF[*].CODON	EFF[*].AA	EFF[*].AA_LEN	EFF[*].GENE	EFF[*].BIOTYPE	EFF[*].CODING	EFF[*].TRID	EFF[*].RANK
1	108868252		C	CA	1282.4		229	0/0	0/0	0/0	1/1	0/1	1/1	1/1	upstream_	MODIFIER	NONE	n.-1_-1insA		-1	SCARNA17	snoRNA		ENSSSCT0	-1
1	108868252		C	CA	1282.4		229	0/0	0/0	0/0	1/1	0/1	1/1	1/1	upstream_	MODIFIER	NONE	n.-1_-1insA		-1	SCARNA18	snoRNA		ENSSSCT0	-1
1	108868252		C	CA	1282.4		229	0/0	0/0	0/0	1/1	0/1	1/1	1/1	intron_vari	MODIFIER	NONE	c.16+1468_16+1469ir		-1	ACAA2	protein_coding		ENSSSCT0	1
1	181967325		T	C	4023.92		210	0/0	0/1	0/1	1/1	1/1	1/1	1/1	intergenic_	MODIFIER	NONE			-1	U2-DIS3L				-1
1	190448139		A	G	1167.65		122	0/0	1/1	0/1	1/1	1/1	1/1	1/1	intergenic_	MODIFIER	NONE			-1	5S_rRNA-U6				-1
1	4331864		GT	G	2639.01		226	1/1	1/1	1/1	0/0	0/1	1/1	1/1	intergenic_	MODIFIER	NONE			-1	U6-ENSSSCG00000029213				-1
1	12888936		A	C	1187.68		143	1/1	1/1	1/1	0/0	0/1	1/1	1/1	intergenic_	MODIFIER	NONE			-1	5S_rRNA-TFB1M				-1
1	12895941		C	T	1841.66		153	1/1	1/1	1/1	0/0	0/1	1/1	1/1	intergenic_	MODIFIER	NONE			-1	5S_rRNA-TFB1M				-1
1	18073412		C	T	1090.23		114	1/1	1/1	1/1	0/0	0/1	1/1	1/1	intron_vari	MODIFIER	NONE	c.306+5338G>A		-1	PPP1R14C	protein_coding		ENSSSCT0	1
1	19883824		C	T	1193.87		116	1/1	1/1	1/1	0/0	0/1	0/1	1/1	intergenic_	MODIFIER	NONE			-1	U6-SAMD5				-1
1	22829783		C	T	1504.08		149	1/1	1/1	1/1	0/0	0/1	1/1	1/1	intergenic_	MODIFIER	NONE			-1	EPM2A-ENSSSCG00000004123				-1
1	35841999		A	C	1031.23		127	1/1	0/1	1/1	0/0	0/1	0/1	0/1	intron_vari	MODIFIER	NONE	c.58+9077T>G		-1	ENSSSCG00000022533	protein_coding		ENSSSCT0	1
1	36919425		CA	C	4336.64		222	1/1	1/1	1/1	0/0	0/0	0/1	0/0	intergenic_	MODIFIER	NONE			-1	L3MBTL3-TMEM244				-1
1	37027995		C	T	2424.57		126	1/1	1/1	1/1	0/0	0/1	1/1	1/1	intron_vari	MODIFIER	NONE	c.367+1053G>A		-1	TMEM244	protein_coding		ENSSSCT0	4
1	39516877		C	CA	3080.19		243	1/1	1/1	1/1	0/0	0/0	1/1	0/0	intergenic_	MODIFIER	NONE	n.null_nullinsA		-1	KIAA0408-ENSSSCG00000004216				-1
1	44700398		A	T	1051.44		132	1/1	1/1	1/1	0/0	0/1	1/1	1/1	intergenic_	MODIFIER	NONE			-1	ENSSSCG00000025075-U6				-1
1	56505569		AT	A	2632.13		212	1/1	1/1	1/1	0/0	0/1	0/1	0/0	intron_vari	MODIFIER	NONE	c.2398-1185delT		-1	COL19A1	protein_coding		ENSSSCT0	36
1	65193052		TTTG	T	6720.31		225	1/1	1/1	1/1	0/0	0/1	1/1	0/1	intergenic_	MODIFIER	NONE	n.null_nulldelTTG		-1	BACH2-MAP3K7				-1
1	69551945		G	GT	4114.4		234	1/1	1/1	1/1	0/0	0/0	1/1	0/1	intergenic_	MODIFIER	NONE	n.null_nullinsT		-1	ENSSSCG00000018695-U6				-1
1	74070222		A	C	1754		143	1/1	1/1	1/1	0/0	0/1	1/1	0/1	intergenic_	MODIFIER	NONE			-1	U6-POU3F2				-1
1	78454868		C	T	1234.95		140	1/1	1/1	1/1	0/0	0/1	1/1	0/1	intergenic_	MODIFIER	NONE			-1	GRIK2-HACE1				-1
1	91022831		T	TA	4152.75		224	1/1	1/1	1/1	0/0	0/1	1/1	0/1	intergenic_	MODIFIER	NONE	n.null_nullinsA		-1	HS3ST5-FRK				-1
1	91041942		A	AT	4448.04		255	1/1	1/1	1/1	0/0	0/1	1/1	1/1	intergenic_	MODIFIER	NONE	n.null_nullinsT		-1	HS3ST5-FRK				-1
1	92085699		G	GTGTA	10242.24		263	1/1	1/1	1/1	0/0	0/1	1/1	0/1	intergenic_	MODIFIER	NONE	n.null_nullinsTGTA		-1	TSPYL1-DSE				-1
1	98926610		AT	A	4326.06		214	1/1	1/1	1/1	0/0	0/1	1/1	1/1	intergenic_	MODIFIER	NONE			-1	IRAK1BP1-HTR1B				-1
1	101219056		CA	C	1316.36		185	1/1	1/1	0/1	0/0	0/1	1/1	1/1	intron_vari	MODIFIER	NONE	c.151-2454delT		-1	SENP6	protein_coding		ENSSSCT0	1
1	104051164		C	T	1142.16		137	1/1	1/1	1/1	0/0	0/1	1/1	0/1	intergenic_	MODIFIER	NONE			-1	ENSSSCG00000025743-SETBP1				-1
1	105792014		ATT	A	1996.96		186	1/1	1/1	1/1	0/0	0/1	1/1	0/1	upstream_	MODIFIER	NONE	c.-160_-160delAA		-1	ENSSSCG00000021219	protein_coding		ENSSSCT0	-1
1	105792014		ATT	A	1996.96		186	1/1	1/1	1/1	0/0	0/1	1/1	0/1	intergenic_	MODIFIER	NONE	n.null_nulldelTT		-1	ENSSSCG00000021219-PSTPIP2				-1
1	106239793		T	TG	1629.37		115	1/1	1/1	1/1	0/0	0/1	1/1	0/1	intergenic_	MODIFIER	NONE	n.null_nullinsG		-1	C18orf25-ENSSSCG00000004501				-1
1	106633037		T	C	1196.95		132	1/1	1/1	1/1	0/0	0/1	1/1	1/1	intergenic_	MODIFIER	NONE			-1	ENSSSCG00000004503-SMAD2				-1
1	129393918		G	T	1313.5		157	1/1	1/1	1/1	0/0	0/1	1/1	0/1	intergenic_	MODIFIER	NONE			-1	RSL24D1-UNC13C				-1
1	129403454		A	C	1170.45		153	1/1	1/1	1/1	0/0	0/1	1/1	0/1	intergenic_	MODIFIER	NONE			-1	RSL24D1-UNC13C				-1
1	160968012		T	TA	5489.87		197	1/1	1/1	1/1	0/0	0/0	1/1	1/1	upstream_	MODIFIER	NONE	n.-1_-1insT		-1	ENSSSCG00000025864	miRNA		ENSSSCT0	-1
1	160968012		T	TA	5489.87		197	1/1	1/1	1/1	0/0	0/0	1/1	1/1	intergenic_	MODIFIER	NONE	n.null_nullinsA		-1	ENSSSCG00000025864-ENSSSCG00000004854				-1
1	248790454		A	G	3767.44		208	1/1	1/1	1/1	0/0	1/1	0/1	1/1	intron_vari	MODIFIER	NONE	c.169-20018A>G		-1	FXN	protein_coding		ENSSSCT0	1
1	248790454		A	G	3767.44		208	1/1	1/1	1/1	0/0	1/1	0/1	1/1	intron_vari	MODIFIER	NONE	c.-10+44162A>G		-1	TJP2	protein_coding		ENSSSCT0	2
1	252875103		T	TA	3044.49		212	1/1	1/1	1/1	0/0	0/1	1/1	1/1	intergenic_	MODIFIER	NONE	n.null_nullinsA		-1	ALDH1A1-ANXA1				-1
1	266243714		C	T	6974.13		355	1/1	1/1	1/1	0/0	0/0	1/1	1/1	intron_vari	MODIFIER	NONE	c.-7+18761G>A		-1	ENSSSCG00000005350	protein_coding		ENSSSCT0	2
1	273804829		C	T	1701.83		151	1/1	1/1	1/1	0/0	0/1	1/1	1/1	intergenic_	MODIFIER	NONE			-1	CYLC2-ENSSSCG00000005403				-1
1	287822953		C	A	1043.66		154	1/1	1/1	1/1	0/0	0/1	0/1	1/1	intergenic_	MODIFIER	NONE			-1	ENSSSCG00000005494-PAPPA				-1
1	288065563		G	C	1054.71		150	1/1	1/1	1/1	0/0	0/1	1/1	1/1	intron_vari	MODIFIER	NONE	c.3154-8142G>C		-1	PAPPA	protein_coding		ENSSSCT0	11
1	288065630		G	A	1034.03		146	1/1	1/1	1/1	0/0	0/0	1/1	1/1	intron_vari	MODIFIER	NONE	c.3154-8075G>A		-1	PAPPA	protein_coding		ENSSSCT0	11
1	288527785		T	C	1045.95		151	1/1	1/1	1/1	0/0	0/1	1/1	1/1	intron_vari	MODIFIER	NONE	c.864-3840A>G		-1	ENSSSCG00000005502	protein_coding		ENSSSCT0	8
1	288530920		G	A	1167.11		177	1/1	1/1	1/1	0/0	0/1	1/1	1/1	intron_vari	MODIFIER	NONE	c.864-6975C>T		-1	ENSSSCG00000005502	protein_coding		ENSSSCT0	8
1	288625127		A	G	1030.65		157	1/1	1/1	1/1	0/0	0/1	1/1	1/1	intron_vari	MODIFIER	NONE	c.546-52607T>C		-1	ENSSSCG00000005502	protein_coding		ENSSSCT0	6
1	288625130		A	G	1064.2		159	1/1	1/1	1/1	0/0	0/1	1/1	1/1	intron_vari	MODIFIER	NONE	c.546-52610T>C		-1	ENSSSCG00000005502	protein_coding		ENSSSCT0	6
1	288630610		A	G	1341.85		149	1/1	1/1	1/1	0/0	0/1	1/1	1/1	intron_vari	MODIFIER	NONE	c.546-58090T>C		-1	ENSSSCG00000005502	protein_coding		ENSSSCT0	6
2	8371729		CCA	C	1468.89		210	0/0	0/1	0/1	1/1	1/1	1/1	0/1	intergenic_	MODIFIER	NONE	n.null_nulldelCA		-1	ZBTB3-TTC9C				-1
2	14567383		A	G	1578.91		135	0/0	0/0	0/0	1/1	1/1	./.	1/1	intergenic_	MODIFIER	NONE			-1	ENSSSCG00000024728-ENSSSCG00000014530				-1
2	19127176		CT	C	3059.78		258	0/0	0/0	0/0	1/1	1/1	1/1	0/1	intergenic_	MODIFIER	NONE			-1	TSPAN18-CD82				-1
2	62515493		A	C	1898.26		180	1/1	1/1	1/1	0/0	0/1	0/1	1/1	intergenic_	MODIFIER	NONE			-1	ENSSSCG00000025219-ENSSSCG00000030221				-1
2	63512197		T	C	1262.61		129	1/1	1/1	1/1	0/0	0/1	0/1	1/1	intergenic_	MODIFIER	NONE			-1	ENSSSCG00000028596-ENSSSCG00000013800				-1
2	64090224		C	T	2046.19		193	1/1	1/1	1/1	0/0	0/1	0/1	1/1	intergenic_	MODIFIER	NONE			-1	ENSSSCG00000030169-ENSSSCG00000025816				-1
2	67265991		AT	A	3801.14		236	1/1	1/1	1/1	0/0	0/0	0/1	1/1	3_prime_U	MODIFIER	NONE	c.*243delT		-1	ENSSSCG00000013715	protein_coding		ENSSSCT0	5
2	123359157		C	T	1195.68		157	1/1	1/1	1/1	0/0	0/1	1/1	1/1	intergenic_	MODIFIER	NONE			-1	ENSSSCG00000014212-KCNN2				-1
2	138528306		A	AT	4376.29		216	1/1	1/1	1/1	0/0	0/1	1/1	1/1	intergenic_	MODIFIER	NONE	n.null_nullinsT		-1	CHSY3-HINT1				-1
2	142727197		T	A	1720.93		125	1/1	1/1	1/1	0/0	0/1	1/1	1/1	intergenic_	MODIFIER	NONE			-1	TXNDC15-H2AFY				-1
2	152143693		C	T	1000.43		133	1/1	1/1	1/1	0/0	0/1	1/1	1/1	intergenic_	MODIFIER	NONE			-1	NR3C1-ENSSSCG00000029914				-1
2	152147532		G	A	1384.87		143	1/1	1/1	1/1	0/0	0/1	1/1	1/1	intergenic_	MODIFIER	NONE			-1	NR3C1-ENSSSCG00000029914				-1
2	154590477		A	T	1026.54		147	1/1	1/1	1/1	0/0	0/1	1/1	1/1	intron_vari	MODIFIER	NONE	c.71-73331T>A		-1	PPP2R2B	protein_coding		ENSSSCT0	1
2	155093274		C	T	1334.73		137	1/1	1/1	1/1	0/0	0/1	0/1	1/1	intron_vari	MODIFIER	NONE	c.501+420G>A		-1	ENSSSCG00000027868	protein_coding		ENSSSCT0	5
2	155093277		C	T	1341.38		137	1/1	1/1	1/1	0/0	0/1	0/1	1/1	intron_vari	MODIFIER	NONE	c.501+417G>A		-1	ENSSSCG00000027868	protein_coding		ENSSSCT0	5
2	155093507		G	A	1499.58		154	1/1	1/1	1/1	0/0	0/1	1/1	1/1	intron_vari	MODIFIER	NONE	c.501+187C>T		-1	ENSSSCG00000027868	protein_coding		ENSSSCT0	5
2	156156776		GT	G	3315.31		211	1/1	1/1	1/1	0/0	0/0	1/1	1/1	intergenic_	MODIFIER	NONE			-1	SPINK6-ENSSSCG00000030055				-1
3	25213842		G	A	1519.45		174	0/0	0/1	0/1	1/1	1/1	0/1	1/1	intergenic_	MODIFIER	NONE			-1	ENSSSCG00000007847-ENSSSCG00000007848				-1
3	27990223		G	A	1259.01		155	0/0	0/1	0/1	1/1	1/1	0/1	1/1	intergenic_	MODIFIER	NONE			-1	ENSSSCG00000007872-5S_rRNA				-1
3	29467453		G	A	1111.54		208	0/0	0/1	0/1	1/1	1/1	0/1	1/1	intron_vari	MODIFIER	NONE	c.289+10436G>A		-1	CPPED1	protein_coding		ENSSSCT0	2
3	32430658		C	A	1259.52		132	0/0	0/1	0/1	1/1	1/1	0/1	1/1	intergenic_	MODIFIER	NONE			-1	LITAF-ENSSSCG00000028743				-1
3	33134141		C	T	1105.93		145	0/0	1/1	0/1	1/1	1/1	0/1	1/1	intron_vari	MODIFIER	NONE	c.376+331C>T		-1	ENSSSCG00000007901	protein_coding		ENSSSCT0	5
3	52550229		T	C	1559.23		143	0/0	0/1	0/1	1/1	1/1	1/1	0/1	intergenic_	MODIFIER	NONE			-1	MRPS9-ENSSSCG00000026126				-1
3	52550244		T	C	1518.25		143	0/0	0/1	0/1	1/1	1/1	1/1	0/1	intergenic_	MODIFIER	NONE			-1	MRPS9-ENSSSCG00000026126				-1

Head	Note
#CHROM	Chromosome
POS	Position
ID	Identification
REF	Reference seq (Sscrofa 10.2)
ALT	Alternative sequence
QUAL	Quality
FILTER	
DP	Total depth
EFF[*].EFFE	Genetic element
EFF[*].IMP/	Functional annotation
EFF[*].FUNCLASS	
EFF[*].CODON	
EFF[*].AA	
EFF[*].AA_LEN	
EFF[*].GENE	
EFF[*].BIOTYPE	
EFF[*].CODING	
EFF[*].TRID	
EFF[*].RANK	
./.	Not called
0/0	Homogeneous to REF
0/1	Heterogeneous to REF
1/1	Homogeneous to ALT

3	54769594	A	G	1046.19	168	0/0	0/1	0/1	1/1	1/1	1/1	0/1	intergenic_	MODIFIER	NONE		-1	IL1R1-ENSSSCG00000028331			-1
3	55829690	T	C	1156.22	161	0/0	0/1	0/1	1/1	1/1	1/1	0/1	intron_vari	MODIFIER	NONE	c.*33+6539A>G	-1	RPL31	protein_coding	ENSSSCT0	4
3	55829690	T	C	1156.22	161	0/0	0/1	0/1	1/1	1/1	1/1	0/1	intron_vari	MODIFIER	NONE	c.2549-2990T>C	-1	TBC1D8	protein_coding	ENSSSCT0	15
3	139192913	C	T	1163.73	148	0/0	0/1	0/1	1/1	1/1	0/1	1/1	intergenic_	MODIFIER	NONE		-1	ENSSSCG00000025247-ASGR1			-1
3	6975267	A	G	2397.56	150	1/1	1/1	1/1	0/0	0/1	1/1	1/1	intergenic_	MODIFIER	NONE		-1	ENSSSCG00000024923-ENSSSCG00000023055			-1
3	111300880	T	G	1879.53	194	1/1	1/1	1/1	0/0	0/1	1/1	0/1	intergenic_	MODIFIER	NONE		-1	CRIM1-ENSSSCG00000008506			-1
4	9068490	T	C	1288.22	135	0/0	0/1	0/1	1/1	1/1	0/1	1/1	intron_vari	MODIFIER	NONE	c.169+46T>C	-1	OC90	protein_coding	ENSSSCT0	2
4	9068533	C	G	1109.13	130	0/0	0/1	0/1	1/1	1/1	0/1	1/1	intron_vari	MODIFIER	NONE	c.169+89C>G	-1	OC90	protein_coding	ENSSSCT0	2
4	9084210	T	C	1281.3	160	0/0	0/1	0/1	1/1	1/1	0/1	1/1	intron_vari	MODIFIER	NONE	c.1070+848T>C	-1	OC90	protein_coding	ENSSSCT0	12
4	9086006	T	C	1388.05	212	0/0	0/1	0/1	1/1	1/1	0/1	1/1	intron_vari	MODIFIER	NONE	c.1071-180T>C	-1	OC90	protein_coding	ENSSSCT0	12
4	67994103	TTG	T	1837.94	214	0/0	0/0	0/1	1/1	1/1	0/1	1/1	intron_vari	MODIFIER	NONE	c.814+7317_814+731	-1	STAU2	protein_coding	ENSSSCT0	6
4	74051994	T	C	1357.71	165	0/0	0/1	0/1	1/1	1/1	1/1	1/1	intergenic_	MODIFIER	NONE		-1	ENSSSCG00000006206-ENSSSCG00000023518			-1
4	74052001	T	C	1158.98	164	0/0	0/1	0/1	1/1	1/1	1/1	1/1	intergenic_	MODIFIER	NONE		-1	ENSSSCG00000006206-ENSSSCG00000023518			-1
4	84082675	GA	G	3538.89	265	0/0	0/0	0/1	1/1	1/1	1/1	1/1	intergenic_	MODIFIER	NONE		-1	SOX17-MRPL15			-1
4	86371133	A	G	1238.81	164	0/0	0/1	0/1	1/1	1/1	1/1	1/1	intergenic_	MODIFIER	NONE		-1	PCMTD1-SNAI2			-1
5	9089370	C	CT	2230.04	216	0/0	0/0	0/0	1/1	1/1	1/1	1/1	intron_vari	MODIFIER	NONE	c.3101-81_3101-80ins	-1	MYH9	protein_coding	ENSSSCT0	24
5	27974160	A	G	1202.27	146	0/0	0/1	0/1	1/1	1/1	0/1	1/1	intergenic_	MODIFIER	NONE		-1	U6-ENSSSCG00000000457			-1
5	69132974	GA	G	2087.8	234	0/0	0/0	0/1	1/1	1/1	0/1	1/1	intergenic_	MODIFIER	NONE		-1	PRMT8-ENSSSCG00000000734			-1
5	32719065	G	T	1185.51	122	1/1	1/1	1/1	0/0	0/1	1/1	0/1	upstream_	MODIFIER	NONE	c.-168C>A	-1	ENSSSCG00000000468	protein_coding	ENSSSCT0	-1
5	32719065	G	T	1185.51	122	1/1	1/1	1/1	0/0	0/1	1/1	0/1	intergenic_	MODIFIER	NONE		-1	ENSSSCG00000000468-LEMD3			-1
5	32724194	T	C	1470.07	150	1/1	1/1	1/1	0/0	0/1	1/1	0/1	intergenic_	MODIFIER	NONE		-1	ENSSSCG00000000468-LEMD3			-1
5	33239319	A	G	2126.59	152	1/1	1/1	1/1	0/0	0/1	1/1	0/1	intergenic_	MODIFIER	NONE		-1	ENSSSCG00000024846-HMGA2			-1
5	35475020	G	A	1308.03	169	1/1	1/1	1/1	0/0	0/1	1/1	0/1	intron_vari	MODIFIER	NONE	c.810+403C>T	-1	ENSSSCG00000000483	protein_coding	ENSSSCT0	5
5	48868555	G	C	1205.22	173	1/1	1/1	1/1	0/0	0/1	1/1	0/1	intron_vari	MODIFIER	NONE	c.110-8507G>C	-1	ENSSSCG00000024232	protein_coding	ENSSSCT0	3
5	67490871	G	GCA	3401.17	233	1/1	1/1	1/1	0/0	0/0	1/1	0/0	intergenic_	MODIFIER	NONE	n.null_nullinsCA	-1	ANO2-KV1.5			-1
6	2338761	T	C	1084.1	129	0/0	0/1	0/1	1/1	1/1	1/1	0/1	intergenic_	MODIFIER	NONE		-1	C16orf95-FOXL1			-1
6	3432569	C	G	1130.18	137	0/0	0/1	0/1	1/1	1/1	1/1	0/1	intergenic_	MODIFIER	NONE		-1	MTHFSD-COX4I1			-1
6	17071811	A	C	1049.52	1905	0/0	0/1	0/1	1/1	1/1	1/1	1/1	intergenic_	MODIFIER	NONE		-1	CDH3-RSPRY1			-1
6	29037648	A	G	1211.43	115	0/0	0/1	0/1	1/1	1/1	1/1	1/1	intergenic_	MODIFIER	NONE		-1	ENSSSCG00000002834-TOX3			-1
6	31093904	A	G	1081.53	127	0/0	0/1	0/1	1/1	1/1	1/1	1/1	intergenic_	MODIFIER	NONE		-1	ZNF423-N4BP1			-1
6	59974516	T	C	1514.54	134	0/0	0/1	0/1	1/1	1/1	1/1	1/1	intergenic_	MODIFIER	NONE		-1	ENSSSCG00000003361-C1orf174			-1
6	75048956	T	C	1056.88	139	0/0	0/1	0/1	1/1	1/1	0/1	1/1	intron_vari	MODIFIER	NONE	c.1405-11327T>C	-1	KDM1A	protein_coding	ENSSSCT0	11
6	81281307	G	GT	2735.98	183	0/0	0/1	1/1	1/1	0/1	0/1	1/1	intron_vari	MODIFIER	NONE	c.820-502_820-501ins	-1	SDC3	protein_coding	ENSSSCT0	2
6	20228757	T	C	2811.59	160	1/1	1/1	1/1	0/0	0/1	1/1	1/1	intergenic_	MODIFIER	NONE		-1	U6-ENSSSCG00000028630			-1
6	60449093	C	T	1439.9	166	1/1	1/1	1/1	0/0	0/1	1/1	0/1	intergenic_	MODIFIER	NONE		-1	DFFB-NPHP4			-1
6	60461712	C	T	1183.4	157	1/1	1/1	1/1	0/0	0/1	1/1	0/1	intergenic_	MODIFIER	NONE		-1	DFFB-NPHP4			-1
6	62256068	T	C	1072.37	126	1/1	1/1	1/1	0/0	0/1	1/1	0/1	intron_vari	MODIFIER	NONE	c.1506+1076T>C	-1	ENSSSCG00000022884	protein_coding	ENSSSCT0	7
6	91341433	T	C	1318.18	150	1/1	1/1	1/1	0/0	0/1	1/1	0/1	intergenic_	MODIFIER	NONE		-1	ENSSSCG00000024963-ENSSSCG00000003676			-1
6	91341447	G	A	1149.68	149	1/1	1/1	1/1	0/0	0/1	1/1	0/0	intergenic_	MODIFIER	NONE		-1	ENSSSCG00000024963-ENSSSCG00000003676			-1
6	91341780	A	G	1689.96	156	1/1	1/1	1/1	0/0	0/1	1/1	0/1	intergenic_	MODIFIER	NONE		-1	ENSSSCG00000024963-ENSSSCG00000003676			-1
6	120593989	G	T	2270.57	189	1/1	1/1	1/1	0/0	0/1	1/1	1/1	intergenic_	MODIFIER	NONE		-1	U6-LPHN2			-1
6	121647932	G	A	1204.74	130	1/1	1/1	1/1	0/0	0/1	1/1	1/1	intergenic_	MODIFIER	NONE		-1	U6-LPHN2			-1
6	123941563	A	G	1523.06	157	1/1	1/1	1/1	0/0	0/1	1/1	1/1	intergenic_	MODIFIER	NONE		-1	U1-ELTD1			-1
7	68532987	C	T	1624.32	152	1/1	1/1	1/1	0/0	0/1	1/1	1/1	intergenic_	MODIFIER	NONE		-1	TTF1-MBIP			-1
7	68665023	T	C	1039.42	140	1/1	1/1	1/1	0/0	0/1	1/1	1/1	intergenic_	MODIFIER	NONE		-1	TTF1-MBIP			-1
7	70715963	A	G	1332.83	134	1/1	1/1	1/1	0/0	0/1	1/1	1/1	intergenic_	MODIFIER	NONE		-1	EGLN3-NPAS3			-1
7	115847997	C	T	1149.98	148	1/1	1/1	1/1	0/0	0/1	1/1	0/1	intergenic_	MODIFIER	NONE		-1	FLRT2-ENSSSCG00000018967			-1
7	115853632	G	A	1244.88	117	1/1	1/1	1/1	0/0	0/1	1/1	0/1	intergenic_	MODIFIER	NONE		-1	FLRT2-ENSSSCG00000018967			-1
7	118868555	C	A	1971.37	169	1/1	1/1	1/1	0/0	0/1	1/1	1/1	intron_vari	MODIFIER	NONE	c.1991-637G>T	-1	ENSSSCG00000002436	protein_coding	ENSSSCT0	21
7	118868622	G	A	2226.83	174	1/1	1/1	1/1	0/0	0/1	1/1	1/1	intron_vari	MODIFIER	NONE	c.1991-704C>T	-1	ENSSSCG00000002436	protein_coding	ENSSSCT0	21
8	5768168	AG	A	1536.66	196	0/0	0/1	0/1	1/1	1/1	0/1	1/1	intergenic_	MODIFIER	NONE		-1	TMEM128-LYAR			-1
8	21945383	A	G	1116.71	139	0/0	0/1	1/1	1/1	1/1	0/1	1/1	intergenic_	MODIFIER	NONE		-1	7SK-ENSSSCG00000008763			-1
8	21945384	T	C	1257.37	140	0/0	0/1	1/1	1/1	1/1	1/1	1/1	intergenic_	MODIFIER	NONE		-1	7SK-ENSSSCG00000008763			-1
8	23009229	CG	C	1080.68	241	0/0	0/0	0/0	1/1	1/1	1/1	1/1	intergenic_	MODIFIER	NONE		-1	ENSSSCG00000008763-PCDH7			-1
8	26874123	A	C	1342.97	136	0/0	0/1	0/1	1/1	1/1	1/1	1/1	intergenic_	MODIFIER	NONE		-1	PCDH7-ENSSSCG00000008767			-1
8	32890209	C	CT	1085.9	213	0/0	0/1	0/1	1/1	1/1	0/1	0/1	intron_vari	MODIFIER	NONE	c.406+1180_406+118	-1	N4BP2	protein_coding	ENSSSCT0	2
8	33762665	GT	G	1159.43	216	0/0	1/1	1/1	1/1	1/1	1/1	0/1	intergenic_	MODIFIER	NONE		-1	NSUN7-U6			-1
8	39555553	T	TTA	4723.47	227	0/0	0/0	0/0	1/1	1/1	1/1	1/1	intron_vari	MODIFIER	NONE	c.2411+1756_2411+1	-1	CORIN	protein_coding	ENSSSCT0	16
8	39652185	GAGA	G	1633.46	200	0/0	0/0	0/0	1/1	1/1	0/1	0/1	intron_vari	MODIFIER	NONE	c.1012+13959_1012+	-1	CORIN	protein_coding	ENSSSCT0	5
8	45200463	CT	C	2816.01	228	0/0	0/1	0/0	1/1	1/1	1/1	1/1	intergenic_	MODIFIER	NONE		-1	ENSSSCG00000024269-CPE			-1
8	2105756	C	T	1402.74	152	1/1	1/1	1/1	0/0	0/1	1/1	1/1	intergenic_	MODIFIER	NONE		-1	ADRA2C-ENSSSCG00000028524			-1
8	6822147	T	C	1048.26	126	1/1	1/1	1/1	0/0	0/1	1/1	0/1	intergenic_	MODIFIER	NONE		-1	SNORA11-U6			-1
8	7605447	T	TA	2583.62	250	1/1	0/1	1/1	0/0	0/1	1/1	0/1	intergenic_	MODIFIER	NONE	n.null_nullinsA	-1	U6-ENSSSCG00000008734			-1
8	9287694	T	TAA	6960.88	229	1/1	1/1	1/1	0/0	0/1	1/1	0/1	intergenic_	MODIFIER	NONE	n.null_nullinsAA	-1	BOD1L1-CPEB2			-1
8	9437147	C	T	1028.4	138	1/1	1/1	1/1	0/0	0/1	1/1	0/1	intergenic_	MODIFIER	NONE		-1	BOD1L1-CPEB2			-1
8	75790028	G	A	3313.66	386	1/1	1/1	1/1	0/0	0/0	0/1	0/0	intergenic_	MODIFIER	NONE		-1	SDAD1-CXCL9			-1
8	137455360	TAC	T	2891.31	193	1/1	1/1	0/1	0/0	0/1	0/1	1/1	intergenic_	MODIFIER	NONE	n.null_nulldelAC	-1	ENSSSCG00000009200-ENSSSCG00000029197			-1
8	138078088	AT	A	2739.72	169	1/1	1/1	1/1	0/0	0/0	0/1	1/1	intron_vari	MODIFIER	NONE	c.1510-27278delA	-1	ENSSSCG00000022986	protein_coding	ENSSSCT0	2
8	138125853	A	ATG	4606.86	212	1/1	1/1	1/1	0/0	0/1	0/1	1/1	intron_vari	MODIFIER	NONE	c.1509+897_1509+89	-1	ENSSSCG00000022986	protein_coding	ENSSSCT0	2
9	4683739	A	G	1065.02	152	0/0	1/1	0/1	1/1	1/1	1/1	1/1	intergenic_	MODIFIER	NONE		-1	ENSSSCG00000014673-TRIM6			-1
9	55718387	A	G	1027.08	160	0/0	0/1	0/1	1/1	1/1	1/1	0/1	intergenic_	MODIFIER	NONE		-1	ENSSSCG00000015140-ENSSSCG00000015144			-1
9	55737157	G	T	1022.89	124	0/0	0/1	0/1	1/1	1/1	1/1	1/1	intergenic_	MODIFIER	NONE		-1	ENSSSCG00000015140-ENSSSCG00000015144			-1
9	56254834	T	A	2035.29	138	0/0	0/1	0/1	1/1	1/1	1/1	1/1	intergenic_	MODIFIER	NONE		-1	ENSSSCG00000015162-ENSSSCG00000025623			-1
9	58075484	G	A	1752.15	167	0/0	0/1	0/1	1/1	1/1	1/1	0/1	intergenic_	MODIFIER	NONE		-1	TMEM218-ENSSSCG00000015211			-1
9	90821723	G	GA	1029.34	224	0/0	0/1	0/1	1/1	0/1	0/1	1/1	intergenic_	MODIFIER	NONE	n.null_nullinsA	-1	ARL4A-ETV1			-1
9	100140721	T	TGG	1325.41	223	0/0	0/0	0/1	1/1	1/1	0/1	1/1	intron_vari	MODIFIER	NONE	c.11403+301_11403+	-1	DNAH11	protein_coding	ENSSSCT0	71

9	100155385	G	A	1425.94	173	0/0	0/1	0/1	1/1	1/1	0/1	1/1	intron_vari	MODIFIER	NONE	c.12103-726G>A	-1	DNAH11	protein_coding	ENSSSCT0	75
9	100155403	A	C	1166.23	168	0/0	0/1	0/1	1/1	1/1	0/1	1/1	intron_vari	MODIFIER	NONE	c.12103-708A>C	-1	DNAH11	protein_coding	ENSSSCT0	75
9	101021728	A	G	1067.48	130	0/0	0/1	0/1	1/1	1/1	0/1	1/1	upstream_	MODIFIER	NONE	c.-64A>G	-1	IL6	protein_coding	ENSSSCT0	-1
9	101021728	A	G	1067.48	130	0/0	0/1	0/1	1/1	1/1	0/1	1/1	intergenic_	MODIFIER	NONE		-1	TOMM7-IL6			-1
9	101129802	CG	C	1229.56	254	0/0	0/1	0/0	1/1	1/1	0/0	0/1	intergenic_	MODIFIER	NONE		-1	IL6-FAM126A			-1
9	100003131	CTG	C	2495.47	210	1/1	1/1	1/1	0/0	0/1	1/1	1/1	intron_vari	MODIFIER	NONE	c.6902+405_6902+40	-1	DNAH11	protein_coding	ENSSSCT0	42
9	106793277	G	A	1032.06	144	1/1	1/1	1/1	0/0	0/1	1/1	1/1	intergenic_	MODIFIER	NONE		-1	ENSSSCG00000022472-SEMA3E			-1
10	29938018	A	C	3839.15	362	0/0	0/1	0/1	1/1	1/1	0/1	1/1	intergenic_	MODIFIER	NONE		-1	ENSSSCG00000010935-ENSSSCG00000010936			-1
10	45881163	T	C	3118.53	370	0/0	0/0	0/0	1/1	1/1	0/1	0/1	intron_vari	MODIFIER	NONE	c.6324+407A>G	-1	SVIL	protein_coding	ENSSSCT0	35
10	64031898	A	G	1811.04	138	0/0	0/1	0/1	1/1	1/1	1/1	1/1	intergenic_	MODIFIER	NONE		-1	GJD4-CCDC3			-1
10	9069068	G	A	1559.53	99	1/1	1/1	1/1	0/0	0/1	1/1	1/1	intergenic_	MODIFIER	NONE		-1	ESRRG-U6atac			-1
10	9090595	T	C	1283.95	134	1/1	1/1	1/1	0/0	0/1	1/1	1/1	intergenic_	MODIFIER	NONE		-1	ESRRG-U6atac			-1
10	26749124	C	T	1307.33	134	1/1	1/1	1/1	0/0	0/1	1/1	1/1	intergenic_	MODIFIER	NONE		-1	ssc-mir-181a-1-NR5A2			-1
10	26800355	T	G	1700.76	141	1/1	1/1	1/1	0/0	0/1	1/1	0/1	intergenic_	MODIFIER	NONE		-1	ssc-mir-181a-1-NR5A2			-1
10	26981224	GT	G	3674.52	206	1/1	1/1	1/1	0/0	0/1	1/1	1/1	intergenic_	MODIFIER	NONE		-1	ssc-mir-181a-1-NR5A2			-1
10	32797716	A	G	1476.19	118	1/1	1/1	1/1	0/0	0/1	1/1	0/1	intergenic_	MODIFIER	NONE		-1	DAPK1-ZCCHC6			-1
10	32908624	C	CT	1235.55	206	1/1	0/1	1/1	0/0	0/1	1/1	0/0	intergenic_	MODIFIER	NONE	n.null_nullinsT	-1	DAPK1-ZCCHC6			-1
10	38421472	T	C	1245.22	126	1/1	1/1	1/1	0/0	0/1	1/1	0/1	intergenic_	MODIFIER	NONE		-1	ENSSSCG00000028539-SNORA31			-1
10	38853301	TAA	T	3562.76	182	1/1	1/1	1/1	0/0	0/0	1/1	0/0	intergenic_	MODIFIER	NONE	n.null_nulldelAA	-1	SNORA31-U6			-1
10	54346537	A	G	1569.22	186	1/1	1/1	1/1	0/0	0/1	0/1	0/1	intron_vari	MODIFIER	NONE	c.463-540A>G	-1	ENSSSCG00000011062	protein_coding	ENSSSCT0	3
10	54488825	A	G	1405.58	131	1/1	1/1	1/1	0/0	0/1	0/1	0/1	intron_vari	MODIFIER	NONE	c.137-1393T>C	-1	ENSSSCG00000024127	protein_coding	ENSSSCT0	1
10	60017208	T	C	1606.47	171	1/1	1/1	1/1	0/0	0/1	1/1	0/1	intergenic_	MODIFIER	NONE		-1	ENSSSCG00000011090-ENSSSCG00000029563			-1
10	69069289	G	A	2216.22	185	1/1	1/1	1/1	0/0	0/1	0/1	0/1	intergenic_	MODIFIER	NONE		-1	U6-ENSSSCG00000029177			-1
10	70247443	T	C	2151.6	133	1/1	1/1	1/1	0/0	0/1	0/1	1/1	intergenic_	MODIFIER	NONE		-1	U6-ENSSSCG00000018589			-1
11	26161070	A	G	1055.38	162	0/0	0/1	0/0	1/1	1/1	0/1	0/1	intergenic_	MODIFIER	NONE		-1	ENSSSCG00000024707-RGCC			-1
11	71093622	A	T	1221.43	211	0/0	0/1	0/1	1/1	1/1	0/1	1/1	intron_vari	MODIFIER	NONE	c.273+592A>T	-1	ENSSSCG00000022549	protein_coding	ENSSSCT0	2
11	80970713	G	A	1544.19	140	0/0	0/1	0/1	1/1	1/1	0/1	1/1	intergenic_	MODIFIER	NONE		-1	ENSSSCG00000026317-EFNB2			-1
12	38609885	AT	A	1116.44	197	0/0	0/0	0/1	1/1	0/1	0/1	0/1	intergenic_	MODIFIER	NONE		-1	ssc-mir-378-2-BCAS3			-1
12	39592974	T	TC	1977.15	262	1/1	0/1	1/1	0/0	0/0	0/1	0/1	upstream_	MODIFIER	NONE	c.-87_-87insG	-1	ZNHIT3	protein_coding	ENSSSCT0	-1
12	39592974	T	TC	1977.15	262	1/1	0/1	1/1	0/0	0/0	0/1	0/1	intergenic_	MODIFIER	NONE	n.null_nullinsC	-1	ZNHIT3-CA4			-1
13	403773	C	CA	3243.74	202	0/0	0/0	0/1	1/1	1/1	1/1	1/1	intron_vari	MODIFIER	NONE	c.180+44121_180+44	-1	ENSSSCG00000023343	protein_coding	ENSSSCT0	1
13	134095135	G	A	1307.6	143	0/0	0/1	0/1	1/1	1/1	1/1	0/1	intergenic_	MODIFIER	NONE		-1	RFC4-MASP1			-1
13	187330423	G	GA	1764.93	238	0/0	0/1	0/1	1/1	1/1	1/1	1/1	intergenic_	MODIFIER	NONE	n.null_nullinsA	-1	ROBO1-ENSSSCG00000012002			-1
13	215237009	C	CGGGAGA	10414.89	225	0/0	0/1	0/1	1/1	1/1	1/1	0/1	intergenic_	MODIFIER	NONE	n.null_nullinsGGGAG/	-1	ENSSSCG00000012078-ENSSSCG00000012082			-1
13	7538116	A	AAAG	7576.99	255	1/1	1/1	1/1	0/0	0/1	0/1	0/1	intergenic_	MODIFIER	NONE	n.null_nullinsAAG	-1	KCNH8-EFHB			-1
13	16751373	T	C	1231.24	178	1/1	0/1	1/1	0/0	1/1	1/1	0/1	intergenic_	MODIFIER	NONE		-1	AZI2-ENSSSCG00000024265			-1
13	88109695	A	G	1269.86	134	1/1	1/1	1/1	0/0	0/1	1/1	1/1	intron_vari	MODIFIER	NONE	c.185+2049T>C	-1	ENSSSCG00000030661	protein_coding	ENSSSCT0	1
13	88290484	A	C	2550.09	152	1/1	1/1	1/1	0/0	0/1	1/1	1/1	intergenic_	MODIFIER	NONE		-1	U6-ENSSSCG00000026073			-1
13	89712326	T	A	3012.81	154	1/1	1/1	1/1	0/0	0/1	1/1	1/1	intron_vari	MODIFIER	NONE	c.694+530T>A	-1	SPSB4	protein_coding	ENSSSCT0	1
13	161985892	A	G	2679.46	153	1/1	0/1	0/1	0/0	0/1	0/1	1/1	intergenic_	MODIFIER	NONE		-1	ENSSSCG00000011945-CBLB			-1
14	62454044	GT	G	2509.49	237	0/0	0/1	0/1	1/1	1/1	1/1	0/1	intron_vari	MODIFIER	NONE	c.3350+733delA	-1	SIPA1L2	protein_coding	ENSSSCT0	11
14	2348961	A	G	1976.25	116	1/1	1/1	1/1	0/0	0/1	1/1	1/1	intergenic_	MODIFIER	NONE		-1	U6-U6			-1
14	2387796	G	A	1454.6	113	1/1	1/1	1/1	0/0	0/1	1/1	1/1	intergenic_	MODIFIER	NONE		-1	U6-ENSSSCG00000027841			-1
14	7732433	T	TG	1084.25	159	1/1	1/1	1/1	0/0	0/1	0/1	0/1	intergenic_	MODIFIER	NONE	n.null_nullinsG	-1	PEBP4-ENSSSCG00000027496			-1
14	9485038	CA	C	3348.82	212	1/1	1/1	1/1	0/0	0/0	0/1	1/1	intergenic_	MODIFIER	NONE		-1	ADAM28-ADAMDEC1			-1
14	72609770	A	AT	1430.63	261	1/1	1/1	1/1	0/0	0/0	1/1	0/0	intergenic_	MODIFIER	NONE	n.null_nullinsT	-1	ENSSSCG00000028393-CTNNA3			-1
14	73715373	C	CT	1754.43	257	1/1	1/1	1/1	0/0	0/1	1/1	0/1	intergenic_	MODIFIER	NONE	n.null_nullinsT	-1	ENSSSCG00000028393-CTNNA3			-1
14	87837974	GT	G	2734.42	220	1/1	1/1	1/1	0/0	0/1	1/1	0/1	intergenic_	MODIFIER	NONE		-1	ENSSSCG00000010328-ENSSSCG00000010329			-1
14	134704523	T	TA	1830.69	235	1/1	0/1	1/1	0/0	0/1	1/1	1/1	intron_vari	MODIFIER	NONE	c.93+37916_93+3791	-1	TCF7L2	protein_coding	ENSSSCT0	4
14	136144494	T	TA	1028.85	214	1/1	1/1	1/1	0/0	0/1	0/1	1/1	intergenic_	MODIFIER	NONE	n.null_nullinsA	-1	ABLIM1-FAM160B1			-1
15	19740207	C	A	1451.84	147	0/0	0/1	0/1	1/1	1/1	1/1	1/1	intergenic_	MODIFIER	NONE		-1	ENSSSCG00000029118-ENSSSCG00000015700			-1
15	24436358	T	C	1285.4	173	0/0	0/1	0/1	1/1	1/1	1/1	1/1	intergenic_	MODIFIER	NONE		-1	U6-ENSSSCG00000026504			-1
15	29972309	G	A	1001.36	145	0/0	0/1	0/1	1/1	1/1	1/1	0/1	intergenic_	MODIFIER	NONE		-1	GYPC-ENSSSCG00000023800			-1
15	34676931	C	T	1022.27	153	0/0	0/1	0/1	1/1	1/1	1/1	0/1	upstream_	MODIFIER	NONE	c.-76C>T	-1	ENSSSCG00000015730	protein_coding	ENSSSCT0	-1
15	34676931	C	T	1022.27	153	0/0	0/1	0/1	1/1	1/1	1/1	0/1	intergenic_	MODIFIER	NONE		-1	TSN-ENSSSCG00000015730			-1
15	38157850	T	TA	1519.66	225	0/0	0/1	0/1	1/1	1/1	1/1	1/1	intergenic_	MODIFIER	NONE	n.null_nullinsA	-1	ENSSSCG00000015747-ENSSSCG00000029846			-1
15	40325994	A	C	1181.59	141	0/0	0/1	0/1	1/1	1/1	1/1	0/1	intergenic_	MODIFIER	NONE		-1	ENSSSCG00000015748-U6			-1
15	65506863	G	A	1070.23	133	0/0	0/1	0/1	1/1	1/1	1/1	1/1	intergenic_	MODIFIER	NONE		-1	SNORA19-HS6ST1			-1
15	65506911	C	T	1551.78	156	0/0	0/1	0/1	1/1	1/1	1/1	1/1	intergenic_	MODIFIER	NONE		-1	SNORA19-HS6ST1			-1
15	108891274	A	C	1425.75	160	0/0	0/1	0/1	1/1	1/1	1/1	1/1	intergenic_	MODIFIER	NONE		-1	TMEFF2-STK17B			-1
15	128115479	G	A	1458.16	152	0/0	0/1	0/1	1/1	1/1	0/1	0/1	intergenic_	MODIFIER	NONE		-1	SPAG16-ENSSSCG00000029683			-1
15	128115480	T	C	1488.16	152	0/0	0/1	0/1	1/1	1/1	0/1	0/1	intergenic_	MODIFIER	NONE		-1	SPAG16-ENSSSCG00000029683			-1
15	135525297	G	T	1526.59	126	0/0	0/1	0/1	1/1	1/1	1/1	1/1	intergenic_	MODIFIER	NONE		-1	U6-SNORA31			-1
15	143768754	G	C	1042.17	147	1/1	1/1	1/1	0/0	0/1	1/1	1/1	intergenic_	MODIFIER	NONE		-1	U6-DNER			-1
15	151200121	C	T	2555.34	143	1/1	0/1	1/1	0/0	0/0	0/1	0/1	intergenic_	MODIFIER	NONE		-1	ACKR3-COPS8			-1
15	154317267	G	T	1570.02	306	1/1	1/1	1/1	0/0	0/1	0/1	0/1	intergenic_	MODIFIER	NONE		-1	ENSSSCG00000030711-ENSSSCG00000020714			-1
16	8032570	GA	G	2914.9	230	0/0	0/1	0/0	1/1	1/1	1/1	0/1	intergenic_	MODIFIER	NONE		-1	7SK-CDH18			-1
16	20790534	A	G	1658.17	148	0/0	0/1	0/1	1/1	1/1	1/1	0/1	intergenic_	MODIFIER	NONE		-1	AMACR-C1QTNF3			-1
16	59844155	G	C	1032.56	119	0/0	0/1	0/0	1/1	0/1	0/1	1/1	intergenic_	MODIFIER	NONE		-1	SLIT3-ENSSSCG00000026810			-1
16	2057205	A	G	1136.5	105	1/1	1/1	1/1	0/0	0/1	1/1	0/1	intergenic_	MODIFIER	NONE		-1	U6-ENSSSCG00000029792			-1
16	2950544	G	A	1513.69	165	1/1	1/1	1/1	0/0	0/1	1/1	0/1	intergenic_	MODIFIER	NONE		-1	U6-ENSSSCG00000029792			-1
16	24919650	G	C	4563.38	343	1/1	1/1	1/1	0/0	0/0	0/1	0/1	intergenic_	MODIFIER	NONE		-1	EGFLAM-LIFR			-1
16	51406679	T	C	1302.65	110	1/1	1/1	1/1	0/0	0/1	0/1	1/1	intron_vari	MODIFIER	NONE	c.1186-1222A>G	-1	MARVELD2	protein_coding	ENSSSCT0	4
16	51406680	G	A	1242.66	108	1/1	1/1	1/1	0/0	0/1	0/1	1/1	intron_vari	MODIFIER	NONE	c.1186-1223C>T	-1	MARVELD2	protein_coding	ENSSSCT0	4
16	51406682	G	A	1272.66	108	1/1	1/1	1/1	0/0	0/1	0/1	1/1	intron_vari	MODIFIER	NONE	c.1186-1225C>T	-1	MARVELD2	protein_coding	ENSSSCT0	4
16	51406719	A	C	1156.74	113	1/1	1/1	1/1	0/0	0/1	0/1	1/1	intron_vari	MODIFIER	NONE	c.1186-1262T>G	-1	MARVELD2	protein_coding	ENSSSCT0	4

16	51505992	C	T	1445.73	139	1/1	1/1	1/1	0/0	1/1	0/1	1/1	3_prime_U	MODIFIER	NONE	c.*563C>T	-1	ENSSSCG00000027784	protein_coding	ENSSSCT0 3
17	40342407	C	T	1218.62	126	0/0	0/1	0/1	1/1	1/1	1/1	1/1	upstream_	MODIFIER	NONE	c.-1C>T	-1	ENSSSCG00000022547	protein_coding	ENSSSCT0 -1
17	40342407	C	T	1218.62	126	0/0	0/1	0/1	1/1	1/1	1/1	1/1	intron_vari	MODIFIER	NONE	c.-172+1139C>T	-1	DUSP15	protein_coding	ENSSSCT0 3
17	40342423	A	T	1394.48	134	0/0	0/1	0/1	1/1	1/1	1/1	1/1	upstream_	MODIFIER	NONE	c.-1A>T	-1	ENSSSCG00000022547	protein_coding	ENSSSCT0 -1
17	40342423	A	T	1394.48	134	0/0	0/1	0/1	1/1	1/1	1/1	1/1	intron_vari	MODIFIER	NONE	c.-172+1155A>T	-1	DUSP15	protein_coding	ENSSSCT0 3
17	40342441	T	G	1631.21	147	0/0	0/1	0/1	1/1	1/1	1/1	1/1	upstream_	MODIFIER	NONE	c.-1T>G	-1	ENSSSCG00000022547	protein_coding	ENSSSCT0 -1
17	40342441	T	G	1631.21	147	0/0	0/1	0/1	1/1	1/1	1/1	1/1	intron_vari	MODIFIER	NONE	c.-172+1173T>G	-1	DUSP15	protein_coding	ENSSSCT0 3
17	43895202	T	A	1201.82	145	0/0	0/1	0/1	1/1	1/1	1/1	1/1	intron_vari	MODIFIER	NONE	c.24+870A>T	-1	ENSSSCG00000024732	protein_coding	ENSSSCT0 1
17	645031	G	T	1223.56	144	1/1	1/1	1/1	0/0	0/1	1/1	1/1	intergenic_	MODIFIER	NONE		-1	ENSSSCG00000020727-LONRF1		-1
17	645313	G	C	1004.64	139	1/1	1/1	1/1	0/0	0/1	1/1	1/1	intergenic_	MODIFIER	NONE		-1	ENSSSCG00000020727-LONRF1		-1
17	645399	C	A	1151.83	147	1/1	1/1	1/1	0/0	0/1	1/1	1/1	intergenic_	MODIFIER	NONE		-1	ENSSSCG00000020727-LONRF1		-1
17	645416	C	T	1062.03	148	1/1	1/1	1/1	0/0	0/1	1/1	1/1	intergenic_	MODIFIER	NONE		-1	ENSSSCG00000020727-LONRF1		-1
17	645425	A	C	1075.99	144	1/1	1/1	1/1	0/0	0/1	1/1	1/1	intergenic_	MODIFIER	NONE		-1	ENSSSCG00000020727-LONRF1		-1
17	1049983	G	A	2212.35	193	1/1	1/1	1/1	0/0	0/1	1/1	1/1	intergenic_	MODIFIER	NONE		-1	KIAA1456-ENSSSCG00000006970		-1
17	3976350	G	C	1156.5	136	1/1	1/1	1/1	0/0	1/1	1/1	1/1	intergenic_	MODIFIER	NONE		-1	SNORA40-ENSSSCG00000023088		-1
17	4784431	A	G	1199.96	161	1/1	1/1	1/1	0/0	0/1	1/1	1/1	intergenic_	MODIFIER	NONE		-1	MSR1-FGF20		-1
17	4784436	T	C	1239.39	162	1/1	1/1	1/1	0/0	0/1	1/1	1/1	intergenic_	MODIFIER	NONE		-1	MSR1-FGF20		-1
17	4985029	C	T	2174.58	140	1/1	1/1	1/1	0/0	0/1	1/1	1/1	intergenic_	MODIFIER	NONE		-1	MSR1-FGF20		-1
17	5008940	C	T	1296.8	109	1/1	1/1	1/1	0/0	0/1	1/1	1/1	intergenic_	MODIFIER	NONE		-1	MSR1-FGF20		-1
17	13008209	G	A	1238	124	1/1	1/1	1/1	0/0	0/1	1/1	1/1	intron_vari	MODIFIER	NONE	c.1562+1919G>A	-1	ENSSSCG00000026968	protein_coding	ENSSSCT0 21
17	13008215	T	C	1266	123	1/1	1/1	1/1	0/0	0/1	1/1	1/1	intron_vari	MODIFIER	NONE	c.1562+1925T>C	-1	ENSSSCG00000026968	protein_coding	ENSSSCT0 21
17	13008216	T	C	1298	124	1/1	1/1	1/1	0/0	0/1	1/1	1/1	intron_vari	MODIFIER	NONE	c.1562+1926T>C	-1	ENSSSCG00000026968	protein_coding	ENSSSCT0 21
18	40749240	G	GA	1047.25	198	0/0	0/1	0/1	1/1	1/1	1/1	0/1	intron_vari	MODIFIER	NONE	c.-141+11760_-141+1	-1	ELMO1	protein_coding	ENSSSCT0 14
18	46377954	A	G	1019.53	97	0/0	0/1	0/1	1/1	1/1	1/1	1/1	intergenic_	MODIFIER	NONE		-1	PAC1-GHRHR		-1
18	1030043	A	G	1267.17	128	1/1	1/1	1/1	0/0	0/1	1/1	0/1	intergenic_	MODIFIER	NONE		-1	ENSSSCG00000016405-ENSSSCG00000026661		-1
18	1030044	A	G	1218.21	128	1/1	1/1	1/1	0/0	0/1	1/1	0/1	intergenic_	MODIFIER	NONE		-1	ENSSSCG00000016405-ENSSSCG00000026661		-1
18	1030052	C	T	1342.16	144	1/1	1/1	1/1	0/0	0/1	1/1	0/1	intergenic_	MODIFIER	NONE		-1	ENSSSCG00000016405-ENSSSCG00000026661		-1
18	2145460	G	A	1633.53	126	1/1	1/1	1/1	0/0	0/1	1/1	1/1	intergenic_	MODIFIER	NONE		-1	ENSSSCG00000016412-SHH		-1
18	2594667	A	G	1458.24	142	1/1	./.	1/1	0/0	0/1	1/1	0/1	intergenic_	MODIFIER	NONE		-1	ENSSSCG00000016412-SHH		-1
18	2594681	G	A	1541.87	150	1/1	./.	1/1	0/0	0/1	1/1	0/1	intergenic_	MODIFIER	NONE		-1	ENSSSCG00000016412-SHH		-1
18	4522996	T	C	1404.99	170	1/1	1/1	1/1	0/0	0/1	1/1	0/1	intergenic_	MODIFIER	NONE		-1	ENSSSCG00000016424-XRCC2		-1
18	4662640	C	G	1931.1	177	1/1	1/1	1/1	0/0	0/1	1/1	1/1	intergenic_	MODIFIER	NONE		-1	ENSSSCG00000016424-XRCC2		-1
18	4975005	G	A	1327	108	1/1	1/1	1/1	0/0	0/1	1/1	0/1	intron_vari	MODIFIER	NONE	c.39+37593G>A	-1	XRCC2	protein_coding	ENSSSCT0 1
18	4975005	G	A	1327	108	1/1	1/1	1/1	0/0	0/1	1/1	0/1	intron_vari	MODIFIER	NONE	c.304-3778G>A	-1	ENSSSCG00000016426	protein_coding	ENSSSCT0 3
18	17502037	AG	A	1799.14	241	1/1	1/1	1/1	0/0	0/0	1/1	1/1	intergenic_	MODIFIER	NONE		-1	CHCHD3-PODXL		-1
18	44713067	A	G	1004.98	154	1/1	1/1	1/1	0/0	0/1	0/1	0/1	intergenic_	MODIFIER	NONE		-1	ENSSSCG00000021095-LSM5		-1
18	45989448	A	G	1109.04	165	1/1	1/1	1/1	0/0	0/1	0/1	1/1	intergenic_	MODIFIER	NONE		-1	PPP1R17-PAC1		-1
X	84273742	C	T	2050.38	492	1/1	0/1	0/1	0/0	0/1	0/1	0/1	intergenic_	MODIFIER	NONE		-1	CH242-184K14.2-CH242-132M16.1		-1
