# Supplementary material for: No excessive mutations in transcription activator-like effector nuclease-mediated α-1,3-galactosyltransferase knockout Yucatan miniature pigs
Source: Asian-Australas J Anim Sci. 2019 Aug 23;33(2):360–72. doi: 10.5713/ajas.19.0480 (PMC6946973; doi:10.5713/ajas.19.0480)
Supplement: Supplementary file 4 [file ajas-19-0480-suppl4.pdf]

Supplementary Table 4. Variant calls of wild type strain control WT1

#CHROM	POS	ID	REF	ALT	QUAL	FILTER	DP	Donor	CJ1	CB1	CB3	WT1	WT2	WT3	EFF[*].EFFECT	EFF[*].IMPACT	EFF[*].FUNCLASS	EFF[*].CODON	EFF[*].AA	EFF[*].AA_LEN	EFF[*].GENE	EFF[*].BIOTYPE	EFF[*].CODING	EFF[*].TRID	EFF[*].RANK
1	1.82E+08		T	C	4023.92		210	0/0	0/1	0/1	1/1	1/1	1/1	1/1	intergenic_	MODIFIER	NONE			-1	U2-DIS3L				-1
1	1.9E+08		A	G	1167.65		122	0/0	1/1	0/1	1/1	1/1	1/1	1/1	intergenic_	MODIFIER	NONE			-1	5S_rRNA-U6				-1
1	12865248		G	A	1443.27		138	1/1	1/1	1/1	1/1	0/0	1/1	1/1	intergenic_	MODIFIER	NONE			-1	5S_rRNA-TFB1M				-1
1	12865258		T	C	1485.55		143	1/1	1/1	1/1	0/1	0/0	1/1	1/1	intergenic_	MODIFIER	NONE			-1	5S_rRNA-TFB1M				-1
1	12865273		G	A	1686.78		146	1/1	1/1	1/1	0/1	0/0	1/1	1/1	intergenic_	MODIFIER	NONE			-1	5S_rRNA-TFB1M				-1
1	13855875		T	A	1428.83		136	1/1	1/1	1/1	0/1	0/0	1/1	1/1	intergenic_	MODIFIER	NONE			-1	TIAM2-ENSSSCG00000026474				-1
1	22077624		T	TTTTC	3371.45		204	1/1	0/1	0/1	0/1	0/0	0/1	0/1	intron_vari	MODIFIER	NONE	c.293-8837_293-8836		-1	EPM2A	protein_coding		ENSSSCT0	1
1	25325718		C	A	1375.51		171	1/1	1/1	1/1	0/1	0/0	1/1	0/1	intron_vari	MODIFIER	NONE	c.82+26877C>A		-1	GPR126	protein_coding		ENSSSCT0	2
1	26002169		T	C	1156.6		112	1/1	1/1	1/1	0/1	0/0	0/1	0/1	intergenic_	MODIFIER	NONE			-1	NMBR-ENSSSCG00000019021				-1
1	32321590		AGAG	A	4273.41		211	1/1	1/1	1/1	0/1	0/0	1/1	0/1	intergenic_	MODIFIER	NONE	n.null_nulldelGAG		-1	MYB-HBS1L				-1
1	36919425		CA	C	4336.64		222	1/1	1/1	1/1	0/0	0/0	0/1	0/0	intergenic_	MODIFIER	NONE			-1	L3MBTL3-TMEM244				-1
1	39516877		C	CA	3080.19		243	1/1	1/1	1/1	0/0	0/0	1/1	0/0	intergenic_	MODIFIER	NONE	n.null_nullinsA		-1	KIAA0408-ENSSSCG00000004216				-1
1	57679142		A	G	1176.21		146	1/1	1/1	1/1	0/1	0/0	0/1	0/1	intergenic_	MODIFIER	NONE			-1	OGFRL1-ssc-mir-30c-2				-1
1	60007428		A	T	1046.24		167	1/1	1/1	1/1	0/1	0/0	0/1	0/1	intergenic_	MODIFIER	NONE			-1	CEP162-TBX18				-1
1	69551945		G	GT	4114.4		234	1/1	1/1	1/1	0/0	0/0	1/1	0/1	intergenic_	MODIFIER	NONE	n.null_nullinsT		-1	ENSSSCG00000018695-U6				-1
1	72866720		T	C	2092.71		154	1/1	1/1	1/1	0/1	0/0	1/1	1/1	intergenic_	MODIFIER	NONE			-1	7SK-ENSSSCG00000004345				-1
1	72942927		G	A	1057.09		125	1/1	1/1	1/1	0/1	0/0	1/1	1/1	intergenic_	MODIFIER	NONE			-1	7SK-U6				-1
1	74956905		T	TGTGTGT(	11354.34		207	1/1	1/1	1/1	0/1	0/0	1/1	0/1	intergenic_	MODIFIER	NONE	n.null_nullinsGTGTGT		-1	ENSSSCG00000004348-ENSSSCG00000004				-1
1	78454324		C	A	1217.68		149	1/1	1/1	1/1	0/1	0/0	1/1	0/1	intergenic_	MODIFIER	NONE			-1	GRIK2-HACE1				-1
1	78536625		GT	G	3973.33		227	1/1	1/1	1/1	0/1	0/0	1/1	0/1	intergenic_	MODIFIER	NONE			-1	GRIK2-HACE1				-1
1	79646811		GA	G	3225.28		209	1/1	1/1	1/1	0/1	0/0	1/1	0/1	intergenic_	MODIFIER	NONE			-1	GRIK2-HACE1				-1
1	80039756		T	C	1079.07		156	1/1	1/1	1/1	0/1	0/0	1/1	1/1	intergenic_	MODIFIER	NONE			-1	GRIK2-HACE1				-1
1	81615492		G	A	1344.85		115	1/1	1/1	1/1	0/1	0/0	1/1	1/1	intergenic_	MODIFIER	NONE			-1	ENSSSCG00000004369-U6				-1
1	87450249		A	G	2292.87		189	1/1	1/1	1/1	0/1	0/0	1/1	0/1	intergenic_	MODIFIER	NONE			-1	FYN-U6				-1
1	87750515		T	TTTTA	7274.94		233	1/1	1/1	1/1	0/1	0/0	1/1	0/1	intron_vari	MODIFIER	NONE	c.3765+357_3765+35		-1	ENSSSCG(	protein_coding		ENSSSCT0	26
1	87807921		A	G	1303.78		138	1/1	1/1	1/1	0/1	0/0	1/1	0/1	intron_vari	MODIFIER	NONE	c.112+773T>C		-1	ENSSSCG(	protein_coding		ENSSSCT0	1
1	1.37E+08		A	ACG	5570.43		214	1/1	1/1	1/1	0/1	0/0	1/1	0/0	intergenic_	MODIFIER	NONE	n.null_nullinsCG		-1	ENSSSCG00000004658-DUT				-1
1	1.59E+08		TTC	T	2430.88		203	1/1	1/1	1/1	0/1	0/0	1/1	1/1	intergenic_	MODIFIER	NONE	n.null_nulldelTC		-1	ENSSSCG00000004839-U6				-1
1	1.61E+08		T	TA	5489.87		197	1/1	1/1	1/1	0/0	0/0	1/1	1/1	upstream_	MODIFIER	NONE	n.-1_-1insT		-1	ENSSSCG(	miRNA		ENSSSCT0	-1
1	1.61E+08		T	TA	5489.87		197	1/1	1/1	1/1	0/0	0/0	1/1	1/1	intergenic_	MODIFIER	NONE	n.null_nullinsA		-1	ENSSSCG00000025864-ENSSSCG00000004				-1
1	2.45E+08		C	T	1198.84		170	1/1	1/1	1/1	0/1	0/0	1/1	1/1	intergenic_	MODIFIER	NONE			-1	ENSSSCG00000005227-KCNV2				-1
1	2.47E+08		T	C	1286.07		130	1/1	1/1	./.	0/1	0/0	1/1	1/1	intergenic_	MODIFIER	NONE			-1	U6-U6				-1
1	2.66E+08		C	T	6974.13		355	1/1	1/1	1/1	0/0	0/0	1/1	1/1	intron_vari	MODIFIER	NONE	c.-7+18761G>A		-1	ENSSSCG(	protein_coding		ENSSSCT0	2
1	2.72E+08		C	T	1895.63		180	1/1	1/1	1/1	0/1	0/0	1/1	1/1	intron_vari	MODIFIER	NONE	c.2358+11721G>A		-1	GRIN3A	protein_coding		ENSSSCT0	12
1	2.72E+08		T	C	1057.34		159	1/1	1/1	1/1	0/1	0/0	1/1	1/1	intron_vari	MODIFIER	NONE	c.699+6301A>G		-1	GRIN3A	protein_coding		ENSSSCT0	1
1	2.74E+08		G	T	1597.54		144	1/1	1/1	1/1	0/1	0/0	1/1	1/1	intergenic_	MODIFIER	NONE			-1	CYLC2-ENSSSCG00000005403				-1
1	2.74E+08		G	A	1445.8		160	1/1	1/1	1/1	0/1	0/0	1/1	1/1	intergenic_	MODIFIER	NONE			-1	CYLC2-ENSSSCG00000005403				-1
1	2.87E+08		A	G	1093.03		144	1/1	1/1	1/1	0/1	0/0	1/1	1/1	intergenic_	MODIFIER	NONE			-1	ENSSSCG00000026492-ENSSSCG00000025				-1
1	2.88E+08		G	A	1040.03		127	1/1	1/1	1/1	0/1	0/0	0/1	1/1	intergenic_	MODIFIER	NONE			-1	ENSSSCG00000005494-PAPPA				-1
1	2.88E+08		G	A	1034.03		146	1/1	1/1	1/1	0/0	0/0	1/1	1/1	intron_vari	MODIFIER	NONE	c.3154-8075G>A		-1	PAPPA	protein_coding		ENSSSCT0	11
1	2.89E+08		T	C	1382.87		166	1/1	1/1	1/1	0/1	0/0	1/1	1/1	intron_vari	MODIFIER	NONE	c.545+71578A>G		-1	ENSSSCG(	protein_coding		ENSSSCT0	6
2	8371729		CCA	C	1468.89		210	0/0	0/1	0/1	1/1	1/1	1/1	0/1	intergenic_	MODIFIER	NONE	n.null_nulldelCA		-1	ZBTB3-TTC9C				-1
2	14567383		A	G	1578.91		135	0/0	0/0	0/0	1/1	1/1	./.	1/1	intergenic_	MODIFIER	NONE			-1	ENSSSCG00000024728-ENSSSCG00000014				-1
2	19127176		CT	C	3059.78		258	0/0	0/0	0/0	1/1	1/1	1/1	0/1	intergenic_	MODIFIER	NONE			-1	TSPAN18-CD82				-1
2	61572733		C	G	1036.06		132	1/1	1/1	1/1	0/1	0/0	0/1	1/1	upstream_	MODIFIER	NONE	c.-70G>C		-1	PGLYRP2	protein_coding		ENSSSCT0	-1
2	61572733		C	G	1036.06		132	1/1	1/1	1/1	0/1	0/0	0/1	1/1	intergenic_	MODIFIER	NONE			-1	PGLYRP2-CYP4F22				-1
2	63077973		G	T	1013.9		161	1/1	1/1	1/1	0/1	0/0	0/0	1/1	intergenic_	MODIFIER	NONE			-1	ENSSSCG00000013810-ENSSSCG00000013				-1
2	63293848		T	TAC	3602.46		240	1/1	1/1	0/1	0/1	0/0	0/1	1/1	intergenic_	MODIFIER	NONE	n.null_nullinsAC		-1	ENSSSCG00000013809-ENSSSCG00000013				-1
2	67265991		AT	A	3801.14		236	1/1	1/1	1/1	0/0	0/0	0/1	1/1	3_prime_U	MODIFIER	NONE	c.*243delT		-1	ENSSSCG(	protein_coding		ENSSSCT0	5
2	72366396		C	G	1234.45		139	1/1	1/1	1/1	0/1	0/0	0/1	1/1	intergenic_	MODIFIER	NONE			-1	ENSSSCG00000013566-INSR				-1
2	85064375		G	T	1029.62		151	1/1	1/1	1/1	0/1	0/0	0/1	1/1	intergenic_	MODIFIER	NONE			-1	ENSSSCG00000024586-ENC1				-1
2	1.22E+08		A	AT	2147.06		211	1/1	1/1	1/1	0/1	0/0	1/1	1/1	intergenic_	MODIFIER	NONE	n.null_nullinsT		-1	ENSSSCG00000024866-ENSSSCG00000014				-1
2	1.23E+08		G	A	1870.51		154	1/1	1/1	1/1	0/1	0/0	1/1	1/1	intergenic_	MODIFIER	NONE			-1	ENSSSCG00000014212-KCNN2				-1
2	1.23E+08		T	C	1892.5		147	1/1	1/1	1/1	0/1	0/0	1/1	1/1	intergenic_	MODIFIER	NONE			-1	ENSSSCG00000014212-KCNN2				-1
2	1.24E+08		C	A	1092.28		158	1/1	1/1	1/1	0/1	0/0	1/1	1/1	intergenic_	MODIFIER	NONE			-1	KCNN2-TRIM36				-1
2	1.24E+08		T	C	1017.48		163	1/1	1/1	1/1	0/1	0/0	1/1	1/1	intergenic_	MODIFIER	NONE			-1	KCNN2-TRIM36				-1
2	1.41E+08		A	G	1608.77		120	1/1	1/1	1/1	0/1	0/0	1/1	1/1	intergenic_	MODIFIER	NONE			-1	ENSSSCG00000014294-ENSSSCG00000030				-1
2	1.41E+08		G	GA	2572.21		238	1/1	1/1	1/1	0/1	0/0	0/1	1/1	intergenic_	MODIFIER	NONE	n.null_nullinsA		-1	ENSSSCG00000030689-C5orf15				-1
2	1.42E+08		CA	C	3687.55		188	1/1	1/1	1/1	0/1	0/0	1/1	1/1	intergenic_	MODIFIER	NONE			-1	ENSSSCG00000030689-C5orf15				-1
2	1.43E+08		C	T	1438		125	1/1	1/1	1/1	0/1	0/0	1/1	1/1	intergenic_	MODIFIER	NONE			-1	H2AFY-ENSSSCG00000014309				-1
2	1.43E+08		T	C	1227.13		122	1/1	1/1	1/1	0/1	0/0	1/1	1/1	intergenic_	MODIFIER	NONE			-1	H2AFY-ENSSSCG00000014309				-1
2	1.43E+08		C	T	1209.97		178	1/1	1/1	1/1	0/1	0/0	1/1	1/1	intergenic_	MODIFIER	NONE			-1	H2AFY-ENSSSCG00000014309				-1
2	1.43E+08		A	T	1594.7		166	1/1	1/1	1/1	0/1	0/0	1/1	1/1	intergenic_	MODIFIER	NONE			-1	H2AFY-ENSSSCG00000014309				-1

Head	Note
#CHROM	Chromosome
POS	Position
ID	Identification
REF	Reference seq (Sscrofa 10.2)
ALT	Alternative sequence
QUAL	Quality
FILTER	
DP	Total depth
EFF[*].EFFE	Genetic element
EFF[*].IMP	Functional annotation
EFF[*].FUNCLASS	
EFF[*].CODON	
EFF[*].AA	
EFF[*].AA_LEN	
EFF[*].GENE	
EFF[*].BIOTYPE	
EFF[*].CODING	
EFF[*].TRID	
EFF[*].RANK	
./.	Not called
0/0	Homogeneous to REF
0/1	Heterogeneous to REF
1/1	Homogeneous to ALT

2	1.43E+08	A	G	1980.89	172	1/1	1/1	1/1	0/1	0/0	1/1	1/1	intergenic_	MODIFIER	NONE		-1	H2AFY-ENSSSCG00000014309			-1
2	1.43E+08	G	A	1673.21	181	1/1	1/1	1/1	0/1	0/0	1/1	1/1	intergenic_	MODIFIER	NONE		-1	H2AFY-ENSSSCG00000014309			-1
2	1.43E+08	G	C	1135.82	168	1/1	1/1	1/1	0/1	0/0	1/1	1/1	intergenic_	MODIFIER	NONE		-1	H2AFY-ENSSSCG00000014309			-1
2	1.5E+08	GGAA	G	4881.88	196	1/1	1/1	1/1	0/1	0/0	1/1	0/1	intergenic_	MODIFIER	NONE	n.null_nulldelGAA	-1	U5-FGF1			-1
2	1.56E+08	GT	G	3315.31	211	1/1	1/1	1/1	0/0	0/0	1/1	1/1	intergenic_	MODIFIER	NONE		-1	SPINK6-ENSSSCG00000030055			-1
2	1.58E+08	C	CA	2574.2	234	1/1	1/1	1/1	0/1	0/0	0/1	0/1	intergenic_	MODIFIER	NONE	n.null_nullinsA	-1	ARHGEF37-ssc-mir-378-1			-1
2	1.62E+08	T	C	1135.86	145	1/1	1/1	1/1	0/1	0/0	0/1	1/1	intron_var	MODIFIER	NONE	c.207-182A>G	-1	ENSSSCG(	protein_coding	ENSSSCT0	3
3	25213842	G	A	1519.45	174	0/0	0/1	0/1	1/1	1/1	0/1	1/1	intergenic_	MODIFIER	NONE		-1	ENSSSCG00000007847-ENSSSCG00000007			-1
3	27990223	G	A	1259.01	155	0/0	0/1	0/1	1/1	1/1	0/1	1/1	intergenic_	MODIFIER	NONE		-1	ENSSSCG00000007872-5S_rRNA			-1
3	29467453	G	A	1111.54	208	0/0	0/1	0/1	1/1	1/1	0/1	1/1	intron_var	MODIFIER	NONE	c.289+10436G>A	-1	CPPED1	protein_coding	ENSSSCT0	2
3	32430658	C	A	1259.52	132	0/0	0/1	0/1	1/1	1/1	0/1	1/1	intergenic_	MODIFIER	NONE		-1	LITAF-ENSSSCG00000028743			-1
3	33134141	C	T	1105.93	145	0/0	1/1	0/1	1/1	1/1	0/1	1/1	intron_var	MODIFIER	NONE	c.376+331C>T	-1	ENSSSCG(	protein_coding	ENSSSCT0	5
3	52550229	T	C	1559.23	143	0/0	0/1	0/1	1/1	1/1	1/1	0/1	intergenic_	MODIFIER	NONE		-1	MRPS9-ENSSSCG00000026126			-1
3	52550244	T	C	1518.25	143	0/0	0/1	0/1	1/1	1/1	1/1	0/1	intergenic_	MODIFIER	NONE		-1	MRPS9-ENSSSCG00000026126			-1
3	54769594	A	G	1046.19	168	0/0	0/1	0/1	1/1	1/1	1/1	0/1	intergenic_	MODIFIER	NONE		-1	IL1R1-ENSSSCG00000028331			-1
3	55829690	T	C	1156.22	161	0/0	0/1	0/1	1/1	1/1	1/1	0/1	intron_var	MODIFIER	NONE	c.*33+6539A>G	-1	RPL31	protein_coding	ENSSSCT0	4
3	55829690	T	C	1156.22	161	0/0	0/1	0/1	1/1	1/1	1/1	0/1	intron_var	MODIFIER	NONE	c.2549-2990T>C	-1	TBC1D8	protein_coding	ENSSSCT0	15
3	1.39E+08	C	T	1163.73	148	0/0	0/1	0/1	1/1	1/1	0/1	1/1	intergenic_	MODIFIER	NONE		-1	ENSSSCG00000025247-ASGR1			-1
3	4732833	AGG	A	3342.11	211	1/1	1/1	1/1	0/1	0/0	1/1	1/1	upstream_	MODIFIER	NONE	c.-88_-88delCC	-1	BETA-ACTI	protein_coding	ENSSSCT0	-1
3	4732833	AGG	A	3342.11	211	1/1	1/1	1/1	0/1	0/0	1/1	1/1	intergenic_	MODIFIER	NONE	n.null_nulldelGG	-1	BETA-ACTIN-ENSSSCG00000007586			-1
3	18714107	C	CA	1444.76	201	1/1	1/1	1/1	0/1	0/0	0/1	0/1	upstream_	MODIFIER	NONE	c.-309_-309insT	-1	TUFM	protein_coding	ENSSSCT0	-1
3	18714107	C	CA	1444.76	201	1/1	1/1	1/1	0/1	0/0	0/1	0/1	upstream_	MODIFIER	NONE	c.-795_-795insA	-1	SH2B1	protein_coding	ENSSSCT0	-1
3	18714107	C	CA	1444.76	201	1/1	1/1	1/1	0/1	0/0	0/1	0/1	intergenic_	MODIFIER	NONE	n.null_nullinsA	-1	TUFM-SH2B1			-1
3	1.38E+08	A	G	3281.05	164	1/1	1/1	1/1	0/1	0/0	1/1	1/1	intergenic_	MODIFIER	NONE		-1	CMPK2-U6			-1
3	1.38E+08	C	T	2352.53	123	1/1	1/1	1/1	0/1	0/0	1/1	1/1	intergenic_	MODIFIER	NONE		-1	CMPK2-U6			-1
3	1.38E+08	A	G	2109.53	115	1/1	1/1	1/1	0/1	0/0	1/1	1/1	intergenic_	MODIFIER	NONE		-1	CMPK2-U6			-1
3	1.38E+08	A	G	2083.53	115	1/1	1/1	1/1	0/1	0/0	1/1	1/1	intergenic_	MODIFIER	NONE		-1	CMPK2-U6			-1
3	1.38E+08	C	G	2113.33	116	1/1	1/1	1/1	0/1	0/0	1/1	1/1	intergenic_	MODIFIER	NONE		-1	CMPK2-U6			-1
3	1.38E+08	T	A	2020.54	116	1/1	1/1	1/1	0/1	0/0	1/1	1/1	intergenic_	MODIFIER	NONE		-1	CMPK2-U6			-1
3	1.38E+08	C	G	2051.33	116	1/1	1/1	1/1	0/1	0/0	1/1	1/1	intergenic_	MODIFIER	NONE		-1	CMPK2-U6			-1
3	1.38E+08	T	A	3692.8	192	1/1	1/1	1/1	0/1	0/0	1/1	1/1	intergenic_	MODIFIER	NONE		-1	CMPK2-U6			-1
3	1.38E+08	T	A	3595.05	191	1/1	1/1	1/1	0/1	0/0	1/1	1/1	intergenic_	MODIFIER	NONE		-1	CMPK2-U6			-1
4	4068921	T	TG	1661.78	218	0/0	0/1	0/1	0/1	1/1	1/1	1/1	intergenic_	MODIFIER	NONE	n.null_nullinsG	-1	ENSSSCG00000005938-5S_rRNA			-1
4	9068490	T	C	1288.22	135	0/0	0/1	0/1	1/1	1/1	0/1	1/1	intron_var	MODIFIER	NONE	c.169+46T>C	-1	OC90	protein_coding	ENSSSCT0	2
4	9068533	C	G	1109.13	130	0/0	0/1	0/1	1/1	1/1	0/1	1/1	intron_var	MODIFIER	NONE	c.169+89C>G	-1	OC90	protein_coding	ENSSSCT0	2
4	9084210	T	C	1281.3	160	0/0	0/1	0/1	1/1	1/1	0/1	1/1	intron_var	MODIFIER	NONE	c.1070+848T>C	-1	OC90	protein_coding	ENSSSCT0	12
4	9086006	T	C	1388.05	212	0/0	0/1	0/1	1/1	1/1	0/1	1/1	intron_var	MODIFIER	NONE	c.1071-180T>C	-1	OC90	protein_coding	ENSSSCT0	12
4	67994103	TTG	T	1837.94	214	0/0	0/0	0/1	1/1	1/1	0/1	1/1	intron_var	MODIFIER	NONE	c.814+7317_814+731	-1	STAU2	protein_coding	ENSSSCT0	6
4	74051994	T	C	1357.71	165	0/0	0/1	0/1	1/1	1/1	1/1	1/1	intergenic_	MODIFIER	NONE		-1	ENSSSCG00000006206-ENSSSCG00000023			-1
4	74052001	T	C	1158.98	164	0/0	0/1	0/1	1/1	1/1	1/1	1/1	intergenic_	MODIFIER	NONE		-1	ENSSSCG00000006206-ENSSSCG00000023			-1
4	84082675	GA	G	3538.89	265	0/0	0/0	0/1	1/1	1/1	1/1	1/1	intergenic_	MODIFIER	NONE		-1	SOX17-MRPL15			-1
4	86371133	A	G	1238.81	164	0/0	0/1	0/1	1/1	1/1	1/1	1/1	intergenic_	MODIFIER	NONE		-1	PCMTD1-SNAI2			-1
4	20791412	T	TC	3077.48	247	1/1	1/1	1/1	0/1	0/0	0/1	1/1	intron_var	MODIFIER	NONE	c.149-4470_149-4469	-1	ENSSSCG(	protein_coding	ENSSSCT0	1
4	60432696	C	T	1585.69	157	1/1	1/1	1/1	0/1	0/0	1/1	1/1	intergenic_	MODIFIER	NONE		-1	FABP5-PAG1			-1
4	1.34E+08	G	GA	1278.25	166	1/1	1/1	1/1	0/1	0/0	1/1	1/1	intergenic_	MODIFIER	NONE	n.null_nullinsA	-1	7SK-RWDD3			-1
5	9089370	C	CT	2230.04	216	0/0	0/0	0/0	1/1	1/1	1/1	1/1	intron_var	MODIFIER	NONE	c.3101-81_3101-80in:	-1	MYH9	protein_coding	ENSSSCT0	24
5	27974160	A	G	1202.27	146	0/0	0/1	0/1	1/1	1/1	0/1	1/1	intergenic_	MODIFIER	NONE		-1	U6-ENSSSCG00000000457			-1
5	69132974	GA	G	2087.8	234	0/0	0/0	0/1	1/1	1/1	0/1	1/1	intergenic_	MODIFIER	NONE		-1	PRMT8-ENSSSCG00000000734			-1
5	30837776	A	T	1709	151	1/1	1/1	1/1	0/1	0/0	1/1	0/1	intergenic_	MODIFIER	NONE		-1	ENSSSCG00000024879-ENSSSCG00000000			-1
5	33390843	G	A	1125.89	135	1/1	1/1	1/1	0/1	0/0	1/1	0/1	intron_var	MODIFIER	NONE	c.249+1087G>A	-1	HMGA2	protein_coding	ENSSSCT0	3
5	38824938	G	A	1083.32	179	1/1	1/1	1/1	0/1	0/0	1/1	0/1	intergenic_	MODIFIER	NONE		-1	TPH2-TRH-DE			-1
5	41628912	C	T	1768.76	190	1/1	1/1	1/1	0/1	0/0	1/1	0/1	intergenic_	MODIFIER	NONE		-1	ATXN7L3B-KCNC2			-1
5	65897789	C	T	1904.49	169	1/1	1/1	1/1	0/1	0/0	1/1	0/1	upstream_	MODIFIER	NONE	c.-324C>T	-1	ENSSSCG(	protein_coding	ENSSSCT0	-1
5	65897789	C	T	1904.49	169	1/1	1/1	1/1	0/1	0/0	1/1	0/1	intergenic_	MODIFIER	NONE		-1	SLC2A3-RBP5			-1
5	65897877	A	G	1231.62	178	1/1	1/1	1/1	0/1	0/0	1/1	0/1	upstream_	MODIFIER	NONE	c.-324A>G	-1	ENSSSCG(	protein_coding	ENSSSCT0	-1
5	65897877	A	G	1231.62	178	1/1	1/1	1/1	0/1	0/0	1/1	0/1	intergenic_	MODIFIER	NONE		-1	SLC2A3-RBP5			-1
5	67490871	G	GCA	3401.17	233	1/1	1/1	1/1	0/0	0/0	1/1	0/0	intergenic_	MODIFIER	NONE	n.null_nullinsCA	-1	ANO2-KV1.5			-1
6	2338761	T	C	1084.1	129	0/0	0/1	0/1	1/1	1/1	1/1	0/1	intergenic_	MODIFIER	NONE		-1	C16orf95-FOXL1			-1
6	3432569	C	G	1130.18	137	0/0	0/1	0/1	1/1	1/1	1/1	0/1	intergenic_	MODIFIER	NONE		-1	MTHFSD-COX4I1			-1
6	17071811	A	C	1049.52	1905	0/0	0/1	0/1	1/1	1/1	1/1	1/1	intergenic_	MODIFIER	NONE		-1	CDH3-RSPRY1			-1
6	29037648	A	G	1211.43	115	0/0	0/1	0/1	1/1	1/1	1/1	1/1	intergenic_	MODIFIER	NONE		-1	ENSSSCG00000002834-TOX3			-1
6	31093904	A	G	1081.53	127	0/0	0/1	0/1	1/1	1/1	1/1	1/1	intergenic_	MODIFIER	NONE		-1	ZNF423-N4BP1			-1
6	59974516	T	C	1514.54	134	0/0	0/1	0/1	1/1	1/1	1/1	1/1	intergenic_	MODIFIER	NONE		-1	ENSSSCG00000003361-C1orf174			-1
6	75048956	T	C	1056.88	139	0/0	0/1	0/1	1/1	1/1	0/1	1/1	intron_var	MODIFIER	NONE	c.1405-11327T>C	-1	KDM1A	protein_coding	ENSSSCT0	11
6	12326306	C	T	1793.25	182	1/1	1/1	1/1	0/1	0/0	0/1	1/1	intergenic_	MODIFIER	NONE		-1	LDHD-ZFP1			-1
6	52852324	A	G	1564.53	96	1/1	1/1	1/1	0/1	0/0	1/1	0/1	intergenic_	MODIFIER	NONE		-1	ENSSSCG00000003252-ENSSSCG00000003			-1
6	60387399	G	T	1149.24	150	1/1	1/1	1/1	0/1	0/0	1/1	0/1	intergenic_	MODIFIER	NONE		-1	DFFB-NPHP4			-1

6	61355577	A	C	1328.21	135	1/1	1/1	1/1	0/1	0/0	1/1	0/1	intergenic_	MODIFIER	NONE		-1	DFFB-NPHP4			-1
6	91356011	A	G	1204.48	142	1/1	1/1	1/1	0/1	0/0	1/1	0/1	intergenic_	MODIFIER	NONE		-1	ENSSSCG00000024963-ENSSSCG00000003			-1
6	1.2E+08	G	A	1308.65	170	1/1	1/1	1/1	0/1	0/0	1/1	1/1	intergenic_	MODIFIER	NONE		-1	ENSSSCG00000026170-U6			-1
6	1.24E+08	T	G	2316.1	178	1/1	1/1	1/1	0/1	0/0	1/1	1/1	intergenic_	MODIFIER	NONE		-1	U1-ELTD1			-1
6	1.24E+08	T	A	1941.49	146	1/1	1/1	1/1	0/1	0/0	1/1	1/1	intergenic_	MODIFIER	NONE		-1	U1-ELTD1			-1
7	40876991	C	T	1149.98	119	1/1	1/1	1/1	0/1	0/0	1/1	0/1	intergenic_	MODIFIER	NONE		-1	ENSSSCG00000019053-LRFN2			-1
7	41560388	G	C	1341.28	153	1/1	1/1	1/1	0/1	0/0	0/1	0/1	intergenic_	MODIFIER	NONE		-1	ENSSSCG00000026127-ENSSSCG00000027			-1
7	44077767	C	T	1121.94	157	1/1	1/1	1/1	0/1	0/0	0/1	1/1	intron_vari	MODIFIER	NONE	c.100-106C>T	-1	ENSSSCG(	protein_coding	ENSSSCT0	1
7	51780524	T	C	1465.8	128	1/1	1/1	1/1	0/1	0/0	0/1	1/1	intergenic_	MODIFIER	NONE		-1	ENSSSCG00000025535-ENSSSCG00000001			-1
7	51780537	G	A	1255.87	122	1/1	1/1	1/1	0/1	0/0	0/1	1/1	intergenic_	MODIFIER	NONE		-1	ENSSSCG00000025535-ENSSSCG00000001			-1
7	57883231	C	T	2008.36	140	1/1	1/1	1/1	0/1	0/0	1/1	1/1	intergenic_	MODIFIER	NONE		-1	RPS17-PDE8A			-1
7	57885654	T	C	3066.06	160	1/1	1/1	1/1	0/1	0/0	1/1	1/1	intergenic_	MODIFIER	NONE		-1	RPS17-PDE8A			-1
7	1.18E+08	G	A	1637.38	146	1/1	1/1	1/1	0/1	0/0	1/1	1/1	upstream_	MODIFIER	NONE	c.-8G>A	-1	ENSSSCG(	protein_coding	ENSSSCT0	-1
7	1.18E+08	G	A	1637.38	146	1/1	1/1	1/1	0/1	0/0	1/1	1/1	intergenic_	MODIFIER	NONE		-1	EFCAB11-ENSSSCG00000002431			-1
7	1.19E+08	A	G	1186.82	125	1/1	1/1	1/1	1/1	0/0	1/1	1/1	intron_vari	MODIFIER	NONE	c.581+7507T>C	-1	ENSSSCG(	protein_coding	ENSSSCT0	8
7	1.19E+08	G	C	1111.03	109	1/1	1/1	1/1	0/1	0/0	1/1	1/1	intergenic_	MODIFIER	NONE		-1	ENSSSCG00000002436-ENSSSCG00000024			-1
7	1.19E+08	G	C	1316.83	120	1/1	1/1	1/1	0/1	0/0	1/1	1/1	intergenic_	MODIFIER	NONE		-1	ENSSSCG00000002436-ENSSSCG00000024			-1
7	1.21E+08	C	T	1917.18	143	1/1	1/1	1/1	0/1	0/0	0/1	0/1	intergenic_	MODIFIER	NONE		-1	SLC24A4-ENSSSCG00000002451			-1
7	1.21E+08	C	T	1283.56	146	1/1	1/1	1/1	1/1	0/0	1/1	0/1	intron_vari	MODIFIER	NONE	c.246+3246C>T	-1	ENSSSCG(	protein_coding	ENSSSCT0	2
8	5768168	AG	A	1536.66	196	0/0	0/1	0/1	1/1	1/1	0/1	1/1	intergenic_	MODIFIER	NONE		-1	TMEM128-LYAR			-1
8	21945383	A	G	1116.71	139	0/0	0/1	1/1	1/1	1/1	0/1	1/1	intergenic_	MODIFIER	NONE		-1	7SK-ENSSSCG00000008763			-1
8	21945384	T	C	1257.37	140	0/0	0/1	1/1	1/1	1/1	1/1	1/1	intergenic_	MODIFIER	NONE		-1	7SK-ENSSSCG00000008763			-1
8	23009229	CG	C	1080.68	241	0/0	0/0	0/0	1/1	1/1	1/1	1/1	intergenic_	MODIFIER	NONE		-1	ENSSSCG00000008763-PCDH7			-1
8	26874123	A	C	1342.97	136	0/0	0/1	0/1	1/1	1/1	1/1	1/1	intergenic_	MODIFIER	NONE		-1	PCDH7-ENSSSCG00000008767			-1
8	32890209	C	CT	1085.9	213	0/0	0/1	0/1	1/1	1/1	0/1	0/1	intron_vari	MODIFIER	NONE	c.406+1180_406+118	-1	N4BP2	protein_coding	ENSSSCT0	2
8	33762665	GT	G	1159.43	216	0/0	1/1	1/1	1/1	1/1	1/1	0/1	intergenic_	MODIFIER	NONE		-1	NSUN7-U6			-1
8	39555553	T	TTA	4723.47	227	0/0	0/0	0/0	1/1	1/1	1/1	1/1	intron_vari	MODIFIER	NONE	c.2411+1756_2411+1	-1	CORIN	protein_coding	ENSSSCT0	16
8	39652185	GAGA	G	1633.46	200	0/0	0/0	0/0	1/1	1/1	0/1	0/1	intron_vari	MODIFIER	NONE	c.1012+13959_1012+	-1	CORIN	protein_coding	ENSSSCT0	5
8	45200463	CT	C	2816.01	228	0/0	0/1	0/0	1/1	1/1	1/1	1/1	intergenic_	MODIFIER	NONE		-1	ENSSSCG00000024269-CPE			-1
8	9266264	GT	G	2435.45	202	1/1	1/1	1/1	0/1	0/0	0/1	0/1	intergenic_	MODIFIER	NONE		-1	BOD1L1-CPEB2			-1
8	9831694	A	T	1615.79	118	1/1	1/1	1/1	0/1	0/0	1/1	1/1	intron_vari	MODIFIER	NONE	c.403+5675A>T	-1	CPEB2	protein_coding	ENSSSCT0	4
8	75790028	G	A	3313.66	386	1/1	1/1	1/1	0/0	0/0	0/1	0/0	intergenic_	MODIFIER	NONE		-1	SDAD1-CXCL9			-1
8	1.38E+08	AT	A	2739.72	169	1/1	1/1	1/1	0/0	0/0	0/1	1/1	intron_vari	MODIFIER	NONE	c.1510-27278delA	-1	ENSSSCG(	protein_coding	ENSSSCT0	2
9	4683739	A	G	1065.02	152	0/0	1/1	0/1	1/1	1/1	1/1	1/1	intergenic_	MODIFIER	NONE		-1	ENSSSCG00000014673-TRIM6			-1
9	55718387	A	G	1027.08	160	0/0	0/1	0/1	1/1	1/1	1/1	0/1	intergenic_	MODIFIER	NONE		-1	ENSSSCG00000015140-ENSSSCG00000015			-1
9	55737157	G	T	1022.89	124	0/0	0/1	0/1	1/1	1/1	1/1	1/1	intergenic_	MODIFIER	NONE		-1	ENSSSCG00000015140-ENSSSCG00000015			-1
9	56254834	T	A	2035.29	138	0/0	0/1	0/1	1/1	1/1	1/1	1/1	intergenic_	MODIFIER	NONE		-1	ENSSSCG00000015162-ENSSSCG00000025			-1
9	58075484	G	A	1752.15	167	0/0	0/1	0/1	1/1	1/1	1/1	0/1	intergenic_	MODIFIER	NONE		-1	TMEM218-ENSSSCG00000015211			-1
9	1E+08	T	TGG	1325.41	223	0/0	0/0	0/1	1/1	1/1	0/1	1/1	intron_vari	MODIFIER	NONE	c.11403+301_11403+	-1	DNAH11	protein_coding	ENSSSCT0	71
9	1E+08	G	A	1425.94	173	0/0	0/1	0/1	1/1	1/1	0/1	1/1	intron_vari	MODIFIER	NONE	c.12103-726G>A	-1	DNAH11	protein_coding	ENSSSCT0	75
9	1E+08	A	C	1166.23	168	0/0	0/1	0/1	1/1	1/1	0/1	1/1	intron_vari	MODIFIER	NONE	c.12103-708A>C	-1	DNAH11	protein_coding	ENSSSCT0	75
9	1.01E+08	A	G	1067.48	130	0/0	0/1	0/1	1/1	1/1	0/1	1/1	upstream_	MODIFIER	NONE	c.-64A>G	-1	IL6	protein_coding	ENSSSCT0	-1
9	1.01E+08	A	G	1067.48	130	0/0	0/1	0/1	1/1	1/1	0/1	1/1	intergenic_	MODIFIER	NONE		-1	TOMM7-IL6			-1
9	1.01E+08	CG	C	1229.56	254	0/0	0/1	0/0	1/1	1/1	0/0	0/1	intergenic_	MODIFIER	NONE		-1	IL6-FAM126A			-1
9	1.06E+08	C	CAT	3026.45	211	1/1	1/1	1/1	0/1	0/0	1/1	1/1	intron_vari	MODIFIER	NONE	c.113-6918_113-6917	-1	ENSSSCG(	protein_coding	ENSSSCT0	1
9	1.11E+08	A	G	1137.08	137	1/1	1/1	1/1	0/1	0/0	1/1	1/1	intergenic_	MODIFIER	NONE		-1	GNAT3-GNAI1			-1
9	1.11E+08	T	C	1197.71	139	1/1	1/1	1/1	0/1	0/0	1/1	1/1	intergenic_	MODIFIER	NONE		-1	GNAT3-GNAI1			-1
10	29938018	A	C	3839.15	362	0/0	0/1	0/1	1/1	1/1	0/1	1/1	intergenic_	MODIFIER	NONE		-1	ENSSSCG00000010935-ENSSSCG00000010			-1
10	45881163	T	C	3118.53	370	0/0	0/0	0/0	1/1	1/1	0/1	0/1	intron_vari	MODIFIER	NONE	c.6324+407A>G	-1	SVIL	protein_coding	ENSSSCT0	35
10	55452335	A	AT	1332.27	255	0/0	0/1	0/1	0/1	1/1	0/1	0/1	intergenic_	MODIFIER	NONE	n.null_nullinsT	-1	GPR158-THNSL1			-1
10	64031898	A	G	1811.04	138	0/0	0/1	0/1	1/1	1/1	1/1	1/1	intergenic_	MODIFIER	NONE		-1	GJD4-CCDC3			-1
10	8592670	A	G	2281.1	201	1/1	1/1	1/1	0/1	0/0	1/1	1/1	intergenic_	MODIFIER	NONE		-1	ENSSSCG00000025480-ESRRG			-1
10	8592671	G	C	2100.68	200	1/1	1/1	1/1	0/1	0/0	1/1	1/1	intergenic_	MODIFIER	NONE		-1	ENSSSCG00000025480-ESRRG			-1
10	8592675	G	A	2054.37	202	1/1	1/1	1/1	0/1	0/0	1/1	1/1	intergenic_	MODIFIER	NONE		-1	ENSSSCG00000025480-ESRRG			-1
10	26520830	AGG	A	2571.39	157	1/1	1/1	1/1	0/1	0/0	1/1	1/1	intergenic_	MODIFIER	NONE	n.null_nulldelGG	-1	ssc-mir-181a-1-NR5A2			-1
10	26756700	A	G	1021.36	127	1/1	1/1	1/1	0/1	0/0	1/1	1/1	intergenic_	MODIFIER	NONE		-1	ssc-mir-181a-1-NR5A2			-1
10	26756701	A	G	1018.12	127	1/1	1/1	1/1	0/1	0/0	1/1	1/1	intergenic_	MODIFIER	NONE		-1	ssc-mir-181a-1-NR5A2			-1
10	32789851	A	T	1218.3	134	1/1	1/1	1/1	0/1	0/0	1/1	0/1	intergenic_	MODIFIER	NONE		-1	DAPK1-ZCCHC6			-1
10	32789894	T	A	2168.52	151	1/1	1/1	1/1	0/1	0/0	1/1	0/1	intergenic_	MODIFIER	NONE		-1	DAPK1-ZCCHC6			-1
10	32906608	A	G	4906.19	309	1/1	1/1	1/1	0/1	0/0	1/1	0/1	intergenic_	MODIFIER	NONE		-1	DAPK1-ZCCHC6			-1
10	38853301	TAA	T	3562.76	182	1/1	1/1	1/1	0/0	0/0	1/1	0/0	intergenic_	MODIFIER	NONE	n.null_nulldelAA	-1	SNORA31-U6			-1
10	42794101	G	A	1261.29	152	1/1	1/1	1/1	0/1	0/0	1/1	0/1	intergenic_	MODIFIER	NONE		-1	ENSSSCG00000029287-ENSSSCG00000030			-1
10	42818926	T	C	2018.35	151	1/1	1/1	1/1	1/1	0/0	1/1	0/1	intergenic_	MODIFIER	NONE		-1	ENSSSCG00000029287-ENSSSCG00000030			-1
10	44424052	G	A	2388.15	279	1/1	1/1	1/1	0/1	0/0	1/1	0/0	intergenic_	MODIFIER	NONE		-1	BAMBI-ENSSSCG00000011017			-1
10	44424058	T	C	2818.19	289	1/1	1/1	1/1	0/1	0/0	1/1	0/0	intergenic_	MODIFIER	NONE		-1	BAMBI-ENSSSCG00000011017			-1
10	51413315	G	GA	1716.06	184	1/1	1/1	1/1	0/1	0/0	1/1	0/0	upstream_	MODIFIER	NONE	c.-150_-150insA	-1	NMT2	protein_coding	ENSSSCT0	-1
10	51413315	G	GA	1716.06	184	1/1	1/1	1/1	0/1	0/0	1/1	0/0	intergenic_	MODIFIER	NONE	n.null_nullinsA	-1	U6-NMT2			-1

10	52511030	A	G	1075.31	137	1/1	1/1	1/1	0/1	0/0	1/1	1/1	intergenic_	MODIFIER NONE		-1	MRPL51-ENSSSCG00000028447			-1
10	52511040	A	C	1221.41	150	1/1	1/1	1/1	0/1	0/0	1/1	0/1	intergenic_	MODIFIER NONE		-1	MRPL51-ENSSSCG00000028447			-1
11	26161070	A	G	1055.38	162	0/0	0/1	0/0	1/1	1/1	0/1	0/1	intergenic_	MODIFIER NONE		-1	ENSSSCG00000024707-RGCC			-1
11	71093622	A	T	1221.43	211	0/0	0/1	0/1	1/1	1/1	0/1	1/1	intron_var	MODIFIER NONE	c.273+592A>T	-1	ENSSSCG(	protein_coding	ENSSSCT0	2
11	80970713	G	A	1544.19	140	0/0	0/1	0/1	1/1	1/1	0/1	1/1	intergenic_	MODIFIER NONE		-1	ENSSSCG00000026317-EFNB2			-1
12	39592974	T	TC	1977.15	262	1/1	0/1	1/1	0/0	0/0	0/1	0/1	upstream_	MODIFIER NONE	c.-87_-87insG	-1	ZNHIT3	protein_coding	ENSSSCT0	-1
12	39592974	T	TC	1977.15	262	1/1	0/1	1/1	0/0	0/0	0/1	0/1	intergenic_	MODIFIER NONE	n.null_nullinsC	-1	ZNHIT3-CA4			-1
13	403773	C	CA	3243.74	202	0/0	0/0	0/1	1/1	1/1	1/1	1/1	intron_var	MODIFIER NONE	c.180+44121_180+44	-1	ENSSSCG(	protein_coding	ENSSSCT0	1
13	1.34E+08	G	A	1307.6	143	0/0	0/1	0/1	1/1	1/1	1/1	0/1	intergenic_	MODIFIER NONE		-1	RFC4-MASP1			-1
13	1.87E+08	G	GA	1764.93	238	0/0	0/1	0/1	1/1	1/1	1/1	1/1	intergenic_	MODIFIER NONE	n.null_nullinsA	-1	ROBO1-ENSSSCG00000012002			-1
13	2.15E+08	C	CGGGAGA	10414.89	225	0/0	0/1	0/1	1/1	1/1	1/1	0/1	intergenic_	MODIFIER NONE	n.null_nullinsGGGAG/	-1	ENSSSCG00000012078-ENSSSCG00000012			-1
13	17017335	A	C	1424.72	135	1/1	1/1	1/1	0/1	0/0	0/1	0/1	intergenic_	MODIFIER NONE		-1	AZI2-ENSSSCG00000024265			-1
13	86845406	T	C	1103.1	154	1/1	1/1	1/1	0/1	0/0	1/1	1/1	intron_var	MODIFIER NONE	c.*132-88864A>G	-1	CEP70	protein_coding	ENSSSCT0	18
13	88109145	A	G	1222.27	180	1/1	1/1	1/1	0/1	0/0	1/1	1/1	intron_var	MODIFIER NONE	c.185+2599T>C	-1	ENSSSCG(	protein_coding	ENSSSCT0	1
13	1E+08	A	AT	1284.7	222	1/1	1/1	1/1	0/1	0/0	1/1	1/1	intergenic_	MODIFIER NONE	n.null_nullinsT	-1	ENSSSCG00000026131-ENSSSCG00000011			-1
13	1.13E+08	A	AAC	4587.75	254	1/1	1/1	1/1	0/1	0/0	1/1	1/1	intergenic_	MODIFIER NONE	n.null_nullinsAC	-1	U6-SI			-1
13	1.85E+08	A	G	1810.41	146	1/1	1/1	1/1	0/1	0/0	1/1	1/1	intergenic_	MODIFIER NONE		-1	ENSSSCG00000029384-ROBO1			-1
13	1.85E+08	G	C	1842.45	148	1/1	1/1	1/1	0/1	0/0	1/1	1/1	intergenic_	MODIFIER NONE		-1	ENSSSCG00000029384-ROBO1			-1
13	1.88E+08	A	G	1522	143	1/1	1/1	1/1	0/1	0/0	1/1	0/1	intergenic_	MODIFIER NONE		-1	ENSSSCG00000012002-LIPI			-1
13	1.88E+08	G	C	1399.49	126	1/1	1/1	1/1	0/1	0/0	1/1	0/1	intergenic_	MODIFIER NONE		-1	ENSSSCG00000012002-LIPI			-1
13	1.88E+08	G	T	1381.49	128	1/1	1/1	1/1	0/1	0/0	1/1	0/1	intergenic_	MODIFIER NONE		-1	ENSSSCG00000012002-LIPI			-1
13	1.99E+08	G	A	1236.27	174	1/1	1/1	1/1	0/1	0/0	1/1	0/1	intergenic_	MODIFIER NONE		-1	ENSSSCG00000012021-ssc-mir-155			-1
13	2.06E+08	A	C	1909.65	109	1/1	1/1	0/1	1/1	0/0	1/1	1/1	intergenic_	MODIFIER NONE		-1	C21orf62-U6			-1
14	62454044	GT	G	2509.49	237	0/0	0/1	0/1	1/1	1/1	1/1	0/1	intron_var	MODIFIER NONE	c.3350+733delA	-1	SIPA1L2	protein_coding	ENSSSCT0	11
14	2347666	C	G	1609.36	139	1/1	1/1	1/1	0/1	0/0	1/1	1/1	intergenic_	MODIFIER NONE		-1	U6-U6			-1
14	2347673	G	A	1612.36	137	1/1	1/1	1/1	0/1	0/0	1/1	1/1	intergenic_	MODIFIER NONE		-1	U6-U6			-1
14	9485038	CA	C	3348.82	212	1/1	1/1	1/1	0/0	0/0	0/1	1/1	intergenic_	MODIFIER NONE		-1	ADAM28-ADAMDEC1			-1
14	72609770	A	AT	1430.63	261	1/1	1/1	1/1	0/0	0/0	1/1	0/0	intergenic_	MODIFIER NONE	n.null_nullinsT	-1	ENSSSCG00000028393-CTNNA3			-1
14	72730900	C	T	1915.57	151	1/1	1/1	1/1	0/1	0/0	1/1	0/1	intergenic_	MODIFIER NONE		-1	ENSSSCG00000028393-CTNNA3			-1
14	73691524	G	GAA	2039.67	211	1/1	1/1	1/1	0/1	0/0	0/1	0/1	intergenic_	MODIFIER NONE	n.null_nullinsAA	-1	ENSSSCG00000028393-CTNNA3			-1
14	74084160	C	A	1497.43	157	1/1	1/1	1/1	0/1	0/0	1/1	0/1	intergenic_	MODIFIER NONE		-1	ENSSSCG00000028393-CTNNA3			-1
14	88418838	C	T	1761.51	166	1/1	1/1	1/1	0/1	0/0	1/1	0/1	intergenic_	MODIFIER NONE		-1	ENSSSCG00000010330-ANXA11			-1
14	97440802	C	T	1038.36	129	1/1	1/1	1/1	0/1	0/0	1/1	1/1	intergenic_	MODIFIER NONE		-1	WDFY4-ENSSSCG00000010385			-1
14	97440803	A	G	1103.34	130	1/1	1/1	1/1	0/1	0/0	1/1	1/1	intergenic_	MODIFIER NONE		-1	WDFY4-ENSSSCG00000010385			-1
15	19740207	C	A	1451.84	147	0/0	0/1	0/1	1/1	1/1	1/1	1/1	intergenic_	MODIFIER NONE		-1	ENSSSCG00000029118-ENSSSCG00000015			-1
15	24436358	T	C	1285.4	173	0/0	0/1	0/1	1/1	1/1	1/1	1/1	intergenic_	MODIFIER NONE		-1	U6-ENSSSCG00000026504			-1
15	29972309	G	A	1001.36	145	0/0	0/1	0/1	1/1	1/1	1/1	0/1	intergenic_	MODIFIER NONE		-1	GYPC-ENSSSCG00000023800			-1
15	34676931	C	T	1022.27	153	0/0	0/1	0/1	1/1	1/1	1/1	0/1	upstream_	MODIFIER NONE	c.-76C>T	-1	ENSSSCG(	protein_coding	ENSSSCT0	-1
15	34676931	C	T	1022.27	153	0/0	0/1	0/1	1/1	1/1	1/1	0/1	intergenic_	MODIFIER NONE		-1	TSN-ENSSSCG00000015730			-1
15	38157850	T	TA	1519.66	225	0/0	0/1	0/1	1/1	1/1	1/1	1/1	intergenic_	MODIFIER NONE	n.null_nullinsA	-1	ENSSSCG00000015747-ENSSSCG00000029			-1
15	40325994	A	C	1181.59	141	0/0	0/1	0/1	1/1	1/1	1/1	0/1	intergenic_	MODIFIER NONE		-1	ENSSSCG00000015748-U6			-1
15	65506863	G	A	1070.23	133	0/0	0/1	0/1	1/1	1/1	1/1	1/1	intergenic_	MODIFIER NONE		-1	SNORA19-HS6ST1			-1
15	65506911	C	T	1551.78	156	0/0	0/1	0/1	1/1	1/1	1/1	1/1	intergenic_	MODIFIER NONE		-1	SNORA19-HS6ST1			-1
15	1.09E+08	A	C	1425.75	160	0/0	0/1	0/1	1/1	1/1	1/1	1/1	intergenic_	MODIFIER NONE		-1	TMEFF2-STK17B			-1
15	1.28E+08	G	A	1458.16	152	0/0	0/1	0/1	1/1	1/1	0/1	0/1	intergenic_	MODIFIER NONE		-1	SPAG16-ENSSSCG00000029683			-1
15	1.28E+08	T	C	1488.16	152	0/0	0/1	0/1	1/1	1/1	0/1	0/1	intergenic_	MODIFIER NONE		-1	SPAG16-ENSSSCG00000029683			-1
15	1.36E+08	G	T	1526.59	126	0/0	0/1	0/1	1/1	1/1	1/1	1/1	intergenic_	MODIFIER NONE		-1	U6-SNORA31			-1
15	443360	T	C	1008.08	145	1/1	1/1	1/1	0/1	0/0	0/1	1/1	intergenic_	MODIFIER NONE		-1	ENSSSCG00000023716-U6			-1
15	443446	A	T	1141.8	127	1/1	1/1	1/1	0/1	0/0	0/1	1/1	intergenic_	MODIFIER NONE		-1	ENSSSCG00000023716-U6			-1
15	516298	T	C	1728.49	166	1/1	1/1	1/1	0/1	0/0	0/1	1/1	intergenic_	MODIFIER NONE		-1	ENSSSCG00000023716-U6			-1
15	1.46E+08	C	T	1712.54	136	1/1	1/1	1/1	0/1	0/0	1/1	1/1	intergenic_	MODIFIER NONE		-1	COPS7B-NPPC			-1
15	1.46E+08	C	G	1087.59	116	1/1	1/1	1/1	0/1	0/0	1/1	./.	intergenic_	MODIFIER NONE		-1	COPS7B-NPPC			-1
15	1.47E+08	A	T	1064.48	143	1/1	1/1	1/1	0/1	0/0	1/1	1/1	intergenic_	MODIFIER NONE		-1	ECEL1-ENSSSCG00000024181			-1
15	1.47E+08	C	T	1946.63	140	1/1	1/1	1/1	0/1	0/0	1/1	1/1	intergenic_	MODIFIER NONE		-1	NGEF-ENSSSCG00000016297			-1
15	1.51E+08	C	T	2555.34	143	1/1	0/1	1/1	0/0	0/0	0/1	0/1	intergenic_	MODIFIER NONE		-1	ACKR3-COPS8			-1
15	1.52E+08	A	G	1580.99	150	1/1	1/1	1/1	0/1	0/0	0/1	0/1	intron_var	MODIFIER NONE	c.354+1314A>G	-1	ENSSSCG(	protein_coding	ENSSSCT0	7
15	1.54E+08	G	A	1083.57	293	1/1	1/1	1/1	0/1	0/0	0/1	1/1	intergenic_	MODIFIER NONE		-1	ENSSSCG00000024643-ENSSSCG00000029			-1
15	1.54E+08	C	A	1180.56	293	1/1	1/1	1/1	0/1	0/0	0/1	1/1	intergenic_	MODIFIER NONE		-1	ENSSSCG00000024643-ENSSSCG00000029			-1
15	1.54E+08	C	T	1229.31	299	1/1	1/1	1/1	0/1	0/0	0/1	1/1	intergenic_	MODIFIER NONE		-1	ENSSSCG00000024643-ENSSSCG00000029			-1
16	8032570	GA	G	2914.9	230	0/0	0/1	0/0	1/1	1/1	1/1	0/1	intergenic_	MODIFIER NONE		-1	7SK-CDH18			-1
16	20790534	A	G	1658.17	148	0/0	0/1	0/1	1/1	1/1	1/1	0/1	intergenic_	MODIFIER NONE		-1	AMACR-C1QTNF3			-1
16	20437451	T	C	1165.95	157	1/1	1/1	1/1	0/1	0/0	0/1	1/1	intron_var	MODIFIER NONE	c.1086-146A>G	-1	ADAMTS1	protein_coding	ENSSSCT0	8
16	20596069	C	T	1601.92	164	1/1	1/1	1/1	0/1	0/0	0/1	1/1	intergenic_	MODIFIER NONE		-1	ADAMTS12-ENSSSCG00000027886			-1
16	20596072	G	A	1397.99	157	1/1	1/1	1/1	0/1	0/0	0/1	1/1	intergenic_	MODIFIER NONE		-1	ADAMTS12-ENSSSCG00000027886			-1
16	20596086	G	A	1399.56	168	1/1	1/1	1/1	0/1	0/0	0/1	1/1	intergenic_	MODIFIER NONE		-1	ADAMTS12-ENSSSCG00000027886			-1
16	24919650	G	C	4563.38	343	1/1	1/1	1/1	0/0	0/0	0/1	0/1	intergenic_	MODIFIER NONE		-1	EGFLAM-LIFR			-1
16	51368450	T	A	1304.22	150	1/1	1/1	1/1	0/1	0/0	0/0	1/1	intron_var	MODIFIER NONE	c.-40-3851T>A	-1	ENSSSCG(	protein_coding	ENSSSCT0	1

16	74371260	A	G	1548.09	145	1/1	1/1	1/1	0/1	0/0	0/1	0/1	intergenic_	MODIFIER	NONE		-1	ENSSSCG00000017069-HAND1			-1
17	36984058	C	CT	1772.88	238	0/0	0/0	0/0	0/1	1/1	1/1	1/1	intergenic_	MODIFIER	NONE	n.null_nullinsT	-1	OXT-MRPS26			-1
17	40342407	C	T	1218.62	126	0/0	0/1	0/1	1/1	1/1	1/1	1/1	upstream_	MODIFIER	NONE	c.-1C>T	-1	ENSSSCG(	protein_coding	ENSSSCT0	-1
17	40342407	C	T	1218.62	126	0/0	0/1	0/1	1/1	1/1	1/1	1/1	intron_vari	MODIFIER	NONE	c.-172+1139C>T	-1	DUSP15	protein_coding	ENSSSCT0	3
17	40342423	A	T	1394.48	134	0/0	0/1	0/1	1/1	1/1	1/1	1/1	upstream_	MODIFIER	NONE	c.-1A>T	-1	ENSSSCG(	protein_coding	ENSSSCT0	-1
17	40342423	A	T	1394.48	134	0/0	0/1	0/1	1/1	1/1	1/1	1/1	intron_vari	MODIFIER	NONE	c.-172+1155A>T	-1	DUSP15	protein_coding	ENSSSCT0	3
17	40342441	T	G	1631.21	147	0/0	0/1	0/1	1/1	1/1	1/1	1/1	upstream_	MODIFIER	NONE	c.-1T>G	-1	ENSSSCG(	protein_coding	ENSSSCT0	-1
17	40342441	T	G	1631.21	147	0/0	0/1	0/1	1/1	1/1	1/1	1/1	intron_vari	MODIFIER	NONE	c.-172+1173T>G	-1	DUSP15	protein_coding	ENSSSCT0	3
17	43895202	T	A	1201.82	145	0/0	0/1	0/1	1/1	1/1	1/1	1/1	intron_vari	MODIFIER	NONE	c.24+870A>T	-1	ENSSSCG(	protein_coding	ENSSSCT0	1
17	610985	C	T	1399.86	141	1/1	1/1	1/1	0/1	0/0	1/1	1/1	intergenic_	MODIFIER	NONE		-1	ENSSSCG00000020727-LONRF1			-1
17	1091800	T	C	1624.73	160	1/1	1/1	1/1	0/1	0/0	1/1	1/1	intergenic_	MODIFIER	NONE		-1	ENSSSCG00000006970-ENSSSCG00000006			-1
17	3089563	G	T	1664.64	139	1/1	1/1	1/1	0/1	0/0	1/1	1/1	intergenic_	MODIFIER	NONE		-1	ssc-mir-383-TUSC3			-1
17	3089566	T	C	1645.72	136	1/1	1/1	1/1	0/1	0/0	1/1	1/1	intergenic_	MODIFIER	NONE		-1	ssc-mir-383-TUSC3			-1
17	6783031	T	C	1099.24	149	1/1	1/1	1/1	0/1	0/0	1/1	0/1	intergenic_	MODIFIER	NONE		-1	ENSSSCG00000006996-CH242-286D19.2			-1
17	12807473	A	G	1111.44	104	1/1	1/1	1/1	0/1	0/0	1/1	1/1	intergenic_	MODIFIER	NONE		-1	CHRNB3-SMIM19			-1
17	12857961	C	T	1370.74	140	1/1	1/1	1/1	0/1	0/0	1/1	1/1	intergenic_	MODIFIER	NONE		-1	SMIM19-SLC20A2			-1
18	40749240	G	GA	1047.25	198	0/0	0/1	0/1	1/1	1/1	1/1	0/1	intron_vari	MODIFIER	NONE	c.-141+11760_-141+:	-1	ELMO1	protein_coding	ENSSSCT0	14
18	46377954	A	G	1019.53	97	0/0	0/1	0/1	1/1	1/1	1/1	1/1	intergenic_	MODIFIER	NONE		-1	PAC1-GHRHR			-1
18	1133221	C	G	1099.8	119	1/1	1/1	1/1	1/1	0/0	1/1	1/1	intergenic_	MODIFIER	NONE		-1	ENSSSCG00000026661-PTPRQ			-1
18	1139071	A	G	1175.86	159	1/1	1/1	1/1	0/1	0/0	1/1	0/1	intergenic_	MODIFIER	NONE		-1	ENSSSCG00000026661-PTPRQ			-1
18	1203153	G	A	1499.59	165	1/1	1/1	1/1	0/1	0/0	1/1	0/1	intergenic_	MODIFIER	NONE		-1	ENSSSCG00000026661-PTPRQ			-1
18	17502037	AG	A	1799.14	241	1/1	1/1	1/1	0/0	0/0	1/1	1/1	intergenic_	MODIFIER	NONE		-1	CHCHD3-PODXL			-1
