# Supplementary material for: No excessive mutations in transcription activator-like effector nuclease-mediated α-1,3-galactosyltransferase knockout Yucatan miniature pigs
Source: Asian-Australas J Anim Sci. 2019 Aug 23;33(2):360–72. doi: 10.5713/ajas.19.0480 (PMC6946973; doi:10.5713/ajas.19.0480)
Supplement: Supplementary file 5 [file ajas-19-0480-suppl5.pdf]

Supplementary Table 5. Variant calls of wild type strain control WT2

#CHROM	POS	ID	REF	ALT	QUAL	FILTER	DP	Donor	CJ1	CB1	CB3	WT1	WT2	WT3	EFF[*].EFFECT	EFF[*].IMPACT	EFF[*].FUNCLASS	EFF[*].CODON	EFF[*].AA	EFF[*].AA_LEN	EFF[*].GENE	EFF[*].BIOTYPE	EFF[*].CODING	EFF[*].TRID	EFF[*].RANK
1	1.09E+08		C	CA	1282.4		229	0/0	0/0	0/0	1/1	0/1	1/1	1/1	upstream_	MODIFIER	NONE	n.-1_-1insA		-1	SCARNA17	snoRNA		ENSSSCT0	-1
1	1.09E+08		C	CA	1282.4		229	0/0	0/0	0/0	1/1	0/1	1/1	1/1	upstream_	MODIFIER	NONE	n.-1_-1insA		-1	SCARNA18	snoRNA		ENSSSCT0	-1
1	1.09E+08		C	CA	1282.4		229	0/0	0/0	0/0	1/1	0/1	1/1	1/1	intron_vari	MODIFIER	NONE	c.16+1468_16+1469i		-1	ACAA2	protein_coding		ENSSSCT0	1
1	1.82E+08		T	C	4023.92		210	0/0	0/1	0/1	1/1	1/1	1/1	1/1	intergenic_	MODIFIER	NONE			-1	U2-DIS3L				-1
1	1.9E+08		A	G	1167.65		122	0/0	1/1	0/1	1/1	1/1	1/1	1/1	intergenic_	MODIFIER	NONE			-1	5S_rRNA-U6				-1
1	2.17E+08		GAA	G	1834.22		209	0/0	0/1	0/1	0/1	0/1	1/1	0/1	intergenic_	MODIFIER	NONE	n.null_nulldelAA		-1	GPHB5-ENSSSCG00000005118				-1
1	19885280		C	T	1176.07		149	1/1	1/1	1/1	0/1	0/1	0/0	1/1	intergenic_	MODIFIER	NONE			-1	U6-SAMD5				-1
1	19885288		A	C	1300.5		148	1/1	1/1	1/1	0/1	0/1	0/0	1/1	intergenic_	MODIFIER	NONE			-1	U6-SAMD5				-1
1	19885296		T	C	1361.47		154	1/1	1/1	1/1	0/1	0/1	0/0	1/1	intergenic_	MODIFIER	NONE			-1	U6-SAMD5				-1
1	23161886		T	C	1543.19		172	1/1	1/1	1/1	0/1	0/1	0/0	0/1	intergenic_	MODIFIER	NONE			-1	ENSSSCG00000004123-ENSSSCG00000026				-1
1	23165345		T	C	1306.48		152	1/1	1/1	1/1	./.	0/1	0/0	0/1	intergenic_	MODIFIER	NONE			-1	ENSSSCG00000004123-ENSSSCG00000026				-1
1	1.23E+08		AT	A	2468.34		239	1/1	1/1	1/1	0/1	0/1	0/0	0/1	intergenic_	MODIFIER	NONE			-1	ANXA2-ENSSSCG00000004580				-1
1	1.66E+08		TG	T	4071.08		234	1/1	0/1	0/1	1/1	1/1	0/0	0/1	intergenic_	MODIFIER	NONE			-1	FBXO15-ENSSSCG00000025660				-1
1	2.2E+08		C	T	7330.54		340	1/1	1/1	1/1	1/1	1/1	0/0	1/1	intergenic_	MODIFIER	NONE			-1	U6-TUSC1				-1
1	2.22E+08		T	C	6677.54		264	1/1	1/1	1/1	1/1	1/1	0/0	1/1	intron_vari	MODIFIER	NONE	c.298+8250T>C		-1	ELAVL2	protein_coding		ENSSSCT0	4
1	2.25E+08		G	C	1317.39		287	1/1	1/1	1/1	1/1	1/1	0/0	1/1	intergenic_	MODIFIER	NONE			-1	IFN-ALPHA-11-CH242-414D14.1				-1
1	2.25E+08		G	A	1570.28		282	1/1	1/1	1/1	1/1	1/1	0/0	1/1	intergenic_	MODIFIER	NONE			-1	IFN-ALPHA-11-CH242-414D14.1				-1
1	2.25E+08		T	C	1720.74		220	1/1	1/1	1/1	1/1	1/1	0/0	1/1	intergenic_	MODIFIER	NONE			-1	IFN-ALPHA-11-CH242-414D14.1				-1
1	2.25E+08		A	G	1566.74		222	1/1	1/1	1/1	1/1	1/1	0/0	1/1	intergenic_	MODIFIER	NONE			-1	IFN-ALPHA-11-CH242-414D14.1				-1
1	2.25E+08		T	C	1727.73		219	1/1	1/1	1/1	1/1	1/1	0/0	1/1	intergenic_	MODIFIER	NONE			-1	IFN-ALPHA-11-CH242-414D14.1				-1
1	2.25E+08		C	A	4065.97		298	1/1	1/1	1/1	1/1	1/1	0/0	1/1	intron_vari	MODIFIER	NONE	c.399+10379C>A		-1	IFN-ALPH/	protein_coding		ENSSSCT0	3
1	2.29E+08		T	C	5339.64		288	1/1	1/1	1/1	1/1	1/1	0/0	1/1	intron_vari	MODIFIER	NONE	c.802+827A>G		-1	SH3GL2	protein_coding		ENSSSCT0	9
1	2.31E+08		A	G	5747.55		235	1/1	1/1	1/1	1/1	1/1	0/0	1/1	intergenic_	MODIFIER	NONE			-1	TTC39B-ZDHHC21				-1
1	2.31E+08		A	G	5654.54		233	1/1	1/1	1/1	1/1	1/1	0/0	1/1	intergenic_	MODIFIER	NONE			-1	TTC39B-ZDHHC21				-1
1	2.89E+08		C	T	1589.58		148	1/1	1/1	1/1	0/1	0/1	0/0	1/1	intergenic_	MODIFIER	NONE			-1	ENSSSCG00000005502-ENSSSCG00000027				-1
1	2.89E+08		A	T	1761.99		144	1/1	1/1	1/1	0/1	0/1	0/0	1/1	intergenic_	MODIFIER	NONE			-1	ENSSSCG00000005502-ENSSSCG00000027				-1
2	8371729		CCA	C	1468.89		210	0/0	0/1	0/1	1/1	1/1	1/1	0/1	intergenic_	MODIFIER	NONE	n.null_nulldelCA		-1	ZBTB3-TTC9C				-1
2	19127176		CT	C	3059.78		258	0/0	0/0	0/0	1/1	1/1	1/1	0/1	intergenic_	MODIFIER	NONE			-1	TSPAN18-CD82				-1
2	90267640		A	AAAAAAC	3886.24		216	0/0	0/1	0/1	0/1	0/1	1/1	0/0	intergenic_	MODIFIER	NONE	n.null_nullinsAAAAAC		-1	ENSSSCG00000026098-ENSSSCG00000014				-1
2	4845229		G	T	1173.19		123	1/1	1/1	1/1	1/1	1/1	0/0	1/1	upstream_	MODIFIER	NONE	c.-134C>A		-1	CTSF	protein_coding		ENSSSCT0	-1
2	4845229		G	T	1173.19		123	1/1	1/1	1/1	1/1	1/1	0/0	1/1	intergenic_	MODIFIER	NONE			-1	CTSF-CCDC87				-1
2	4933475		G	C	1296.18		157	1/1	1/1	1/1	1/1	1/1	0/0	1/1	intron_vari	MODIFIER	NONE	c.892+27G>C		-1	CTSF	protein_coding		ENSSSCT0	8
2	8870056		G	GTT	3275.88		172	1/1	0/1	1/1	1/1	1/1	0/0	1/1	intron_vari	MODIFIER	NONE	c.992-2239_992-2238		-1	ENSSSCG(	protein_coding		ENSSSCT0	4
2	29686631		AC	A	1116.53		156	1/1	1/1	1/1	1/1	1/1	0/0	1/1	intron_vari	MODIFIER	NONE	c.4147-2633delC		-1	KIAA1549I	protein_coding		ENSSSCT0	13
2	29689931		C	CA	1888.12		196	1/1	1/1	1/1	1/1	1/1	0/0	1/1	intron_vari	MODIFIER	NONE	c.4364+449_4364+45		-1	KIAA1549I	protein_coding		ENSSSCT0	14
2	48174967		TAC	T	4304.16		240	1/1	1/1	1/1	1/1	1/1	0/0	1/1	intron_vari	MODIFIER	NONE	c.-736+44382_-736+4		-1	SPON1	protein_coding		ENSSSCT0	6
2	49365996		TAG	T	4868.06		182	1/1	0/1	1/1	1/1	1/1	0/0	1/1	intergenic_	MODIFIER	NONE	n.null_nulldelAG		-1	BMAL1-ENSSSCG00000028546				-1
2	52136229		GGT	G	4506.26		196	1/1	1/1	1/1	1/1	1/1	0/0	1/1	intron_vari	MODIFIER	NONE	c.-3+5310_-3+5311d		-1	MRVI1	protein_coding		ENSSSCT0	1
2	52256122		TTG	T	5703.51		236	1/1	1/1	1/1	1/1	1/1	0/0	1/1	intron_vari	MODIFIER	NONE	c.2168+4071_2168+4		-1	MRVI1	protein_coding		ENSSSCT0	18
2	52910825		AT	A	2764.77		252	1/1	0/1	1/1	1/1	1/1	0/0	1/1	intron_vari	MODIFIER	NONE	c.496-5566delT		-1	ENSSSCG(	protein_coding		ENSSSCT0	4
2	54632899		C	T	1265.25		180	1/1	1/1	1/1	1/1	1/1	0/0	1/1	intergenic_	MODIFIER	NONE			-1	JMJD4-ENSSSCG00000024506				-1
2	55591681		A	G	1005.55		156	1/1	1/1	1/1	1/1	1/1	0/0	1/1	intergenic_	MODIFIER	NONE			-1	ENSSSCG00000025084-ENSSSCG00000028				-1
2	61255610		G	GT	2448.68		227	1/1	1/1	1/1	0/1	0/1	0/0	1/1	intergenic_	MODIFIER	NONE	n.null_nullinsT		-1	ENSSSCG00000013844-ENSSSCG00000013				-1
2	61714074		A	G	2278.19		161	1/1	1/1	1/1	0/1	0/1	0/0	1/1	intergenic_	MODIFIER	NONE			-1	CYP4F22-RASAL3				-1
2	62994051		T	A	2127.2		239	1/1	1/1	1/1	0/1	0/1	0/0	1/1	upstream_	MODIFIER	NONE	c.-1T>A		-1	ENSSSCG(	protein_coding		ENSSSCT0	-1
2	62994051		T	A	2127.2		239	1/1	1/1	1/1	0/1	0/1	0/0	1/1	intergenic_	MODIFIER	NONE			-1	ENSSSCG00000013811-ENSSSCG00000013				-1
2	63077973		G	T	1013.9		161	1/1	1/1	1/1	0/1	0/0	0/0	1/1	intergenic_	MODIFIER	NONE			-1	ENSSSCG00000013810-ENSSSCG00000013				-1
2	72365504		A	C	1300.74		141	1/1	1/1	1/1	0/1	0/1	0/0	1/1	intergenic_	MODIFIER	NONE			-1	ENSSSCG00000013566-INSR				-1
2	72699689		C	G	2618.15		785	1/1	1/1	1/1	0/1	0/1	0/0	1/1	missense_v	MODERAT	NONE	c.142C>G	p.Arg48Gl	807	EMR1	protein_coding		ENSSSCT0	1
2	72699689		C	G	2618.15		785	1/1	1/1	1/1	0/1	0/1	0/0	1/1	upstream_	MODIFIER	NONE	c.-1G>C		-1	ENSSSCG(	protein_coding		ENSSSCT0	-1
2	88881240		T	C	1672.9		179	1/1	1/1	1/1	0/1	0/1	0/0	1/1	intron_vari	MODIFIER	NONE	c.-207+1926T>C		-1	ENSSSCG(	protein_coding		ENSSSCT0	1
2	1.61E+08		A	C	1869.56		125	1/1	1/1	1/1	1/1	1/1	0/0	1/1	intergenic_	MODIFIER	NONE			-1	ENSSSCG00000028286-ENSSSCG00000025				-1
2	1.61E+08		AT	A	3070.32		222	1/1	1/1	1/1	1/1	1/1	0/0	1/1	intergenic_	MODIFIER	NONE			-1	ENSSSCG00000021614-ENSSSCG00000027				-1
3	52550229		T	C	1559.23		143	0/0	0/1	0/1	1/1	1/1	1/1	0/1	intergenic_	MODIFIER	NONE			-1	MRPS9-ENSSSCG00000026126				-1
3	52550244		T	C	1518.25		143	0/0	0/1	0/1	1/1	1/1	1/1	0/1	intergenic_	MODIFIER	NONE			-1	MRPS9-ENSSSCG00000026126				-1
3	54769594		A	G	1046.19		168	0/0	0/1	0/1	1/1	1/1	1/1	0/1	intergenic_	MODIFIER	NONE			-1	IL1R1-ENSSSCG00000028331				-1
3	55829690		T	C	1156.22		161	0/0	0/1	0/1	1/1	1/1	1/1	0/1	intron_vari	MODIFIER	NONE	c.*33+6539A>G		-1	RPL31	protein_coding		ENSSSCT0	4
3	55829690		T	C	1156.22		161	0/0	0/1	0/1	1/1	1/1	1/1	0/1	intron_vari	MODIFIER	NONE	c.2549-2990T>C		-1	TBC1D8	protein_coding		ENSSSCT0	15
3	1386148		T	TG	3196.14		235	1/1	1/1	1/1	1/1	1/1	0/0	1/1	intergenic_	MODIFIER	NONE	n.null_nullinsG		-1	ENSSSCG00000027961-ELFN1				-1
3	4447954		A	G	1263.86		122	1/1	1/1	1/1	1/1	1/1	0/0	1/1	intergenic_	MODIFIER	NONE			-1	MMD2-WIPI2				-1
3	14687749		G	A	1302.61		143	1/1	1/1	1/1	1/1	1/1	0/0	1/1	intergenic_	MODIFIER	NONE			-1	U6-ENSSSCG00000007727				-1
3	38056015		C	T	1094.51		127	1/1	1/1	1/1	1/1	1/1	0/0	1/1	intergenic_	MODIFIER	NONE			-1	U6-U6				-1
3	51502521		T	TG	3166.99		217	1/1	1/1	0/1	0/1	1/1	0/0	0/1	intergenic_	MODIFIER	NONE	n.null_nullinsG		-1	NCK2-FHL2				-1

Head	Note
#CHROM	Chromosome
POS	Position
ID	Identification
REF	Reference seq (Sscrofa 10.2)
ALT	Alternative sequence
QUAL	Quality
FILTER	
DP	Total depth
EFF[*].EFFE	Genetic element
EFF[*].IMP	Functional annotation
EFF[*].FUNCLASS	
EFF[*].CODON	
EFF[*].AA	
EFF[*].AA_LEN	
EFF[*].GENE	
EFF[*].BIOTYPE	
EFF[*].CODING	
EFF[*].TRID	
EFF[*].RANK	
./.	Not called
0/0	Homogeneous to REF
0/1	Heterogeneous to REF
1/1	Homogeneous to ALT

3	1.42E+08	T	G	4161.94	1151	1/1	1/1	1/1	0/1	0/1	0/0	0/1	intergenic	MODIFIER	NONE		-1	ACP1-ENSSSCG00000008661			-1
3	1.42E+08	C	G	7956.76	2096	1/1	1/1	1/1	1/1	1/1	0/0	1/1	intergenic	MODIFIER	NONE		-1	ACP1-ENSSSCG00000008661			-1
4	4068921	T	TG	1661.78	218	0/0	0/1	0/1	0/1	1/1	1/1	1/1	intergenic	MODIFIER	NONE	n.null_nullinsG	-1	ENSSSCG00000005938-5S_rRNA			-1
4	20598904	C	CT	1482.44	258	0/0	0/1	0/0	0/1	0/1	1/1	1/1	intergenic	MODIFIER	NONE	n.null_nullinsT	-1	NOV-MAL2			-1
4	43291944	CAAAAAA	C	3569.67	156	0/0	0/1	0/1	0/1	0/1	1/1	1/1	intergenic	MODIFIER	NONE	n.null_nulldelAAAAA	-1	SDC2-PTDSS1			-1
4	74051994	T	C	1357.71	165	0/0	0/1	0/1	1/1	1/1	1/1	1/1	intergenic	MODIFIER	NONE		-1	ENSSSCG00000006206-ENSSSCG00000023			-1
4	74052001	T	C	1158.98	164	0/0	0/1	0/1	1/1	1/1	1/1	1/1	intergenic	MODIFIER	NONE		-1	ENSSSCG00000006206-ENSSSCG00000023			-1
4	84082675	GA	G	3538.89	265	0/0	0/0	0/1	1/1	1/1	1/1	1/1	intergenic	MODIFIER	NONE		-1	SOX17-MRPL15			-1
4	86371133	A	G	1238.81	164	0/0	0/1	0/1	1/1	1/1	1/1	1/1	intergenic	MODIFIER	NONE		-1	PCMTD1-SNAI2			-1
4	1.3E+08	GA	G	1112.08	164	0/0	0/1	0/1	0/1	0/1	1/1	1/1	intergenic	MODIFIER	NONE		-1	ENSSSCG00000006870-SLC35A3			-1
4	9067293	T	C	1436.02	152	1/1	0/1	0/1	1/1	1/1	0/0	1/1	intron_var	MODIFIER	NONE	c.112+516T>C	-1	OC90	protein_coding	ENSSSCT0	1
4	9067304	A	G	1354.02	149	1/1	0/1	0/1	1/1	1/1	0/0	1/1	intron_var	MODIFIER	NONE	c.112+527A>G	-1	OC90	protein_coding	ENSSSCT0	1
4	24018739	A	G	1974.22	127	1/1	1/1	1/1	0/1	0/1	0/0	./.	intergenic	MODIFIER	NONE		-1	SNORA31-TRPS1			-1
4	33362195	T	TA	2516.62	245	1/1	0/1	1/1	0/1	0/1	0/0	1/1	intergenic	MODIFIER	NONE	n.null_nullinsA	-1	ABRA-ENSSSCG00000006037			-1
4	38937181	CAA	C	2837.04	168	1/1	1/1	1/1	1/1	1/1	0/0	1/1	intron_var	MODIFIER	NONE	c.428-391_428-390de	-1	YWHAZ	protein_coding	ENSSSCT0	3
4	39097076	A	AC	2577.95	157	1/1	1/1	1/1	1/1	1/1	0/0	0/1	intergenic	MODIFIER	NONE	n.null_nullinsC	-1	YWHAZ-PABPC1			-1
4	1.23E+08	T	TAA	1208.17	201	1/1	1/1	0/1	1/1	0/1	0/0	0/1	intergenic	MODIFIER	NONE	n.null_nullinsAA	-1	NTNG1-ENSSSCG00000006853			-1
4	1.42E+08	G	A	2361.39	152	1/1	1/1	1/1	1/1	1/1	0/0	1/1	intergenic	MODIFIER	NONE		-1	ODF2L-ZNHIT6			-1
5	9089370	C	CT	2230.04	216	0/0	0/0	0/0	1/1	1/1	1/1	1/1	intron_var	MODIFIER	NONE	c.3101-81_3101-80ins	-1	MYH9	protein_coding	ENSSSCT0	24
5	74973826	G	GC	1277.73	152	1/1	1/1	1/1	0/1	0/1	0/0	1/1	intron_var	MODIFIER	NONE	c.-128-43159_-128-4	-1	ENSSSCG	protein_coding	ENSSSCT0	1
6	2338761	T	C	1084.1	129	0/0	0/1	0/1	1/1	1/1	1/1	0/1	intergenic	MODIFIER	NONE		-1	C16orf95-FOXL1			-1
6	3432569	C	G	1130.18	137	0/0	0/1	0/1	1/1	1/1	1/1	0/1	intergenic	MODIFIER	NONE		-1	MTHFSD-COX4I1			-1
6	17071811	A	C	1049.52	1905	0/0	0/1	0/1	1/1	1/1	1/1	1/1	intergenic	MODIFIER	NONE		-1	CDH3-RSPRY1			-1
6	29037648	A	G	1211.43	115	0/0	0/1	0/1	1/1	1/1	1/1	1/1	intergenic	MODIFIER	NONE		-1	ENSSSCG00000002834-TOX3			-1
6	31093904	A	G	1081.53	127	0/0	0/1	0/1	1/1	1/1	1/1	1/1	intergenic	MODIFIER	NONE		-1	ZNF423-N4BP1			-1
6	59974516	T	C	1514.54	134	0/0	0/1	0/1	1/1	1/1	1/1	1/1	intergenic	MODIFIER	NONE		-1	ENSSSCG00000003361-C1orf174			-1
6	78665344	GA	G	1779.58	202	0/0	0/0	0/1	0/1	0/1	1/1	1/1	intron_var	MODIFIER	NONE	c.572-1071delT	-1	EYA3	protein_coding	ENSSSCT0	4
6	84569119	CG	C	3720.18	245	0/0	0/0	0/1	0/1	0/1	1/1	1/1	intergenic	MODIFIER	NONE		-1	C1orf94-ENSSSCG00000003624			-1
6	1.41E+08	TA	T	1028.28	233	0/0	0/1	0/1	0/0	0/1	1/1	0/1	intergenic	MODIFIER	NONE		-1	ENSSSCG00000003828-JUN			-1
6	1.38E+08	T	C	1602.12	143	1/1	1/1	1/1	1/1	1/1	0/0	1/1	intergenic	MODIFIER	NONE		-1	ATG4C-U6			-1
6	1.38E+08	C	A	1521.16	143	1/1	1/1	1/1	1/1	1/1	0/0	1/1	intergenic	MODIFIER	NONE		-1	ATG4C-U6			-1
6	1.39E+08	C	T	1150.3	110	1/1	1/1	1/1	1/1	1/1	0/0	1/1	intergenic	MODIFIER	NONE		-1	KANK4-ENSSSCG00000023243			-1
6	1.43E+08	C	T	1529.39	746	1/1	1/1	1/1	./.	1/1	0/0	0/1	intergenic	MODIFIER	NONE		-1	C8A-C1orf168			-1
6	1.43E+08	T	A	2840.78	4248	1/1	1/1	0/1	0/1	0/1	0/0	0/1	intergenic	MODIFIER	NONE		-1	C8A-C1orf168			-1
7	928362	C	A	1608.58	179	1/1	1/1	1/1	1/1	1/1	0/0	0/1	intergenic	MODIFIER	NONE		-1	FOXF2-GMDS			-1
7	2367332	G	A	1205.12	154	1/1	1/1	1/1	1/1	1/1	0/0	1/1	upstream_	MODIFIER	NONE	c.-1G>A	-1	FAM50B	protein_coding	ENSSSCT0	-1
7	2367332	G	A	1205.12	154	1/1	1/1	1/1	1/1	1/1	0/0	1/1	intergenic	MODIFIER	NONE		-1	PXDC1-FAM50B			-1
7	2596504	C	A	1974.76	164	1/1	1/1	1/1	1/1	1/1	0/0	1/1	intergenic	MODIFIER	NONE		-1	ECI2-ENSSSCG00000000999			-1
7	30643829	G	T	1988.1	143	1/1	1/1	1/1	1/1	1/1	0/0	0/1	intergenic	MODIFIER	NONE		-1	FAM83B-ENSSSCG00000001484			-1
7	30836318	T	C	1117.74	143	1/1	1/1	1/1	1/1	1/1	0/0	1/1	intergenic	MODIFIER	NONE		-1	FAM83B-ENSSSCG00000001484			-1
7	31064626	C	T	1176.17	138	1/1	1/1	1/1	1/1	1/1	0/0	0/1	intergenic	MODIFIER	NONE		-1	ENSSSCG00000001484-ENSSSCG00000001			-1
7	32893934	C	T	1130.01	176	1/1	1/1	1/1	1/1	1/1	0/0	0/1	intron_var	MODIFIER	NONE	c.694-15474G>A	-1	ENSSSCG	protein_coding	ENSSSCT0	7
7	33452094	A	G	2162.6	159	1/1	1/1	1/1	1/1	1/1	0/0	0/1	intergenic	MODIFIER	NONE		-1	ENSSSCG00000025172-DST			-1
7	38316792	A	G	1346.47	140	1/1	1/1	1/1	1/1	1/1	0/0	1/1	intergenic	MODIFIER	NONE		-1	ENSSSCG00000001583-ENSSSCG00000001			-1
7	47277517	G	A	1624.89	108	1/1	1/1	1/1	1/1	1/1	0/0	1/1	upstream_	MODIFIER	NONE	c.-428C>T	-1	ENPP4	protein_coding	ENSSSCT0	-1
7	47277517	G	A	1624.89	108	1/1	1/1	1/1	1/1	1/1	0/0	1/1	intergenic	MODIFIER	NONE		-1	ENPP4-RCAN2			-1
8	21945384	T	C	1257.37	140	0/0	0/1	1/1	1/1	1/1	1/1	1/1	intergenic	MODIFIER	NONE		-1	7SK-ENSSSCG00000008763			-1
8	23009229	CG	C	1080.68	241	0/0	0/0	0/0	1/1	1/1	1/1	1/1	intergenic	MODIFIER	NONE		-1	ENSSSCG00000008763-PCDH7			-1
8	26874123	A	C	1342.97	136	0/0	0/1	0/1	1/1	1/1	1/1	1/1	intergenic	MODIFIER	NONE		-1	PCDH7-ENSSSCG00000008767			-1
8	33762665	GT	G	1159.43	216	0/0	1/1	1/1	1/1	1/1	1/1	0/1	intergenic	MODIFIER	NONE		-1	NSUN7-U6			-1
8	39555553	T	TTA	4723.47	227	0/0	0/0	0/0	1/1	1/1	1/1	1/1	intron_var	MODIFIER	NONE	c.2411+1756_2411+1	-1	CORIN	protein_coding	ENSSSCT0	16
8	45200463	CT	C	2816.01	228	0/0	0/1	0/0	1/1	1/1	1/1	1/1	intergenic	MODIFIER	NONE		-1	ENSSSCG00000024269-CPE			-1
8	87464621	G	C	1468.37	178	1/1	1/1	1/1	1/1	1/1	0/0	0/1	intron_var	MODIFIER	NONE	c.397-24591G>C	-1	SLC10A7	protein_coding	ENSSSCT0	4
8	91243682	G	A	1121.08	157	1/1	1/1	1/1	1/1	1/1	0/0	0/1	intergenic	MODIFIER	NONE		-1	IL15-ENSSSCG00000009052			-1
8	91357497	T	C	1793.91	153	1/1	1/1	1/1	1/1	1/1	0/0	0/1	intergenic	MODIFIER	NONE		-1	ENSSSCG00000009052-RNF150			-1
8	91374454	T	A	1591.76	160	1/1	1/1	1/1	1/1	1/1	0/0	0/1	intergenic	MODIFIER	NONE		-1	ENSSSCG00000009052-RNF150			-1
8	92971857	C	T	1650.73	174	1/1	1/1	1/1	1/1	1/1	0/0	0/1	intergenic	MODIFIER	NONE		-1	MGST2-RAB33B			-1
8	1.17E+08	T	C	1702.7	161	1/1	1/1	1/1	1/1	1/1	0/0	0/1	intergenic	MODIFIER	NONE		-1	ENSSSCG00000021844-ARSJ			-1
8	1.22E+08	A	AT	3506.92	224	1/1	1/1	1/1	1/1	1/1	0/0	1/1	intergenic	MODIFIER	NONE	n.null_nullinsT	-1	RPL34-LEF1			-1
8	1.24E+08	CAA	C	2250.3	127	1/1	1/1	1/1	1/1	1/1	0/0	1/1	upstream_	MODIFIER	NONE	c.-3_-3delTT	-1	ENSSSCG	protein_coding	ENSSSCT0	-1
8	1.24E+08	CAA	C	2250.3	127	1/1	1/1	1/1	1/1	1/1	0/0	1/1	intergenic	MODIFIER	NONE	n.null_nulldelAA	-1	ENSSSCG00000020821-ENSSSCG00000023			-1
8	1.3E+08	GCCAA	G	6249	148	1/1	1/1	1/1	1/1	1/1	0/0	1/1	intergenic	MODIFIER	NONE	n.null_nulldelCCAA	-1	ENSSSCG00000030522-ADH4			-1
8	1.31E+08	G	T	7800.54	259	1/1	1/1	1/1	1/1	1/1	0/0	1/1	intron_var	MODIFIER	NONE	c.167-3261C>A	-1	METAP1	protein_coding	ENSSSCT0	2
8	1.31E+08	TC	T	5375.14	222	1/1	1/1	1/1	1/1	1/1	0/0	1/1	intron_var	MODIFIER	NONE	c.81+24560delC	-1	TSPAN5	protein_coding	ENSSSCT0	1
8	1.32E+08	T	C	1077.01	179	1/1	1/1	1/1	1/1	1/1	0/0	1/1	intergenic	MODIFIER	NONE		-1	U6-ENSSSCG00000019832			-1
8	1.38E+08	A	AT	3608.97	249	1/1	1/1	1/1	0/1	0/1	0/0	1/1	intergenic	MODIFIER	NONE	n.null_nullinsT	-1	ENSSSCG00000029197-ENSSSCG00000022			-1
8	1.41E+08	T	C	6968.53	375	1/1	1/1	1/1	1/1	1/1	0/0	0/1	intron_var	MODIFIER	NONE	c.6358-1846A>G	-1	PTPN13	protein_coding	ENSSSCT0	40

9	4683739	A	G	1065.02	152	0/0	1/1	0/1	1/1	1/1	1/1	1/1	intergenic_	MODIFIER	NONE		-1	ENSSSCG00000014673-TRIM6			-1
9	55718387	A	G	1027.08	160	0/0	0/1	0/1	1/1	1/1	1/1	0/1	intergenic_	MODIFIER	NONE		-1	ENSSSCG00000015140-ENSSSCG00000015			-1
9	55737157	G	T	1022.89	124	0/0	0/1	0/1	1/1	1/1	1/1	1/1	intergenic_	MODIFIER	NONE		-1	ENSSSCG00000015140-ENSSSCG00000015			-1
9	56254834	T	A	2035.29	138	0/0	0/1	0/1	1/1	1/1	1/1	1/1	intergenic_	MODIFIER	NONE		-1	ENSSSCG00000015162-ENSSSCG00000025			-1
9	58075484	G	A	1752.15	167	0/0	0/1	0/1	1/1	1/1	1/1	0/1	intergenic_	MODIFIER	NONE		-1	TMEM218-ENSSSCG00000015211			-1
9	1.21E+08	C	T	1271.49	387	0/0	0/0	0/0	0/1	0/0	1/1	0/1	intergenic_	MODIFIER	NONE		-1	ENSSSCG00000015458-ENSSSCG00000029			-1
9	13168406	T	A	1163.88	108	1/1	1/1	1/1	1/1	1/1	0/0	0/1	intergenic_	MODIFIER	NONE		-1	PAK1-AQP11			-1
9	29131911	T	C	1176.04	149	1/1	1/1	1/1	1/1	1/1	0/0	0/0	intron_var	MODIFIER	NONE	c.-26-1780T>C	-1	ENSSSCG(	protein_coding	ENSSSCT0	2
9	30818604	A	C	1332.26	114	1/1	1/1	1/1	1/1	1/1	0/0	1/1	intergenic_	MODIFIER	NONE		-1	CWC15-ENSSSCG00000026272			-1
9	31130725	A	G	1917.95	155	1/1	1/1	1/1	1/1	1/1	0/0	1/1	intergenic_	MODIFIER	NONE		-1	SESN3-FAM76B			-1
9	31755919	C	T	1068.21	106	1/1	1/1	1/1	1/1	1/1	0/0	1/1	intergenic_	MODIFIER	NONE		-1	FAM76B-ENSSSCG00000014969			-1
9	31755935	T	C	1313.43	125	1/1	1/1	1/1	1/1	1/1	0/0	1/1	intergenic_	MODIFIER	NONE		-1	FAM76B-ENSSSCG00000014969			-1
9	42511529	A	T	1967.82	131	1/1	1/1	1/1	1/1	0/1	0/0	1/1	intergenic_	MODIFIER	NONE		-1	C11orf87-U1			-1
9	62639271	TA	T	1317.69	202	1/1	1/1	1/1	0/1	0/1	0/0	0/0	intergenic_	MODIFIER	NONE		-1	BARX2-SNORD112			-1
9	69870502	G	A	1351.32	161	1/1	1/1	1/1	1/1	1/1	0/0	0/1	intron_var	MODIFIER	NONE	c.454-982G>A	-1	ENSSSCG(	protein_coding	ENSSSCT0	5
9	1.29E+08	TGG	T	3976.49	254	1/1	0/1	1/1	0/1	0/1	0/0	1/1	intron_var	MODIFIER	NONE	c.3702+645_3702+64	-1	TNN	protein_coding	ENSSSCT0	32
9	1.32E+08	G	A	1324.62	144	1/1	1/1	1/1	1/1	1/1	0/0	1/1	intron_var	MODIFIER	NONE	c.-72+17526G>A	-1	RASAL2	protein_coding	ENSSSCT0	3
9	1.38E+08	C	A	1258.36	107	1/1	1/1	1/1	1/1	1/1	0/0	1/1	intergenic_	MODIFIER	NONE		-1	C1orf21-EDEM3			-1
10	64031898	A	G	1811.04	138	0/0	0/1	0/1	1/1	1/1	1/1	1/1	intergenic_	MODIFIER	NONE		-1	GJD4-CCDC3			-1
10	68794575	TA	T	1852.33	242	0/0	0/0	0/1	0/1	0/1	1/1	0/1	intergenic_	MODIFIER	NONE		-1	U6-ENSSSCG00000029177			-1
10	70261400	A	G	1621.14	168	0/0	0/1	0/1	0/1	0/1	1/1	0/1	intergenic_	MODIFIER	NONE		-1	U6-ENSSSCG00000018589			-1
11	14944055	A	G	1973	162	0/0	0/1	0/1	0/1	0/1	1/1	0/1	intergenic_	MODIFIER	NONE		-1	COG6-FOXO1			-1
11	82268654	AGTTGAG	A	1244.06	239	0/0	0/0	0/0	0/1	0/1	1/1	0/1	intergenic_	MODIFIER	NONE	n.null_nulldelGTTGAG	-1	FAM155A-ENSSSCG00000009539			-1
11	1887438	AAAAT	A	3256.31	223	1/1	1/1	1/1	0/1	0/1	0/0	0/1	intron_var	MODIFIER	NONE	c.446-14128_446-141	-1	TNFRSF19	protein_coding	ENSSSCT0	5
11	2234773	T	C	1143.95	132	1/1	0/0	0/1	1/1	1/1	0/0	1/1	intron_var	MODIFIER	NONE	c.55+21048A>G	-1	ENSSSCG(	protein_coding	ENSSSCT0	1
11	35390104	G	A	1331.53	130	1/1	1/1	1/1	1/1	1/1	0/0	0/1	intergenic_	MODIFIER	NONE		-1	ENSSSCG00000009449-SNORA31			-1
12	7432755	G	A	1239.5	134	1/1	1/1	1/1	1/1	1/1	0/0	1/1	intergenic_	MODIFIER	NONE		-1	RPL38-SDK2			-1
12	7432764	G	A	1367.47	138	1/1	1/1	1/1	1/1	1/1	0/0	1/1	intergenic_	MODIFIER	NONE		-1	RPL38-SDK2			-1
12	8693471	C	G	1468.74	153	1/1	1/1	1/1	1/1	1/1	0/0	1/1	intergenic_	MODIFIER	NONE		-1	SLC39A11-SOX9			-1
12	22828565	T	C	1095.34	137	1/1	1/1	1/1	1/1	1/1	0/0	0/0	intron_var	MODIFIER	NONE	c.707-168A>G	-1	ENSSSCG(	protein_coding	ENSSSCT0	5
12	22835615	T	C	1668.93	129	1/1	1/1	1/1	1/1	1/1	0/0	1/1	upstream_	MODIFIER	NONE	c.-1A>G	-1	ENSSSCG(	protein_coding	ENSSSCT0	-1
12	22835615	T	C	1668.93	129	1/1	1/1	1/1	1/1	1/1	0/0	1/1	intergenic_	MODIFIER	NONE		-1	ENSSSCG00000017490-ENSSSCG00000017			-1
12	48947845	A	T	3669.6	5516	1/1	0/1	0/1	0/1	0/1	0/0	0/1	intergenic_	MODIFIER	NONE		-1	U6-ABR			-1
13	403773	C	CA	3243.74	202	0/0	0/0	0/1	1/1	1/1	1/1	1/1	intron_var	MODIFIER	NONE	c.180+44121_180+44	-1	ENSSSCG(	protein_coding	ENSSSCT0	1
13	1.34E+08	G	A	1307.6	143	0/0	0/1	0/1	1/1	1/1	1/1	0/1	intergenic_	MODIFIER	NONE		-1	RFC4-MASP1			-1
13	1.87E+08	G	GA	1764.93	238	0/0	0/1	0/1	1/1	1/1	1/1	1/1	intergenic_	MODIFIER	NONE	n.null_nullinsA	-1	ROBO1-ENSSSCG00000012002			-1
13	2.15E+08	C	CGGGAGA	10414.89	225	0/0	0/1	0/1	1/1	1/1	1/1	0/1	intergenic_	MODIFIER	NONE	n.null_nullinsGGGAG/	-1	ENSSSCG00000012078-ENSSSCG00000012			-1
13	7733142	C	G	1113.12	147	1/1	1/1	1/1	0/1	0/1	0/0	0/1	intron_var	MODIFIER	NONE	c.825+4020C>G	-1	EFHB	protein_coding	ENSSSCT0	1
13	14327965	A	G	4440.15	283	1/1	1/1	1/1	0/1	0/1	0/0	0/1	intergenic_	MODIFIER	NONE		-1	OXSM-LRRC3B			-1
13	1.14E+08	TTA	T	2637.19	178	1/1	0/1	1/1	0/1	1/1	0/0	1/1	intergenic_	MODIFIER	NONE	n.null_nulldelTA	-1	BCHE-ZBBX			-1
14	11250816	G	T	1477.15	177	0/0	1/1	1/1	0/1	0/1	1/1	1/1	intergenic_	MODIFIER	NONE		-1	ENSSSCG00000009655-PPP2R2A			-1
14	12224629	C	CAT	1782.1	244	0/0	0/1	0/1	0/1	0/1	1/1	0/1	intron_var	MODIFIER	NONE	c.436-3030_436-3029	-1	TRIM35	protein_coding	ENSSSCT0	1
14	62454044	GT	G	2509.49	237	0/0	0/1	0/1	1/1	1/1	1/1	0/1	intron_var	MODIFIER	NONE	c.3350+733delA	-1	SIPA1L2	protein_coding	ENSSSCT0	11
14	10921916	A	G	1106.65	134	1/1	1/1	1/1	1/1	1/1	0/0	0/1	intergenic_	MODIFIER	NONE		-1	CDCA2-ENSSSCG00000022148			-1
14	11232079	C	T	1365.75	132	1/1	1/1	1/1	1/1	1/1	0/0	0/1	intergenic_	MODIFIER	NONE		-1	ENSSSCG00000009655-PPP2R2A			-1
14	11232125	C	A	1520.67	111	1/1	1/1	1/1	1/1	1/1	0/0	0/1	intergenic_	MODIFIER	NONE		-1	ENSSSCG00000009655-PPP2R2A			-1
14	11232127	T	C	1506.6	112	1/1	1/1	1/1	1/1	1/1	0/0	0/1	intergenic_	MODIFIER	NONE		-1	ENSSSCG00000009655-PPP2R2A			-1
14	11232129	A	G	1732.52	118	1/1	1/1	1/1	1/1	1/1	0/0	0/1	intergenic_	MODIFIER	NONE		-1	ENSSSCG00000009655-PPP2R2A			-1
14	50886236	AC	A	3668.45	187	1/1	1/1	1/1	1/1	1/1	0/0	0/1	intron_var	MODIFIER	NONE	c.426+1535delG	-1	MORC2	protein_coding	ENSSSCT0	6
14	58561330	T	C	5117.15	292	1/1	1/1	1/1	0/1	0/1	0/0	0/1	intron_var	MODIFIER	NONE	c.1512+503A>G	-1	MTR	protein_coding	ENSSSCT0	16
14	58859778	G	T	8164.15	388	1/1	1/1	1/1	0/1	0/1	0/0	0/1	intron_var	MODIFIER	NONE	c.45+3513C>A	-1	LGALS8	protein_coding	ENSSSCT0	1
14	59983496	A	G	5287.15	230	1/1	1/1	1/1	0/1	0/1	0/0	0/1	intergenic_	MODIFIER	NONE		-1	TBCE-GGPS1			-1
14	61203599	CT	C	3265.99	214	1/1	1/1	1/1	1/1	1/1	0/0	1/1	intergenic_	MODIFIER	NONE		-1	SLC35F3-KCNK1			-1
14	1.16E+08	C	CA	1060.08	214	1/1	1/1	1/1	1/1	0/1	0/0	1/1	intron_var	MODIFIER	NONE	c.688+3065_688+306	-1	ENSSSCG(	protein_coding	ENSSSCT0	9
14	1.16E+08	G	C	1131.99	122	1/1	1/1	1/1	1/1	1/1	0/0	1/1	intron_var	MODIFIER	NONE	c.688+1970C>G	-1	ENSSSCG(	protein_coding	ENSSSCT0	9
14	1.16E+08	G	C	1420.9	145	1/1	1/1	1/1	1/1	1/1	0/0	0/1	intron_var	MODIFIER	NONE	c.688+1912C>G	-1	ENSSSCG(	protein_coding	ENSSSCT0	9
14	1.16E+08	C	T	1958.88	154	1/1	1/1	1/1	1/1	1/1	0/0	1/1	intron_var	MODIFIER	NONE	c.688+1890G>A	-1	ENSSSCG(	protein_coding	ENSSSCT0	9
14	1.19E+08	A	C	1314.17	185	1/1	1/1	1/1	1/1	1/1	0/0	1/1	intergenic_	MODIFIER	NONE		-1	LOXL4-ENSSSCG00000010533			-1
14	1.31E+08	G	A	1212.32	124	1/1	1/1	1/1	1/1	1/1	0/0	1/1	intergenic_	MODIFIER	NONE		-1	SNORA62-XPNPEP1			-1
14	1.42E+08	A	C	1910.13	118	1/1	1/1	1/1	0/1	1/1	0/0	0/1	intergenic_	MODIFIER	NONE		-1	ENSSSCG00000010692-FGFR2			-1
14	1.42E+08	A	C	1751.03	113	1/1	0/1	0/1	0/1	1/1	0/0	1/1	intergenic_	MODIFIER	NONE		-1	ENSSSCG00000010692-FGFR2			-1
15	19740207	C	A	1451.84	147	0/0	0/1	0/1	1/1	1/1	1/1	1/1	intergenic_	MODIFIER	NONE		-1	ENSSSCG00000029118-ENSSSCG00000015			-1
15	24436358	T	C	1285.4	173	0/0	0/1	0/1	1/1	1/1	1/1	1/1	intergenic_	MODIFIER	NONE		-1	U6-ENSSSCG00000026504			-1
15	29972309	G	A	1001.36	145	0/0	0/1	0/1	1/1	1/1	1/1	0/1	intergenic_	MODIFIER	NONE		-1	GYPC-ENSSSCG00000023800			-1
15	34676931	C	T	1022.27	153	0/0	0/1	0/1	1/1	1/1	1/1	0/1	upstream_	MODIFIER	NONE	c.-76C>T	-1	ENSSSCG(	protein_coding	ENSSSCT0	-1
15	34676931	C	T	1022.27	153	0/0	0/1	0/1	1/1	1/1	1/1	0/1	intergenic_	MODIFIER	NONE		-1	TSN-ENSSSCG00000015730			-1
15	38157850	T	TA	1519.66	225	0/0	0/1	0/1	1/1	1/1	1/1	1/1	intergenic_	MODIFIER	NONE	n.null_nullinsA	-1	ENSSSCG00000015747-ENSSSCG00000029			-1

15	40325994	A	C	1181.59	141	0/0	0/1	0/1	1/1	1/1	1/1	0/1	intergenic_	MODIFIER	NONE		-1	ENSSSCG00000015748-U6			-1
15	65506863	G	A	1070.23	133	0/0	0/1	0/1	1/1	1/1	1/1	1/1	intergenic_	MODIFIER	NONE		-1	SNORA19-HS6ST1			-1
15	65506911	C	T	1551.78	156	0/0	0/1	0/1	1/1	1/1	1/1	1/1	intergenic_	MODIFIER	NONE		-1	SNORA19-HS6ST1			-1
15	1.09E+08	A	C	1425.75	160	0/0	0/1	0/1	1/1	1/1	1/1	1/1	intergenic_	MODIFIER	NONE		-1	TMEFF2-STK17B			-1
15	1.36E+08	G	T	1526.59	126	0/0	0/1	0/1	1/1	1/1	1/1	1/1	intergenic_	MODIFIER	NONE		-1	U6-SNORA31			-1
15	1.41E+08	C	CA	1924.64	229	0/0	0/1	0/1	0/1	0/1	1/1	1/1	intergenic_	MODIFIER	NONE	n.null_nullinsA	-1	U6-ENSSSCG00000030288			-1
15	1.48E+08	A	C	1090.49	279	1/1	0/1	1/1	1/1	1/1	0/0	0/1	intergenic_	MODIFIER	NONE		-1	ENSSSCG00000016302-ENSSSCG00000030			-1
15	1.52E+08	T	G	2098.44	178	1/1	1/1	1/1	0/1	0/1	0/0	0/1	intergenic_	MODIFIER	NONE		-1	ENSSSCG00000016328-ENSSSCG00000016			-1
15	1.52E+08	TA	T	1704.25	190	1/1	1/1	1/1	0/1	0/1	0/0	0/0	upstream_	MODIFIER	NONE	c.-1delA	-1	ENSSSCG(	protein_coding	ENSSSCT0	-1
15	1.52E+08	TA	T	1704.25	190	1/1	1/1	1/1	0/1	0/1	0/0	0/0	intergenic_	MODIFIER	NONE		-1	KLHL30-ENSSSCG00000016337			-1
15	1.54E+08	G	A	1353.36	153	1/1	1/1	1/1	0/1	0/1	0/0	0/1	intergenic_	MODIFIER	NONE		-1	ENSSSCG00000023644-ENSSSCG00000029			-1
15	1.54E+08	C	A	1180.32	134	1/1	1/1	1/1	0/1	0/1	0/0	0/1	intergenic_	MODIFIER	NONE		-1	ENSSSCG00000023644-ENSSSCG00000029			-1
16	8032570	GA	G	2914.9	230	0/0	0/1	0/0	1/1	1/1	1/1	0/1	intergenic_	MODIFIER	NONE		-1	7SK-CDH18			-1
16	20790534	A	G	1658.17	148	0/0	0/1	0/1	1/1	1/1	1/1	0/1	intergenic_	MODIFIER	NONE		-1	AMACR-C1QTNF3			-1
16	3389119	C	G	2412.15	153	1/1	1/1	1/1	0/1	0/1	0/0	0/1	intergenic_	MODIFIER	NONE		-1	U6-ENSSSCG00000029792			-1
16	4280860	C	T	5137.61	307	1/1	1/1	1/1	0/1	0/1	0/0	0/1	intergenic_	MODIFIER	NONE		-1	ENSSSCG00000016781-FAM105A			-1
16	23627177	G	A	1113.76	128	1/1	1/1	1/1	1/1	1/1	0/0	0/0	intergenic_	MODIFIER	NONE		-1	C5orf42-NUP155			-1
16	51146564	C	T	1708.51	119	1/1	1/1	1/1	0/1	0/1	0/0	1/1	intergenic_	MODIFIER	NONE		-1	ENSSSCG00000024880-CCNB1			-1
16	51214619	A	T	1043.51	136	1/1	./.	1/1	0/1	0/1	0/0	1/1	intron_vari	MODIFIER	NONE	c.84-4007T>A	-1	SLC30A5	protein_coding	ENSSSCT0	1
16	51214627	C	T	1148.5	134	1/1	1/1	1/1	0/1	0/1	0/0	1/1	intron_vari	MODIFIER	NONE	c.84-4015G>A	-1	SLC30A5	protein_coding	ENSSSCT0	1
16	51368450	T	A	1304.22	150	1/1	1/1	1/1	0/1	0/0	0/0	1/1	intron_vari	MODIFIER	NONE	c.-40-3851T>A	-1	ENSSSCG(	protein_coding	ENSSSCT0	1
16	51534787	G	A	2600.53	161	1/1	1/1	1/1	0/1	0/1	0/0	1/1	intergenic_	MODIFIER	NONE		-1	ENSSSCG00000027784-ENSSSCG00000021			-1
16	76268357	C	T	1498.96	152	1/1	1/1	1/1	0/1	0/1	0/0	0/1	intergenic_	MODIFIER	NONE		-1	SNORA18-U6			-1
17	36984058	C	CT	1772.88	238	0/0	0/0	0/0	0/1	1/1	1/1	1/1	intergenic_	MODIFIER	NONE	n.null_nullinsT	-1	OXT-MRPS26			-1
17	40342407	C	T	1218.62	126	0/0	0/1	0/1	1/1	1/1	1/1	1/1	upstream_	MODIFIER	NONE	c.-1C>T	-1	ENSSSCG(	protein_coding	ENSSSCT0	-1
17	40342407	C	T	1218.62	126	0/0	0/1	0/1	1/1	1/1	1/1	1/1	intron_vari	MODIFIER	NONE	c.-172+1139C>T	-1	DUSP15	protein_coding	ENSSSCT0	3
17	40342423	A	T	1394.48	134	0/0	0/1	0/1	1/1	1/1	1/1	1/1	upstream_	MODIFIER	NONE	c.-1A>T	-1	ENSSSCG(	protein_coding	ENSSSCT0	-1
17	40342423	A	T	1394.48	134	0/0	0/1	0/1	1/1	1/1	1/1	1/1	intron_vari	MODIFIER	NONE	c.-172+1155A>T	-1	DUSP15	protein_coding	ENSSSCT0	3
17	40342441	T	G	1631.21	147	0/0	0/1	0/1	1/1	1/1	1/1	1/1	upstream_	MODIFIER	NONE	c.-1T>G	-1	ENSSSCG(	protein_coding	ENSSSCT0	-1
17	40342441	T	G	1631.21	147	0/0	0/1	0/1	1/1	1/1	1/1	1/1	intron_vari	MODIFIER	NONE	c.-172+1173T>G	-1	DUSP15	protein_coding	ENSSSCT0	3
17	43895202	T	A	1201.82	145	0/0	0/1	0/1	1/1	1/1	1/1	1/1	intron_vari	MODIFIER	NONE	c.24+870A>T	-1	ENSSSCG(	protein_coding	ENSSSCT0	1
17	21668562	C	T	2394.78	162	1/1	1/1	1/1	1/1	1/1	0/0	1/1	upstream_	MODIFIER	NONE	c.-90C>T	-1	ENSSSCG(	protein_coding	ENSSSCT0	-1
17	21668562	C	T	2394.78	162	1/1	1/1	1/1	1/1	1/1	0/0	1/1	intergenic_	MODIFIER	NONE		-1	ANKEF1-ENSSSCG00000026985			-1
17	23826537	T	A	1078.73	133	1/1	1/1	1/1	1/1	1/1	0/0	1/1	intergenic_	MODIFIER	NONE		-1	ENSSSCG00000007069-SPTLC3			-1
17	23835070	T	G	1434.17	154	1/1	1/1	1/1	1/1	1/1	0/0	1/1	intergenic_	MODIFIER	NONE		-1	ENSSSCG00000007069-SPTLC3			-1
17	31389795	C	G	1023.35	142	1/1	1/1	1/1	1/1	1/1	0/0	1/1	intergenic_	MODIFIER	NONE		-1	ENSSSCG00000028421-U6			-1
17	64082231	C	CCA	1407.88	248	1/1	0/1	0/1	0/1	0/1	0/0	0/1	upstream_	MODIFIER	NONE	c.-8_-8insTG	-1	AURKA	protein_coding	ENSSSCT0	-1
17	64082231	C	CCA	1407.88	248	1/1	0/1	0/1	0/1	0/1	0/0	0/1	upstream_	MODIFIER	NONE	c.-165_-165insCA	-1	CSTF1	protein_coding	ENSSSCT0	-1
17	64082231	C	CCA	1407.88	248	1/1	0/1	0/1	0/1	0/1	0/0	0/1	intragenic_	MODIFIER	NONE	n.null_nullinsTG	-1	AURKA			-1
17	68238296	T	C	2282.55	159	1/1	1/1	1/1	1/1	1/1	0/0	0/1	intergenic_	MODIFIER	NONE		-1	ENSSSCG00000026743-ENSSSCG00000007			-1
18	40749240	G	GA	1047.25	198	0/0	0/1	0/1	1/1	1/1	1/1	0/1	intron_vari	MODIFIER	NONE	c.-141+11760_-141+:	-1	ELMO1	protein_coding	ENSSSCT0	14
18	46377954	A	G	1019.53	97	0/0	0/1	0/1	1/1	1/1	1/1	1/1	intergenic_	MODIFIER	NONE		-1	PAC1-GHRHR			-1
18	55245294	T	C	1405.56	122	0/0	0/1	1/1	0/0	0/1	1/1	1/1	intron_vari	MODIFIER	NONE	c.273+8194A>G	-1	CCM2	protein_coding	ENSSSCT0	3
18	52031687	T	C	3553.03	145	1/1	1/1	1/1	0/1	1/1	0/0	1/1	upstream_	MODIFIER	NONE	c.-74T>C	-1	CYCS	protein_coding	ENSSSCT0	-1
18	52031687	T	C	3553.03	145	1/1	1/1	1/1	0/1	1/1	0/0	1/1	intergenic_	MODIFIER	NONE		-1	ssc-mir-148a-CYCS			-1
18	53769580	TG	T	1178.98	202	1/1	1/1	1/1	0/1	1/1	0/0	0/1	intron_vari	MODIFIER	NONE	c.201+530delG	-1	TNS3	protein_coding	ENSSSCT0	7
18	56318142	A	AC	1116.05	247	1/1	1/1	1/1	1/1	1/1	0/0	0/1	intergenic_	MODIFIER	NONE	n.null_nullinsC	-1	5S_rRNA-HECW1			-1
18	58731545	T	C	1047.26	138	1/1	1/1	1/1	1/1	1/1	0/0	0/1	intergenic_	MODIFIER	NONE		-1	ENSSSCG00000016765-ENSSSCG00000016			-1
X	6727207	TGC	T	3333.81	261	0/0	0/0	0/0	0/1	0/1	1/1	1/1	intron_vari	MODIFIER	NONE	c.317+3568_317+356	-1	SHROOM2	protein_coding	ENSSSCT0	2
X	7195511	T	G	2281.44	201	0/0	0/0	0/0	0/1	0/1	1/1	1/1	intron_vari	MODIFIER	NONE	n.634+28971T>G	-1	WWC3	processed_transcript	ENSSSCT0	1
X	16072712	G	A	2807.39	231	0/0	0/0	0/0	0/1	0/1	1/1	1/1	intron_vari	MODIFIER	NONE	c.-166+29841G>A	-1	CDKL5	protein_coding	ENSSSCT0	1
X	16832884	G	T	2050.16	215	0/0	0/0	0/0	0/1	0/1	0/0	1/1	intergenic_	MODIFIER	NONE		-1	GPR64-CH242-78D20.1			-1
X	41124308	T	C	1782.89	342	0/0	0/0	0/0	0/1	0/1	1/1	0/1	intergenic_	MODIFIER	NONE		-1	ENSSSCG00000028209-DDX3X			-1
X	42697359	C	G	2041.24	207	0/0	0/0	0/0	0/1	0/1	1/1	0/1	intergenic_	MODIFIER	NONE		-1	CH242-132C3.1-MAOA			-1
X	43258366	G	A	2440.15	278	0/0	0/0	0/0	0/1	0/1	1/1	0/1	intergenic_	MODIFIER	NONE		-1	MAOA-MAOB			-1
X	1.26E+08	A	G	3689.76	221	0/0	0/1	0/0	0/1	0/1	1/1	1/1	intron_vari	MODIFIER	NONE	c.1163+5756T>C	-1	GPC3	protein_coding	ENSSSCT0	4
X	1.29E+08	T	A	2958.38	334	0/0	0/0	0/0	0/1	0/1	1/1	1/1	intergenic_	MODIFIER	NONE		-1	ZIC3-CH242-204G11.1			-1
X	1.29E+08	T	C	1319.7	177	0/0	0/0	0/0	0/1	0/1	1/1	1/1	intergenic_	MODIFIER	NONE		-1	ZIC3-CH242-204G11.1			-1
X	1.31E+08	C	T	2758.79	236	0/0	0/1	0/1	0/1	0/1	1/1	1/1	intron_vari	MODIFIER	NONE	c.435+4062G>A	-1	ATP11C	protein_coding	ENSSSCT0	5
X	13939179	A	G	4981.55	203	1/1	1/1	1/1	1/1	1/1	0/0	1/1	intergenic_	MODIFIER	NONE		-1	CH242-19L11.2-CTPS2			-1
X	39984625	G	A	2897.52	302	1/1	1/1	1/1	0/1	0/1	0/0	0/0	intergenic_	MODIFIER	NONE		-1	BCOR-U6			-1
X	41079471	G	A	3869.42	214	1/1	0/1	0/1	0/1	0/1	0/0	0/1	intergenic_	MODIFIER	NONE		-1	CH242-15C8.2-ENSSSCG00000028209			-1
X	41102232	G	T	3260.2	177	1/1	1/1	1/1	0/1	0/1	0/0	0/1	upstream_	MODIFIER	NONE	n.-1C>A	-1	ENSSSCG(	miRNA	ENSSSCT0	-1
X	41102232	G	T	3260.2	177	1/1	1/1	1/1	0/1	0/1	0/0	0/1	intergenic_	MODIFIER	NONE		-1	ENSSSCG00000028209-DDX3X			-1
X	41508271	C	A	3953.15	226	1/1	1/1	1/1	0/1	0/1	0/0	0/1	intron_vari	MODIFIER	NONE	c.832-2414G>T	-1	CASK	protein_coding	ENSSSCT0	8
X	41799395	G	A	1054.68	200	1/1	1/1	1/1	0/1	0/1	0/0	1/1	intergenic_	MODIFIER	NONE		-1	CASK-U6			-1
X	44200178	T	A	2969.2	206	1/1	1/1	1/1	0/1	0/1	0/0	0/1	intergenic_	MODIFIER	NONE		-1	FUNDC1-5S_rRNA			-1

X	44229447	T	C	3585.55	137	1/1	0/1	1/1	1/1	0/1	0/0	1/1	intergenic_	MODIFIER	NONE		-1	FUNDC1-5S_rRNA		-1
X	44407361	T	C	1746.24	207	1/1	1/1	1/1	0/1	0/1	0/0	0/1	intergenic_	MODIFIER	NONE		-1	CH242-517A18.1-DUSP21		-1
X	44769158	C	T	2943.35	169	1/1	1/1	1/1	0/1	0/1	0/0	0/1	intron_var	MODIFIER	NONE	c.174-2066C>T	-1	CXorf36	protein_coding	ENSSSCT0 1
X	45055606	G	A	3180.5	228	1/1	1/1	1/1	0/1	0/1	0/0	0/1	intergenic_	MODIFIER	NONE		-1	CH242-210O3.1-ssc-mir-221		-1
X	1.05E+08	G	A	2693.18	189	1/1	1/1	1/1	0/1	0/1	0/0	0/1	intergenic_	MODIFIER	NONE		-1	NXT2-KCNE1L		-1
X	1.13E+08	G	A	3693.34	606	1/1	1/1	1/1	0/1	0/1	0/0	0/1	intergenic_	MODIFIER	NONE		-1	ENSSSCG00000022359-UPF3B		-1
X	1.15E+08	C	G	2156.2	144	1/1	1/1	1/1	0/1	0/1	0/0	0/1	intergenic_	MODIFIER	NONE		-1	ENSSSCG00000012636-ENSSSCG00000012		-1
X	1.15E+08	C	A	2927.37	181	1/1	1/1	1/1	1/1	1/1	0/0	1/1	intergenic_	MODIFIER	NONE		-1	ENSSSCG00000012637-GRIA3		-1
X	1.15E+08	C	G	2976.45	180	1/1	1/1	1/1	1/1	1/1	0/0	1/1	intergenic_	MODIFIER	NONE		-1	ENSSSCG00000012637-GRIA3		-1
