# Supplementary material for: No excessive mutations in transcription activator-like effector nuclease-mediated α-1,3-galactosyltransferase knockout Yucatan miniature pigs
Source: Asian-Australas J Anim Sci. 2019 Aug 23;33(2):360–72. doi: 10.5713/ajas.19.0480 (PMC6946973; doi:10.5713/ajas.19.0480)
Supplement: Supplementary file 6 [file ajas-19-0480-suppl6.pdf]

Supplementary Table 6. Variant calls of wild type strain control WT3

#CHROM	POS	ID	REF	ALT	QUAL	FILTER	DP	Donor	CJ1	CB1	CB3	WT1	WT2	WT3	EFF[*].EFFECT	EFF[*].IMPACT	EFF[*].FUNCLASS	EFF[*].CODON	EFF[*].AA	EFF[*].AA_LEN	EFF[*].GENE	EFF[*].BIOTYPE	EFF[*].CODING	EFF[*].TRID	EFF[*].RANK
1	9896270		G	C	1115.83		1144	0/0	0/1	0/1	0/1	0/1	0/1	1/1	intron_vari	MODIFIER	NONE	c.377-3078C>G		-1	FNDC1	protein_coding		ENSSSCT0	3
1	1.09E+08		C	CA	1282.4		229	0/0	0/0	0/0	1/1	0/1	1/1	1/1	upstream_	MODIFIER	NONE	n.-1_-1insA		-1	SCARNA17	snoRNA		ENSSSCT0	-1
1	1.09E+08		C	CA	1282.4		229	0/0	0/0	0/0	1/1	0/1	1/1	1/1	upstream_	MODIFIER	NONE	n.-1_-1insA		-1	SCARNA18	snoRNA		ENSSSCT0	-1
1	1.09E+08		C	CA	1282.4		229	0/0	0/0	0/0	1/1	0/1	1/1	1/1	intron_vari	MODIFIER	NONE	c.16+1468_16+1469i		-1	ACAA2	protein_coding		ENSSSCT0	1
1	1.82E+08		T	C	4023.92		210	0/0	0/1	0/1	1/1	1/1	1/1	1/1	intergenic_	MODIFIER	NONE			-1	U2-DIS3L				-1
1	1.9E+08		A	G	1167.65		122	0/0	1/1	0/1	1/1	1/1	1/1	1/1	intergenic_	MODIFIER	NONE			-1	5S_rRNA-U6				-1
1	25316240		A	G	1531.7		149	1/1	1/1	1/1	0/1	0/1	0/1	0/0	intron_vari	MODIFIER	NONE	c.82+17399A>G		-1	GPR126	protein_coding		ENSSSCT0	2
1	25316252		A	G	1288.24		139	1/1	1/1	1/1	0/1	0/1	0/1	0/0	intron_vari	MODIFIER	NONE	c.82+17411A>G		-1	GPR126	protein_coding		ENSSSCT0	2
1	26094212		GT	G	2761.52		245	1/1	1/1	1/1	1/1	1/1	0/1	0/0	intergenic_	MODIFIER	NONE			-1	NMBR-ENSSSCG00000019021				-1
1	34180135		T	C	4928.15		313	1/1	1/1	1/1	0/1	0/1	0/1	0/0	intergenic_	MODIFIER	NONE			-1	ENSSSCG00000023999-RPS12				-1
1	34959460		G	GT	2439.16		265	1/1	0/1	1/1	1/1	1/1	0/1	0/0	intergenic_	MODIFIER	NONE	n.null_nullinsT		-1	MOXD1-CTGF				-1
1	36919425		CA	C	4336.64		222	1/1	1/1	1/1	0/0	0/0	0/1	0/0	intergenic_	MODIFIER	NONE			-1	L3MBTL3-TMEM244				-1
1	38168772		A	AAAT	6612.76		239	1/1	1/1	1/1	0/1	0/1	0/1	0/0	intergenic_	MODIFIER	NONE	n.null_nullinsAAT		-1	ENSSSCG00000029684-PTPRK				-1
1	39516877		C	CA	3080.19		243	1/1	1/1	1/1	0/0	0/0	1/1	0/0	intergenic_	MODIFIER	NONE	n.null_nullinsA		-1	KIAA0408-ENSSSCG00000004216				-1
1	56505569		AT	A	2632.13		212	1/1	1/1	1/1	0/0	0/1	0/1	0/0	intron_vari	MODIFIER	NONE	c.2398-1185delT		-1	COL19A1	protein_coding		ENSSSCT0	36
1	65224934		G	GTCTC	1308.19		138	1/1	0/1	0/1	0/1	0/1	1/1	0/0	intergenic_	MODIFIER	NONE	n.null_nullinsTCTC		-1	BACH2-MAP3K7				-1
1	78454772		A	G	1452.57		135	1/1	1/1	1/1	0/1	0/1	1/1	0/0	intergenic_	MODIFIER	NONE			-1	GRIK2-HACE1				-1
1	78454780		G	C	1445.63		133	1/1	1/1	1/1	0/1	0/1	1/1	0/0	intergenic_	MODIFIER	NONE			-1	GRIK2-HACE1				-1
1	78454817		T	C	1544.52		141	1/1	1/1	1/1	0/1	0/1	1/1	0/0	intergenic_	MODIFIER	NONE			-1	GRIK2-HACE1				-1
1	79645931		TTA	T	1517.32		250	1/1	1/1	1/1	0/1	0/1	1/1	0/0	intergenic_	MODIFIER	NONE	n.null_nulldelTA		-1	GRIK2-HACE1				-1
1	86850348		G	T	1046.14		126	1/1	1/1	1/1	1/1	0/1	1/1	0/0	intron_vari	MODIFIER	NONE	c.177+4648C>A		-1	ENSSSCG(	protein_coding		ENSSSCT0	2
1	92032342		C	CA	1615.99		213	1/1	1/1	1/1	0/1	0/1	0/1	0/0	intergenic_	MODIFIER	NONE	n.null_nullinsA		-1	TSPYL1-DSE				-1
1	1.04E+08		GT	G	1055.61		249	1/1	0/1	1/1	0/1	0/1	1/1	0/0	intergenic_	MODIFIER	NONE			-1	MB21D1-ENSSSCG00000025743				-1
1	1.04E+08		G	T	1112.95		116	1/1	1/1	1/1	0/1	0/1	1/1	0/0	intergenic_	MODIFIER	NONE			-1	ENSSSCG00000025743-SETBP1				-1
1	1.23E+08		A	AT	1777.79		236	1/1	1/1	1/1	0/1	0/1	0/1	0/0	intergenic_	MODIFIER	NONE	n.null_nullinsT		-1	ANXA2-ENSSSCG00000004580				-1
1	1.29E+08		ATTT	A	3815.41		228	1/1	1/1	1/1	0/1	0/1	1/1	0/0	intergenic_	MODIFIER	NONE	n.null_nulldelTTT		-1	ENSSSCG00000029459-NEDD4				-1
1	1.37E+08		A	ACG	5570.43		214	1/1	1/1	1/1	0/1	0/0	1/1	0/0	intergenic_	MODIFIER	NONE	n.null_nullinsCG		-1	ENSSSCG00000004658-DUT				-1
1	1.41E+08		AAAC	A	5705.44		202	1/1	1/1	1/1	0/1	0/1	1/1	0/0	intron_vari	MODIFIER	NONE	c.1045-155_1045-153		-1	DUOX2	protein_coding		ENSSSCT0	8
1	3.09E+08		T	C	1172.76		171	1/1	1/1	1/1	0/1	0/1	1/1	0/0	intergenic_	MODIFIER	NONE			-1	ENSSSCG00000005763-ENSSSCG00000025				-1
2	14567383		A	G	1578.91		135	0/0	0/0	0/0	1/1	1/1	./.	1/1	intergenic_	MODIFIER	NONE			-1	ENSSSCG00000024728-ENSSSCG00000014				-1
2	18863926		T	TA	1322.01		224	0/0	0/1	0/0	0/1	0/1	0/1	1/1	intergenic_	MODIFIER	NONE	n.null_nullinsA		-1	PRDM11-TP53I11				-1
2	19565912		C	CG	1377.89		149	1/1	1/1	1/1	0/1	0/1	0/1	0/0	intergenic_	MODIFIER	NONE	n.null_nullinsG		-1	U5-EXT2				-1
2	27349923		C	T	2201.19		198	1/1	1/1	1/1	0/1	0/1	0/1	0/0	intron_vari	MODIFIER	NONE	c.320-2211G>A		-1	LDLRAD3	protein_coding		ENSSSCT0	3
2	28815481		T	C	3404.02		229	1/1	1/1	1/1	0/1	0/1	1/1	0/0	intergenic_	MODIFIER	NONE			-1	EHF-ELF5				-1
2	43282851		GT	G	3288.51		185	1/1	1/1	1/1	0/1	0/1	0/1	0/0	upstream_	MODIFIER	NONE	c.-104delA		-1	CSRP3	protein_coding		ENSSSCT0	-1
2	43282851		GT	G	3288.51		185	1/1	1/1	1/1	0/1	0/1	0/1	0/0	intergenic_	MODIFIER	NONE			-1	CSRP3-ENSSSCG00000021688				-1
3	25213842		G	A	1519.45		174	0/0	0/1	0/1	1/1	1/1	0/1	1/1	intergenic_	MODIFIER	NONE			-1	ENSSSCG00000007847-ENSSSCG00000007				-1
3	27990223		G	A	1259.01		155	0/0	0/1	0/1	1/1	1/1	0/1	1/1	intergenic_	MODIFIER	NONE			-1	ENSSSCG00000007872-5S_rRNA				-1
3	28057532		A	T	2031		217	0/0	0/1	0/1	0/1	0/1	0/1	1/1	intergenic_	MODIFIER	NONE			-1	ENSSSCG00000007872-5S_rRNA				-1
3	29467453		G	A	1111.54		208	0/0	0/1	0/1	1/1	1/1	0/1	1/1	intron_vari	MODIFIER	NONE	c.289+10436G>A		-1	CPPED1	protein_coding		ENSSSCT0	2
3	32430658		C	A	1259.52		132	0/0	0/1	0/1	1/1	1/1	0/1	1/1	intergenic_	MODIFIER	NONE			-1	LITAF-ENSSSCG00000028743				-1
3	33134141		C	T	1105.93		145	0/0	1/1	0/1	1/1	1/1	0/1	1/1	intron_vari	MODIFIER	NONE	c.376+331C>T		-1	ENSSSCG(	protein_coding		ENSSSCT0	5
3	1.39E+08		C	T	1163.73		148	0/0	0/1	0/1	1/1	1/1	0/1	1/1	intergenic_	MODIFIER	NONE			-1	ENSSSCG00000025247-ASGR1				-1
3	12643285		A	T	1239.73		141	1/1	1/1	1/1	1/1	1/1	1/1	0/0	intergenic_	MODIFIER	NONE			-1	ENSSSCG00000024045-ZNF226				-1
3	1.09E+08		G	A	1334.41		150	1/1	1/1	1/1	0/1	0/1	1/1	0/0	intron_vari	MODIFIER	NONE	c.540+264C>T		-1	ENSSSCG(	protein_coding		ENSSSCT0	5
3	1.11E+08		T	C	1103.15		151	1/1	1/1	1/1	0/1	0/1	1/1	0/0	intergenic_	MODIFIER	NONE			-1	CRIM1-ENSSSCG00000008506				-1
3	1.11E+08		A	G	1204.03		151	1/1	1/1	1/1	0/1	0/1	1/1	0/0	intergenic_	MODIFIER	NONE			-1	CRIM1-ENSSSCG00000008506				-1
3	1.19E+08		TA	T	1318.87		160	1/1	1/1	1/1	0/1	0/1	1/1	0/0	intron_vari	MODIFIER	NONE	c.139-1475delT		-1	ABHD1	protein_coding		ENSSSCT0	1
3	1.19E+08		CT	C	5165.86		230	1/1	1/1	1/1	0/1	0/1	1/1	0/0	intron_vari	MODIFIER	NONE	c.624+60delA		-1	MAPRE3	protein_coding		ENSSSCT0	5
3	1.42E+08		A	T	3230.05		2018	1/1	0/1	1/1	1/1	0/1	1/1	0/0	intergenic_	MODIFIER	NONE			-1	ACP1-ENSSSCG00000008661				-1
3	1.43E+08		T	G	1914.87		568	1/1	0/1	0/1	1/1	1/1	1/1	0/0	intergenic_	MODIFIER	NONE			-1	ACP1-ENSSSCG00000008661				-1
4	4068921		T	TG	1661.78		218	0/0	0/1	0/1	0/1	1/1	1/1	1/1	intergenic_	MODIFIER	NONE	n.null_nullinsG		-1	ENSSSCG00000005938-5S_rRNA				-1
4	9068490		T	C	1288.22		135	0/0	0/1	0/1	1/1	1/1	0/1	1/1	intron_vari	MODIFIER	NONE	c.169+46T>C		-1	OC90	protein_coding		ENSSSCT0	2
4	9068533		C	G	1109.13		130	0/0	0/1	0/1	1/1	1/1	0/1	1/1	intron_vari	MODIFIER	NONE	c.169+89C>G		-1	OC90	protein_coding		ENSSSCT0	2
4	9084210		T	C	1281.3		160	0/0	0/1	0/1	1/1	1/1	0/1	1/1	intron_vari	MODIFIER	NONE	c.1070+848T>C		-1	OC90	protein_coding		ENSSSCT0	12
4	9086006		T	C	1388.05		212	0/0	0/1	0/1	1/1	1/1	0/1	1/1	intron_vari	MODIFIER	NONE	c.1071-180T>C		-1	OC90	protein_coding		ENSSSCT0	12
4	20598904		C	CT	1482.44		258	0/0	0/1	0/0	0/1	0/1	1/1	1/1	intergenic_	MODIFIER	NONE	n.null_nullinsT		-1	NOV-MAL2				-1
4	43291944		CAAAAAA	C	3569.67		156	0/0	0/1	0/1	0/1	0/1	1/1	1/1	intergenic_	MODIFIER	NONE	n.null_nulldelAAAAA		-1	SDC2-PTDSS1				-1
4	67994103		TTG	T	1837.94		214	0/0	0/0	0/1	1/1	1/1	0/1	1/1	intron_vari	MODIFIER	NONE	c.814+7317_814+731		-1	STAU2	protein_coding		ENSSSCT0	6
4	74051994		T	C	1357.71		165	0/0	0/1	0/1	1/1	1/1	1/1	1/1	intergenic_	MODIFIER	NONE			-1	ENSSSCG00000006206-ENSSSCG00000023				-1
4	74052001		T	C	1158.98		164	0/0	0/1	0/1	1/1	1/1	1/1	1/1	intergenic_	MODIFIER	NONE			-1	ENSSSCG00000006206-ENSSSCG00000023				-1
4	84082675		GA	G	3538.89		265	0/0	0/0	0/1	1/1	1/1	1/1	1/1	intergenic_	MODIFIER	NONE			-1	SOX17-MRPL15				-1
4	86371133		A	G	1238.81		164	0/0	0/1	0/1	1/1	1/1	1/1	1/1	intergenic_	MODIFIER	NONE			-1	PCMTD1-SNAI2				-1

Head	Note
#CHROM	Chromosome
POS	Position
ID	Identification
REF	Reference seq (Sscrofa 10.2)
ALT	Alternative sequence
QUAL	Quality
FILTER	
DP	Total depth
EFF[*].EFFE	Genetic element
EFF[*].IMP	Functional annotation
EFF[*].FUNCLASS	
EFF[*].CODON	
EFF[*].AA	
EFF[*].AA_LEN	
EFF[*].GENE	
EFF[*].BIOTYPE	
EFF[*].CODING	
EFF[*].TRID	
EFF[*].RANK	
./.	Not called
0/0	Homogeneous to REF
0/1	Heterogeneous to REF
1/1	Homogeneous to ALT

4	92282644	T	C	1536.93	143	0/0	0/1	0/1	0/1	0/1	0/1	1/1	intron_var	MODIFIER	NONE	c.263+504T>C		-1	FAM78B	protein_coding	ENSSSCT0	1
4	1.03E+08	T	C	1067.01	145	0/0	0/1	0/1	0/1	0/1	0/1	1/1	missense_v	MODERAT	NONE	c.1385A>(	p.His462A	1359	ENSSSCG(	protein_coding	ENSSSCT0	2
4	1.04E+08	C	G	1037.16	144	0/0	0/1	0/1	0/1	0/1	0/1	1/1	upstream_	MODIFIER	NONE	c.-137G>C		-1	ENSSSCG(	protein_coding	ENSSSCT0	-1
4	1.04E+08	C	G	1037.16	144	0/0	0/1	0/1	0/1	0/1	0/1	1/1	intergenic_	MODIFIER	NONE			-1	ENSSSCG00000006533-ZBTB7B			-1
4	1.3E+08	GA	G	1112.08	164	0/0	0/1	0/1	0/1	0/1	1/1	1/1	intergenic_	MODIFIER	NONE			-1	ENSSSCG00000006870-SLC35A3			-1
4	9016480	T	A	1259.96	195	1/1	1/1	1/1	1/1	1/1	./.	0/0	intergenic_	MODIFIER	NONE			-1	ENSSSCG00000024124-ENSSSCG00000022			-1
4	9018024	G	A	1430.91	144	1/1	1/1	1/1	1/1	1/1	0/1	0/0	intergenic_	MODIFIER	NONE			-1	ENSSSCG00000024124-ENSSSCG00000022			-1
4	35485513	CT	C	1981.55	214	1/1	1/1	0/1	1/1	1/1	1/1	0/0	intron_var	MODIFIER	NONE	c.137-7728delT		-1	LRP12	protein_coding	ENSSSCT0	2
4	45216561	A	G	1527.31	122	1/1	1/1	1/1	1/1	1/1	./.	0/0	intergenic_	MODIFIER	NONE			-1	INTS8-DPY19L4			-1
4	1.22E+08	T	G	4592.32	155	1/1	1/1	1/1	1/1	1/1	0/1	0/0	intergenic_	MODIFIER	NONE			-1	FAM102B-SCAMC-1			-1
4	1.24E+08	T	C	1413.31	128	1/1	1/1	1/1	1/1	1/1	0/1	0/0	intergenic_	MODIFIER	NONE			-1	ENSSSCG00000006853-U6			-1
4	1.25E+08	TC	T	2908.45	212	1/1	1/1	1/1	1/1	1/1	0/1	0/0	intergenic_	MODIFIER	NONE			-1	U6-ENSSSCG00000025216			-1
4	1.25E+08	AAAC	A	3378.8	189	1/1	1/1	1/1	1/1	1/1	0/1	0/0	intergenic_	MODIFIER	NONE	n.null_nulldelAAC		-1	U6-ENSSSCG00000025216			-1
5	7578522	G	C	1089.97	137	0/0	0/1	0/1	0/1	0/1	0/1	1/1	intron_var	MODIFIER	NONE	c.4813-167C>G		-1	ENSSSCG(	protein_coding	ENSSSCT0	15
5	9089370	C	CT	2230.04	216	0/0	0/0	0/0	1/1	1/1	1/1	1/1	intron_var	MODIFIER	NONE	c.3101-81_3101-80ins		-1	MYH9	protein_coding	ENSSSCT0	24
5	27974160	A	G	1202.27	146	0/0	0/1	0/1	1/1	1/1	0/1	1/1	intergenic_	MODIFIER	NONE			-1	U6-ENSSSCG00000000457			-1
5	69132974	GA	G	2087.8	234	0/0	0/0	0/1	1/1	1/1	0/1	1/1	intergenic_	MODIFIER	NONE			-1	PRMT8-ENSSSCG00000000734			-1
5	33392417	T	C	1910.95	138	1/1	1/1	1/1	0/1	0/1	1/1	0/0	intron_var	MODIFIER	NONE	c.249+2661T>C		-1	HMGA2	protein_coding	ENSSSCT0	3
5	33392433	G	T	1170.46	121	1/1	1/1	1/1	0/1	0/1	1/1	0/0	intron_var	MODIFIER	NONE	c.249+2677G>T		-1	HMGA2	protein_coding	ENSSSCT0	3
5	33394214	A	G	1291.83	141	1/1	1/1	1/1	0/1	0/1	1/1	0/0	intron_var	MODIFIER	NONE	c.249+4458A>G		-1	HMGA2	protein_coding	ENSSSCT0	3
5	40150123	A	C	1168.24	140	1/1	1/1	1/1	0/1	0/1	1/1	0/0	intergenic_	MODIFIER	NONE			-1	U4-ENSSSCG00000023758			-1
5	47253705	A	G	1737.49	160	1/1	1/1	1/1	0/1	0/1	1/1	0/0	intron_var	MODIFIER	NONE	c.555-11068A>G		-1	ENSSSCG(	protein_coding	ENSSSCT0	3
5	67490871	G	GCA	3401.17	233	1/1	1/1	1/1	0/0	0/0	1/1	0/0	intergenic_	MODIFIER	NONE	n.null_nullinsCA		-1	ANO2-KV1.5			-1
5	69456012	GA	G	1469.67	218	1/1	1/1	1/1	0/1	1/1	0/1	0/0	intron_var	MODIFIER	NONE	c.-29-657delT		-1	TEAD4	protein_coding	ENSSSCT0	2
6	17071811	A	C	1049.52	1905	0/0	0/1	0/1	1/1	1/1	1/1	1/1	intergenic_	MODIFIER	NONE			-1	CDH3-RSPRY1			-1
6	29037648	A	G	1211.43	115	0/0	0/1	0/1	1/1	1/1	1/1	1/1	intergenic_	MODIFIER	NONE			-1	ENSSSCG00000002834-TOX3			-1
6	31093904	A	G	1081.53	127	0/0	0/1	0/1	1/1	1/1	1/1	1/1	intergenic_	MODIFIER	NONE			-1	ZNF423-N4BP1			-1
6	59974516	T	C	1514.54	134	0/0	0/1	0/1	1/1	1/1	1/1	1/1	intergenic_	MODIFIER	NONE			-1	ENSSSCG00000003361-C1orf174			-1
6	75048956	T	C	1056.88	139	0/0	0/1	0/1	1/1	1/1	0/1	1/1	intron_var	MODIFIER	NONE	c.1405-11327T>C		-1	KDM1A	protein_coding	ENSSSCT0	11
6	78665344	GA	G	1779.58	202	0/0	0/0	0/1	0/1	0/1	1/1	1/1	intron_var	MODIFIER	NONE	c.572-1071delT		-1	EYA3	protein_coding	ENSSSCT0	4
6	81281307	G	GT	2735.98	183	0/0	0/1	1/1	1/1	0/1	0/1	1/1	intron_var	MODIFIER	NONE	c.820-502_820-501ins		-1	SDC3	protein_coding	ENSSSCT0	2
6	84569119	CG	C	3720.18	245	0/0	0/0	0/1	0/1	0/1	1/1	1/1	intergenic_	MODIFIER	NONE			-1	C1orf94-ENSSSCG00000003624			-1
6	10060732	A	G	1476.56	141	1/1	1/1	1/1	1/1	1/1	0/1	0/0	intron_var	MODIFIER	NONE	c.230+1050T>C		-1	VAT1L	protein_coding	ENSSSCT0	1
6	23030258	C	A	3401.38	239	1/1	0/1	0/1	0/1	0/1	0/1	0/0	intergenic_	MODIFIER	NONE			-1	U2-CDH11			-1
6	32768657	G	A	5142.53	311	1/1	1/1	1/1	1/1	1/1	0/1	0/0	intergenic_	MODIFIER	NONE			-1	ENSSSCG00000026318-GPT2			-1
6	60068443	G	A	1369.15	162	1/1	1/1	1/1	1/1	1/1	1/1	0/0	intergenic_	MODIFIER	NONE			-1	C1orf174-ssc-mir-4331			-1
6	60500943	G	A	1575.64	192	1/1	1/1	1/1	0/1	0/1	1/1	0/0	intergenic_	MODIFIER	NONE			-1	DFFB-NPHP4			-1
6	60582914	G	A	1383.97	174	1/1	1/1	1/1	0/1	0/1	1/1	0/0	intergenic_	MODIFIER	NONE			-1	DFFB-NPHP4			-1
6	60600187	G	T	1228.13	151	1/1	1/1	1/1	0/1	0/1	1/1	0/0	intergenic_	MODIFIER	NONE			-1	DFFB-NPHP4			-1
6	75789726	G	T	5039.15	312	1/1	1/1	1/1	0/1	0/1	0/1	0/0	intron_var	MODIFIER	NONE	c.73+1526C>A		-1	STPG1	protein_coding	ENSSSCT0	2
6	91341447	G	A	1149.68	149	1/1	1/1	1/1	0/0	0/1	1/1	0/0	intergenic_	MODIFIER	NONE			-1	ENSSSCG00000024963-ENSSSCG00000003			-1
7	23717326	T	A	1022.24	121	1/1	1/1	1/1	1/1	1/1	1/1	0/0	intergenic_	MODIFIER	NONE			-1	ENSSSCG00000001196-ENSSSCG00000022			-1
7	27945935	G	T	1939.72	172	1/1	1/1	1/1	0/1	1/1	1/1	0/0	intron_var	MODIFIER	NONE	c.2234-385C>A		-1	ENSSSCG(	protein_coding	ENSSSCT0	7
7	30942918	A	G	1668.46	143	1/1	1/1	1/1	1/1	1/1	0/1	0/0	intergenic_	MODIFIER	NONE			-1	FAM83B-ENSSSCG00000001484			-1
7	32887747	C	T	1395.69	145	1/1	1/1	1/1	1/1	1/1	0/1	0/0	intron_var	MODIFIER	NONE	c.694-9287G>A		-1	ENSSSCG(	protein_coding	ENSSSCT0	7
7	33397853	C	G	1330.04	137	1/1	1/1	1/1	1/1	1/1	0/1	0/0	intergenic_	MODIFIER	NONE			-1	ENSSSCG00000025172-DST			-1
7	33457871	A	G	1236.56	128	1/1	1/1	1/1	1/1	1/1	1/1	0/0	intergenic_	MODIFIER	NONE			-1	ENSSSCG00000025172-DST			-1
7	33460465	C	T	1195.57	142	1/1	1/1	1/1	1/1	1/1	0/1	0/0	intergenic_	MODIFIER	NONE			-1	ENSSSCG00000025172-DST			-1
7	35907715	G	C	1119.49	124	1/1	1/1	1/1	1/1	1/1	1/1	0/0	intron_var	MODIFIER	NONE	c.272-33079C>G		-1	SCUBE3	protein_coding	ENSSSCT0	2
7	38825386	G	A	1070.45	110	1/1	1/1	1/1	1/1	1/1	0/1	0/0	intron_var	MODIFIER	NONE	c.29-33123G>A		-1	ENSSSCG(	protein_coding	ENSSSCT0	1
7	51077725	C	T	1593.19	146	1/1	1/1	1/1	1/1	1/1	1/1	0/0	intergenic_	MODIFIER	NONE			-1	TFAP2D-ENSSSCG00000025535			-1
7	52012140	C	G	1371.3	160	1/1	1/1	1/1	0/1	0/1	0/1	0/0	intergenic_	MODIFIER	NONE			-1	ENSSSCG00000028857-ENSSSCG00000001			-1
7	52012211	G	A	1202.75	152	1/1	1/1	0/1	0/1	0/1	0/1	0/0	intergenic_	MODIFIER	NONE			-1	ENSSSCG00000028857-ENSSSCG00000001			-1
7	52012263	C	G	1104.48	151	1/1	1/1	0/1	0/1	0/1	0/1	0/0	intergenic_	MODIFIER	NONE			-1	ENSSSCG00000028857-ENSSSCG00000001			-1
7	1.21E+08	A	T	1508.1	131	1/1	1/1	1/1	1/1	1/1	0/1	0/0	intron_var	MODIFIER	NONE	c.2438-5174A>T		-1	ENSSSCG(	protein_coding	ENSSSCT0	8
8	5768168	AG	A	1536.66	196	0/0	0/1	0/1	1/1	1/1	0/1	1/1	intergenic_	MODIFIER	NONE			-1	TMEM128-LYAR			-1
8	21945383	A	G	1116.71	139	0/0	0/1	1/1	1/1	1/1	0/1	1/1	intergenic_	MODIFIER	NONE			-1	7SK-ENSSSCG00000008763			-1
8	21945384	T	C	1257.37	140	0/0	0/1	1/1	1/1	1/1	1/1	1/1	intergenic_	MODIFIER	NONE			-1	7SK-ENSSSCG00000008763			-1
8	23009229	CG	C	1080.68	241	0/0	0/0	0/0	1/1	1/1	1/1	1/1	intergenic_	MODIFIER	NONE			-1	ENSSSCG00000008763-PCDH7			-1
8	26874123	A	C	1342.97	136	0/0	0/1	0/1	1/1	1/1	1/1	1/1	intergenic_	MODIFIER	NONE			-1	PCDH7-ENSSSCG00000008767			-1
8	39555553	T	TTA	4723.47	227	0/0	0/0	0/0	1/1	1/1	1/1	1/1	intron_var	MODIFIER	NONE	c.2411+1756_2411+1		-1	CORIN	protein_coding	ENSSSCT0	16
8	45200463	CT	C	2816.01	228	0/0	0/1	0/0	1/1	1/1	1/1	1/1	intergenic_	MODIFIER	NONE			-1	ENSSSCG00000024269-CPE			-1
8	2668800	G	A	2410.49	154	1/1	1/1	1/1	0/1	0/1	1/1	0/0	intergenic_	MODIFIER	NONE			-1	C4orf50-JAKMIP1			-1
8	9493642	AAAC	A	1538.8	300	1/1	0/1	1/1	0/1	0/1	0/1	0/0	intergenic_	MODIFIER	NONE	n.null_nulldelAAC		-1	BOD1L1-CPEB2			-1
8	9589033	T	C	1016.22	168	1/1	1/1	1/1	0/1	0/1	1/1	0/0	intergenic_	MODIFIER	NONE			-1	BOD1L1-CPEB2			-1
8	9589091	T	C	1618.95	158	1/1	1/1	1/1	0/1	0/1	1/1	0/0	intergenic_	MODIFIER	NONE			-1	BOD1L1-CPEB2			-1
8	32060822	AC	A	3262.27	219	1/1	0/1	1/1	0/1	0/1	0/1	0/0	intergenic_	MODIFIER	NONE			-1	ENSSSCG00000030201-WDR19			-1

8	75790028	G	A	3313.66	386	1/1	1/1	1/1	0/0	0/0	0/1	0/0	intergenic_	MODIFIER	NONE		-1	SDAD1-CXCL9			-1
8	91358977	T	A	1570.86	200	1/1	1/1	1/1	1/1	1/1	0/1	0/0	intergenic_	MODIFIER	NONE		-1	ENSSSCG00000009052-RNF150			-1
8	91368760	C	A	1054.41	107	1/1	1/1	1/1	1/1	1/1	0/1	0/0	intergenic_	MODIFIER	NONE		-1	ENSSSCG00000009052-RNF150			-1
8	91368764	T	C	1009.58	106	1/1	1/1	1/1	1/1	1/1	0/1	0/0	intergenic_	MODIFIER	NONE		-1	ENSSSCG00000009052-RNF150			-1
8	91409824	T	C	1056.84	151	1/1	1/1	1/1	1/1	1/1	0/1	0/0	intergenic_	MODIFIER	NONE		-1	ENSSSCG00000009052-RNF150			-1
8	91480263	A	G	1033.28	130	1/1	1/1	1/1	1/1	1/1	0/1	0/0	intergenic_	MODIFIER	NONE		-1	ENSSSCG00000009052-RNF150			-1
8	91484661	T	C	1304.76	134	1/1	1/1	1/1	1/1	1/1	0/1	0/0	intergenic_	MODIFIER	NONE		-1	ENSSSCG00000009052-RNF150			-1
8	1.15E+08	A	G	1318.94	121	1/1	1/1	1/1	1/1	1/1	0/1	0/0	intergenic_	MODIFIER	NONE		-1	ENSSSCG00000009117-ENSSSCG00000009			-1
9	4683739	A	G	1065.02	152	0/0	1/1	0/1	1/1	1/1	1/1	1/1	intergenic_	MODIFIER	NONE		-1	ENSSSCG00000014673-TRIM6			-1
9	55737157	G	T	1022.89	124	0/0	0/1	0/1	1/1	1/1	1/1	1/1	intergenic_	MODIFIER	NONE		-1	ENSSSCG00000015140-ENSSSCG00000015			-1
9	56254834	T	A	2035.29	138	0/0	0/1	0/1	1/1	1/1	1/1	1/1	intergenic_	MODIFIER	NONE		-1	ENSSSCG00000015162-ENSSSCG00000025			-1
9	90821723	G	GA	1029.34	224	0/0	0/1	0/1	1/1	0/1	0/1	1/1	intergenic_	MODIFIER	NONE	n.null_nullinsA	-1	ARL4A-ETV1			-1
9	91512293	GT	G	1005.36	271	0/0	0/0	0/1	0/1	0/1	0/1	1/1	intron_vari	MODIFIER	NONE	c.1114-1420delA	-1	ETV1	protein_coding	ENSSSCT0	11
9	1E+08	T	TGG	1325.41	223	0/0	0/0	0/1	1/1	1/1	0/1	1/1	intron_vari	MODIFIER	NONE	c.11403+301_11403+	-1	DNAH11	protein_coding	ENSSSCT0	71
9	1E+08	G	A	1425.94	173	0/0	0/1	0/1	1/1	1/1	0/1	1/1	intron_vari	MODIFIER	NONE	c.12103-726G>A	-1	DNAH11	protein_coding	ENSSSCT0	75
9	1E+08	A	C	1166.23	168	0/0	0/1	0/1	1/1	1/1	0/1	1/1	intron_vari	MODIFIER	NONE	c.12103-708A>C	-1	DNAH11	protein_coding	ENSSSCT0	75
9	1.01E+08	A	G	1067.48	130	0/0	0/1	0/1	1/1	1/1	0/1	1/1	upstream_	MODIFIER	NONE	c.-64A>G	-1	IL6	protein_coding	ENSSSCT0	-1
9	1.01E+08	A	G	1067.48	130	0/0	0/1	0/1	1/1	1/1	0/1	1/1	intergenic_	MODIFIER	NONE		-1	TOMM7-IL6			-1
9	16757028	A	AC	1021.99	232	1/1	1/1	1/1	1/1	1/1	0/1	0/0	intergenic_	MODIFIER	NONE	n.null_nullinsC	-1	ENSSSCG00000014896-U6			-1
9	29131911	T	C	1176.04	149	1/1	1/1	1/1	1/1	1/1	0/0	0/0	intron_vari	MODIFIER	NONE	c.-26-1780T>C	-1	ENSSSCG(	protein_coding	ENSSSCT0	2
9	45966872	A	G	1522.75	115	1/1	1/1	1/1	1/1	1/1	1/1	0/0	intergenic_	MODIFIER	NONE		-1	DRD2-U6			-1
9	55801778	A	G	1633.51	150	1/1	1/1	1/1	1/1	1/1	1/1	0/0	intron_vari	MODIFIER	NONE	c.585-827T>C	-1	ENSSSCG(	protein_coding	ENSSSCT0	4
9	55853043	CT	C	2191.44	202	1/1	1/1	1/1	0/1	1/1	1/1	0/0	intergenic_	MODIFIER	NONE		-1	ENSSSCG00000024299-ZNF202			-1
9	62639271	TA	T	1317.69	202	1/1	1/1	1/1	0/1	0/1	0/0	0/0	intergenic_	MODIFIER	NONE		-1	BARX2-SNORD112			-1
9	73656311	CT	C	2141.82	193	1/1	1/1	1/1	1/1	1/1	1/1	0/0	upstream_	MODIFIER	NONE	c.-1delA	-1	DYRK3	protein_coding	ENSSSCT0	-1
9	73656311	CT	C	2141.82	193	1/1	1/1	1/1	1/1	1/1	1/1	0/0	intergenic_	MODIFIER	NONE		-1	DYRK3-ENSSSCG00000015650			-1
9	75774162	A	G	5306.54	307	1/1	1/1	1/1	1/1	1/1	0/1	0/0	intergenic_	MODIFIER	NONE		-1	U6-ZNF804B			-1
9	86160521	C	T	6404.54	260	1/1	1/1	1/1	1/1	1/1	0/1	0/0	intergenic_	MODIFIER	NONE		-1	U1-ENSSSCG00000015346			-1
9	1.22E+08	G	GT	2286.16	274	1/1	1/1	1/1	1/1	1/1	0/1	0/0	intergenic_	MODIFIER	NONE	n.null_nullinsT	-1	ENSSSCG00000015460-ENSSSCG00000021			-1
9	1.34E+08	T	C	2700.16	457	1/1	1/1	1/1	1/1	1/1	1/1	0/0	intergenic_	MODIFIER	NONE		-1	XPR1-KIAA1614			-1
10	29938018	A	C	3839.15	362	0/0	0/1	0/1	1/1	1/1	0/1	1/1	intergenic_	MODIFIER	NONE		-1	ENSSSCG00000010935-ENSSSCG00000010			-1
10	64031898	A	G	1811.04	138	0/0	0/1	0/1	1/1	1/1	1/1	1/1	intergenic_	MODIFIER	NONE		-1	GJD4-CCDC3			-1
10	13394424	CG	C	1884.53	186	1/1	1/1	1/1	1/1	1/1	1/1	0/0	intergenic_	MODIFIER	NONE		-1	DUSP10-HHIPL2			-1
10	32908624	C	CT	1235.55	206	1/1	0/1	1/1	0/0	0/1	1/1	0/0	intergenic_	MODIFIER	NONE	n.null_nullinsT	-1	DAPK1-ZCCHC6			-1
10	38489003	T	G	1998.66	180	1/1	1/1	1/1	0/1	0/1	1/1	0/0	intergenic_	MODIFIER	NONE		-1	SNORA31-SNORA31			-1
10	38853301	TAA	T	3562.76	182	1/1	1/1	1/1	0/0	0/0	1/1	0/0	intergenic_	MODIFIER	NONE	n.null_nulldelAA	-1	SNORA31-U6			-1
10	44424052	G	A	2388.15	279	1/1	1/1	1/1	0/1	0/0	1/1	0/0	intergenic_	MODIFIER	NONE		-1	BAMBI-ENSSSCG00000011017			-1
10	44424058	T	C	2818.19	289	1/1	1/1	1/1	0/1	0/0	1/1	0/0	intergenic_	MODIFIER	NONE		-1	BAMBI-ENSSSCG00000011017			-1
10	48437750	G	A	1529.84	126	1/1	1/1	1/1	0/1	0/1	0/1	0/0	intergenic_	MODIFIER	NONE		-1	ST8SIA6-ENSSSCG00000011035			-1
10	48593344	C	A	1933.02	171	1/1	1/1	1/1	0/1	0/1	1/1	0/0	intergenic_	MODIFIER	NONE		-1	ST8SIA6-ENSSSCG00000011035			-1
10	48617088	G	A	1028.86	117	1/1	1/1	1/1	0/1	0/1	1/1	0/0	intron_vari	MODIFIER	NONE	c.11+2490C>T	-1	ENSSSCG(	protein_coding	ENSSSCT0	1
10	51413315	G	GA	1716.06	184	1/1	1/1	1/1	0/1	0/0	1/1	0/0	upstream_	MODIFIER	NONE	c.-150_-150insA	-1	NMT2	protein_coding	ENSSSCT0	-1
10	51413315	G	GA	1716.06	184	1/1	1/1	1/1	0/1	0/0	1/1	0/0	intergenic_	MODIFIER	NONE	n.null_nullinsA	-1	U6-NMT2			-1
10	57034243	A	AAACCC	7505.61	207	1/1	1/1	1/1	0/1	0/1	1/1	0/0	intergenic_	MODIFIER	NONE	n.null_nullinsAACCC	-1	ENSSSCG00000022730-ENSSSCG00000011			-1
10	62544164	G	A	1554.13	163	1/1	1/1	1/1	0/1	0/1	1/1	0/0	intergenic_	MODIFIER	NONE		-1	NRP1-ENSSSCG00000011103			-1
10	62544168	T	G	1802	162	1/1	1/1	1/1	0/1	0/1	1/1	0/0	intergenic_	MODIFIER	NONE		-1	NRP1-ENSSSCG00000011103			-1
10	63095377	G	GA	2400.82	215	1/1	1/1	1/1	0/1	0/1	1/1	0/0	intergenic_	MODIFIER	NONE	n.null_nullinsA	-1	ENSSSCG00000011103-CUL2			-1
11	71093622	A	T	1221.43	211	0/0	0/1	0/1	1/1	1/1	0/1	1/1	intron_vari	MODIFIER	NONE	c.273+592A>T	-1	ENSSSCG(	protein_coding	ENSSSCT0	2
11	80970713	G	A	1544.19	140	0/0	0/1	0/1	1/1	1/1	0/1	1/1	intergenic_	MODIFIER	NONE		-1	ENSSSCG00000026317-EFNB2			-1
11	37797467	G	T	1017.02	135	1/1	1/1	1/1	0/1	0/1	1/1	0/0	intergenic_	MODIFIER	NONE		-1	SNORA31-ENSSSCG00000024171			-1
11	51921183	C	A	1009.08	144	1/1	1/1	1/1	1/1	1/1	1/1	0/0	intergenic_	MODIFIER	NONE		-1	KLF5-ENSSSCG00000009465			-1
11	51921220	A	G	1346.54	142	1/1	1/1	1/1	1/1	1/1	1/1	0/0	intergenic_	MODIFIER	NONE		-1	KLF5-ENSSSCG00000009465			-1
11	51932409	G	A	1045.83	140	1/1	1/1	1/1	1/1	1/1	1/1	0/0	intergenic_	MODIFIER	NONE		-1	KLF5-ENSSSCG00000009465			-1
11	52118232	A	G	1134.77	136	1/1	1/1	1/1	1/1	1/1	1/1	0/0	intergenic_	MODIFIER	NONE		-1	KLF5-ENSSSCG00000009465			-1
11	52134003	C	T	1699.49	143	1/1	1/1	1/1	1/1	1/1	1/1	0/0	intergenic_	MODIFIER	NONE		-1	KLF5-ENSSSCG00000009465			-1
11	52818703	A	C	2078.5	253	1/1	1/1	1/1	1/1	1/1	1/1	0/0	intergenic_	MODIFIER	NONE		-1	LMO7-KCTD12			-1
11	53248011	T	C	1442.41	133	1/1	1/1	1/1	1/1	1/1	1/1	0/0	intergenic_	MODIFIER	NONE		-1	LMO7-KCTD12			-1
11	53248023	A	G	1486.66	141	1/1	1/1	1/1	1/1	1/1	1/1	0/0	intergenic_	MODIFIER	NONE		-1	LMO7-KCTD12			-1
11	53261058	T	C	2019.53	167	1/1	1/1	1/1	1/1	1/1	1/1	0/0	intergenic_	MODIFIER	NONE		-1	LMO7-KCTD12			-1
11	53286966	A	C	1110.59	162	1/1	1/1	1/1	1/1	1/1	1/1	0/0	intergenic_	MODIFIER	NONE		-1	LMO7-KCTD12			-1
11	55435086	C	G	1805.99	151	1/1	1/1	1/1	1/1	1/1	1/1	0/0	intron_vari	MODIFIER	NONE	c.639-1315G>C	-1	RNF219	protein_coding	ENSSSCT0	5
11	62041272	G	A	1574.61	148	1/1	1/1	1/1	0/1	1/1	1/1	0/0	intergenic_	MODIFIER	NONE		-1	SLITRK6-ENSSSCG00000024241			-1
11	62971203	A	C	1353.84	144	1/1	1/1	1/1	1/1	1/1	1/1	0/0	intergenic_	MODIFIER	NONE		-1	ENSSSCG00000024241-ENSSSCG00000029			-1
11	69609751	T	C	1278.55	163	1/1	1/1	1/1	1/1	1/1	1/1	0/0	upstream_	MODIFIER	NONE	c.-1T>C	-1	GPC6	protein_coding	ENSSSCT0	-1
11	69609751	T	C	1278.55	163	1/1	1/1	1/1	1/1	1/1	1/1	0/0	intergenic_	MODIFIER	NONE		-1	U6-GPC6			-1
11	75430106	C	G	1822.31	125	1/1	1/1	1/1	1/1	1/1	1/1	0/0	upstream_	MODIFIER	NONE	c.-253G>C	-1	ENSSSCG(	protein_coding	ENSSSCT0	-1
11	75430106	C	G	1822.31	125	1/1	1/1	1/1	1/1	1/1	1/1	0/0	intron_vari	MODIFIER	NONE	c.389+14866C>G	-1	UBAC2	protein_coding	ENSSSCT0	4

11	77206321	T	C	1797.4	124	1/1	1/1	1/1	1/1	1/1	1/1	0/0	intron_var	MODIFIER	NONE	c.490-7801T>C	-1	ENSSSCG(	protein_coding	ENSSSCT0	3
11	79497765	A	AT	1627.41	232	1/1	1/1	1/1	1/1	1/1	1/1	0/0	intergenic_	MODIFIER	NONE	n.null_nullinsT	-1	ENSSSCG00000026317-EFNB2			-1
12	4457448	C	T	5009.15	276	1/1	1/1	1/1	0/1	0/1	0/1	0/0	intergenic_	MODIFIER	NONE		-1	MGAT5B-ENSSSCG00000026070			-1
12	22828565	T	C	1095.34	137	1/1	1/1	1/1	1/1	1/1	0/0	0/0	intron_var	MODIFIER	NONE	c.707-168A>G	-1	ENSSSCG(	protein_coding	ENSSSCT0	5
12	22828628	C	A	1690.67	151	1/1	1/1	1/1	1/1	1/1	0/1	0/0	intron_var	MODIFIER	NONE	c.707-231G>T	-1	ENSSSCG(	protein_coding	ENSSSCT0	5
12	39014754	A	G	1175.76	111	1/1	1/1	1/1	0/1	0/1	1/1	0/0	intron_var	MODIFIER	NONE	c.202+7654T>C	-1	BCAS3	protein_coding	ENSSSCT0	4
12	44075390	C	CT	2390.21	193	1/1	1/1	1/1	0/1	0/1	1/1	0/0	intron_var	MODIFIER	NONE	c.2122-323_2122-322	-1	MYO1D	protein_coding	ENSSSCT0	17
12	46470807	C	A	1409.47	150	1/1	1/1	1/1	1/1	1/1	1/1	0/0	intergenic_	MODIFIER	NONE		-1	NLK-ENSSSCG00000017756			-1
12	48770304	A	G	1471.42	134	1/1	1/1	1/1	0/1	1/1	0/1	0/0	intergenic_	MODIFIER	NONE		-1	BHLHA9-U6			-1
13	403773	C	CA	3243.74	202	0/0	0/0	0/1	1/1	1/1	1/1	1/1	intron_var	MODIFIER	NONE	c.180+44121_180+44	-1	ENSSSCG(	protein_coding	ENSSSCT0	1
13	1.87E+08	G	GA	1764.93	238	0/0	0/1	0/1	1/1	1/1	1/1	1/1	intergenic_	MODIFIER	NONE	n.null_nullinsA	-1	ROBO1-ENSSSCG00000012002			-1
13	8524911	G	GTT	2883.13	220	1/1	1/1	1/1	0/1	0/1	1/1	0/0	intergenic_	MODIFIER	NONE	n.null_nullinsTT	-1	ENSSSCG00000011206-ZNF385D			-1
13	28093169	G	C	1348.71	131	1/1	1/1	1/1	1/1	1/1	1/1	0/0	intron_var	MODIFIER	NONE	c.291-17330C>G	-1	ENSSSCG(	protein_coding	ENSSSCT0	3
13	68322864	G	T	1007.55	150	1/1	1/1	1/1	1/1	1/1	1/1	0/0	intergenic_	MODIFIER	NONE		-1	ENSSSCG00000020679-GRM7			-1
13	1.37E+08	A	G	1961.92	140	1/1	1/1	1/1	1/1	1/1	1/1	0/0	intergenic_	MODIFIER	NONE		-1	LEPREL1-CLAUDIN1			-1
13	1.39E+08	CT	C	4593.04	231	1/1	1/1	1/1	1/1	1/1	1/1	0/0	intergenic_	MODIFIER	NONE		-1	ENSSSCG00000018793-FGF12			-1
13	1.63E+08	CA	C	1818.43	222	1/1	1/1	1/1	1/1	0/1	1/1	0/0	intergenic_	MODIFIER	NONE		-1	ALCAM-U1			-1
13	1.71E+08	G	A	1090.69	234	1/1	1/1	1/1	1/1	1/1	1/1	0/0	intergenic_	MODIFIER	NONE		-1	ENSSSCG00000026824-ENSSSCG00000030			-1
13	1.88E+08	C	T	1690.39	153	1/1	1/1	1/1	0/1	0/1	1/1	0/0	intergenic_	MODIFIER	NONE		-1	ENSSSCG00000012002-LIPI			-1
13	1.99E+08	T	A	1669.21	139	1/1	1/1	0/1	0/1	0/1	0/1	0/0	upstream_	MODIFIER	NONE	c.-38A>T	-1	ENSSSCG(	protein_coding	ENSSSCT0	-1
13	1.99E+08	T	A	1669.21	139	1/1	1/1	0/1	0/1	0/1	0/1	0/0	intergenic_	MODIFIER	NONE		-1	ENSSSCG00000012021-ssc-mir-155			-1
13	1.99E+08	A	G	2218.57	158	1/1	1/1	1/1	0/1	0/1	1/1	0/0	upstream_	MODIFIER	NONE	c.-38T>C	-1	ENSSSCG(	protein_coding	ENSSSCT0	-1
13	1.99E+08	A	G	2218.57	158	1/1	1/1	1/1	0/1	0/1	1/1	0/0	intergenic_	MODIFIER	NONE		-1	ENSSSCG00000012021-ssc-mir-155			-1
13	1.99E+08	A	G	2354.38	165	1/1	1/1	1/1	0/1	0/1	1/1	0/0	upstream_	MODIFIER	NONE	c.-38T>C	-1	ENSSSCG(	protein_coding	ENSSSCT0	-1
13	1.99E+08	A	G	2354.38	165	1/1	1/1	1/1	0/1	0/1	1/1	0/0	intergenic_	MODIFIER	NONE		-1	ENSSSCG00000012021-ssc-mir-155			-1
13	1.99E+08	G	A	2322.37	159	1/1	1/1	1/1	0/1	0/1	1/1	0/0	upstream_	MODIFIER	NONE	c.-38C>T	-1	ENSSSCG(	protein_coding	ENSSSCT0	-1
13	1.99E+08	G	A	2322.37	159	1/1	1/1	1/1	0/1	0/1	1/1	0/0	intergenic_	MODIFIER	NONE		-1	ENSSSCG00000012021-ssc-mir-155			-1
13	1.99E+08	G	A	2023.59	156	1/1	1/1	1/1	0/1	0/1	1/1	0/0	upstream_	MODIFIER	NONE	c.-38C>T	-1	ENSSSCG(	protein_coding	ENSSSCT0	-1
13	1.99E+08	G	A	2023.59	156	1/1	1/1	1/1	0/1	0/1	1/1	0/0	intergenic_	MODIFIER	NONE		-1	ENSSSCG00000012021-ssc-mir-155			-1
13	1.99E+08	G	A	1685.62	144	1/1	1/1	1/1	0/1	0/1	1/1	0/0	intergenic_	MODIFIER	NONE		-1	ENSSSCG00000012021-ssc-mir-155			-1
13	1.99E+08	T	C	1771.66	151	1/1	1/1	1/1	0/1	1/1	1/1	0/0	intergenic_	MODIFIER	NONE		-1	ssc-mir-155-JAM2			-1
13	2.05E+08	G	C	1377.22	130	1/1	1/1	1/1	1/1	1/1	1/1	0/0	intron_var	MODIFIER	NONE	c.1481-525C>G	-1	TIAM1	protein_coding	ENSSSCT0	10
13	2.05E+08	C	G	1075.28	120	1/1	1/1	1/1	1/1	1/1	1/1	0/0	intron_var	MODIFIER	NONE	c.1481-557G>C	-1	TIAM1	protein_coding	ENSSSCT0	10
13	2.06E+08	T	C	3723.55	176	1/1	1/1	1/1	1/1	1/1	1/1	0/0	intergenic_	MODIFIER	NONE		-1	SCAF4-U6			-1
13	2.06E+08	GC	G	6886.68	184	1/1	1/1	1/1	1/1	1/1	1/1	0/0	intergenic_	MODIFIER	NONE		-1	U6-HUNK			-1
13	2.06E+08	T	C	5664.55	218	1/1	1/1	1/1	1/1	1/1	1/1	0/0	intergenic_	MODIFIER	NONE		-1	U6-HUNK			-1
13	2.06E+08	TGCTGTG	T	7940.72	108	1/1	1/1	1/1	1/1	1/1	1/1	0/0	intergenic_	MODIFIER	NONE	n.null_nulldelGCTGTG	-1	U6-HUNK			-1
13	2.06E+08	A	G	6807.55	306	1/1	1/1	1/1	1/1	1/1	1/1	0/0	intergenic_	MODIFIER	NONE		-1	HUNK-BRSK2			-1
13	2.06E+08	A	AAAC	8022.04	175	1/1	1/1	1/1	1/1	1/1	1/1	0/0	intergenic_	MODIFIER	NONE	n.null_nullinsAAC	-1	BRSK2-EVA1C			-1
13	2.09E+08	A	C	1572.35	192	1/1	1/1	1/1	1/1	0/1	0/1	0/0	intergenic_	MODIFIER	NONE		-1	RUNX1-ENSSSCG00000027172			-1
14	11250816	G	T	1477.15	177	0/0	1/1	1/1	0/1	0/1	1/1	1/1	intergenic_	MODIFIER	NONE		-1	ENSSSCG00000009655-PPP2R2A			-1
14	1.5E+08	G	A	1674.14	215	0/0	0/1	0/1	0/1	0/1	0/1	1/1	intergenic_	MODIFIER	NONE		-1	5S_rRNA-MGMT			-1
14	9364596	C	G	1136.8	151	1/1	1/1	1/1	1/1	1/1	1/1	0/0	intergenic_	MODIFIER	NONE		-1	STC1-ADAM28			-1
14	11231630	C	T	1082.61	131	1/1	1/1	1/1	1/1	1/1	0/1	0/0	intergenic_	MODIFIER	NONE		-1	ENSSSCG00000009655-PPP2R2A			-1
14	12236242	A	AG	1187.77	220	1/1	0/1	0/1	0/1	0/1	1/1	0/0	upstream_	MODIFIER	NONE	c.-524_-524insC	-1	TRIM35	protein_coding	ENSSSCT0	-1
14	12236242	A	AG	1187.77	220	1/1	0/1	0/1	0/1	0/1	1/1	0/0	intergenic_	MODIFIER	NONE	n.null_nullinsG	-1	TRIM35-PTK2B			-1
14	13681570	TG	T	7991	231	1/1	1/1	1/1	1/1	1/1	1/1	0/0	intron_var	MODIFIER	NONE	c.1406+1198delG	-1	EXTL3	protein_coding	ENSSSCT0	5
14	17852170	T	C	1425.06	158	1/1	1/1	1/1	1/1	1/1	1/1	0/0	intergenic_	MODIFIER	NONE		-1	HAND2-ENSSSCG00000009704			-1
14	20424379	G	GT	4796.6	234	1/1	1/1	1/1	1/1	1/1	0/1	0/0	intergenic_	MODIFIER	NONE	n.null_nullinsT	-1	ENSSSCG00000024906-AADAT			-1
14	21859350	T	TA	3424.31	204	1/1	1/1	1/1	1/1	1/1	0/1	0/0	intergenic_	MODIFIER	NONE	n.null_nullinsA	-1	SH3RF1-CBR4			-1
14	24064797	G	A	6548.6	225	1/1	1/1	1/1	1/1	1/1	1/1	0/0	intron_var	MODIFIER	NONE	c.352+384G>A	-1	CHFR	protein_coding	ENSSSCT0	3
14	40188921	C	T	1557.9	156	1/1	1/1	1/1	1/1	1/1	0/1	0/0	intergenic_	MODIFIER	NONE		-1	ENSSSCG00000009866-TBX5			-1
14	40383540	C	T	2063.62	156	1/1	1/1	1/1	1/1	1/1	0/1	0/0	intergenic_	MODIFIER	NONE		-1	TBX5-U6			-1
14	40414279	C	T	2176.34	148	1/1	1/1	1/1	1/1	1/1	0/1	0/0	upstream_	MODIFIER	NONE	n.-1C>T	-1	U6	snRNA	ENSSSCT0	-1
14	40414279	C	T	2176.34	148	1/1	1/1	1/1	1/1	1/1	0/1	0/0	intergenic_	MODIFIER	NONE		-1	TBX5-U6			-1
14	71628311	C	CTT	2366.72	235	1/1	1/1	0/1	0/1	0/1	1/1	0/0	intergenic_	MODIFIER	NONE	n.null_nullinsTT	-1	EGR2-NRBF2			-1
14	72046301	C	CACAT	6619.88	265	1/1	1/1	1/1	0/1	0/1	0/1	0/0	intergenic_	MODIFIER	NONE	n.null_nullinsACAT	-1	JMJD1C-ssc-mir-1296			-1
14	72609770	A	AT	1430.63	261	1/1	1/1	1/1	0/0	0/0	1/1	0/0	intergenic_	MODIFIER	NONE	n.null_nullinsT	-1	ENSSSCG00000028393-CTNNA3			-1
14	73661476	CA	C	4225.41	256	1/1	1/1	1/1	0/1	0/1	1/1	0/0	intergenic_	MODIFIER	NONE		-1	ENSSSCG00000028393-CTNNA3			-1
14	74093522	T	C	1218.21	170	1/1	1/1	1/1	0/1	0/1	1/1	0/0	intergenic_	MODIFIER	NONE		-1	ENSSSCG00000028393-CTNNA3			-1
14	74598813	C	T	1653.67	160	1/1	1/1	1/1	0/1	0/1	1/1	0/0	intergenic_	MODIFIER	NONE		-1	ENSSSCG00000028393-CTNNA3			-1
14	99445928	T	A	1441.96	169	1/1	1/1	1/1	1/1	1/1	1/1	0/0	intergenic_	MODIFIER	NONE		-1	ENSSSCG00000029530-ENSSSCG00000023			-1
14	1.16E+08	T	G	1465.43	175	1/1	1/1	1/1	1/1	1/1	0/1	0/0	intron_var	MODIFIER	NONE	c.688+1823A>C	-1	ENSSSCG(	protein_coding	ENSSSCT0	9
14	1.2E+08	C	CT	3373.8	249	1/1	1/1	1/1	1/1	1/1	1/1	0/0	intron_var	MODIFIER	NONE	c.1533+403_1533+40	-1	ABCC2	protein_coding	ENSSSCT0	11
14	1.27E+08	A	G	1030.11	129	1/1	1/1	1/1	1/1	1/1	0/1	0/0	intergenic_	MODIFIER	NONE		-1	U8-U6			-1
14	1.3E+08	G	GAAAC	1385	226	1/1	1/1	1/1	1/1	1/1	1/1	0/0	intergenic_	MODIFIER	NONE	n.null_nullinsAAAC	-1	SNORA62-XPNPEP1			-1
14	1.34E+08	G	A	1407.89	165	1/1	1/1	1/1	1/1	1/1	0/1	0/0	intron_var	MODIFIER	NONE	c.797-63C>T	-1	ENSSSCG(	protein_coding	ENSSSCT0	9

14	1.34E+08	G	A	1033.17	157	1/1	1/1	1/1	1/1	1/1	0/1	0/0	intron_var	MODIFIER	NONE	c.797-90C>T	-1	ENSSSCG(	protein_coding	ENSSSCT0	9
15	19740207	C	A	1451.84	147	0/0	0/1	0/1	1/1	1/1	1/1	1/1	intergenic_	MODIFIER	NONE		-1	ENSSSCG00000029118-ENSSSCG00000015			-1
15	24436358	T	C	1285.4	173	0/0	0/1	0/1	1/1	1/1	1/1	1/1	intergenic_	MODIFIER	NONE		-1	U6-ENSSSCG00000026504			-1
15	38157850	T	TA	1519.66	225	0/0	0/1	0/1	1/1	1/1	1/1	1/1	intergenic_	MODIFIER	NONE	n.null_nullinsA	-1	ENSSSCG00000015747-ENSSSCG00000029			-1
15	65506863	G	A	1070.23	133	0/0	0/1	0/1	1/1	1/1	1/1	1/1	intergenic_	MODIFIER	NONE		-1	SNORA19-HS6ST1			-1
15	65506911	C	T	1551.78	156	0/0	0/1	0/1	1/1	1/1	1/1	1/1	intergenic_	MODIFIER	NONE		-1	SNORA19-HS6ST1			-1
15	1.09E+08	A	C	1425.75	160	0/0	0/1	0/1	1/1	1/1	1/1	1/1	intergenic_	MODIFIER	NONE		-1	TMEFF2-STK17B			-1
15	1.36E+08	G	T	1526.59	126	0/0	0/1	0/1	1/1	1/1	1/1	1/1	intergenic_	MODIFIER	NONE		-1	U6-SNORA31			-1
15	1.41E+08	C	CA	1924.64	229	0/0	0/1	0/1	0/1	0/1	1/1	1/1	intergenic_	MODIFIER	NONE	n.null_nullinsA	-1	U6-ENSSSCG00000030288			-1
15	1.51E+08	AC	A	2015.52	203	0/0	0/0	0/0	0/1	0/1	0/1	1/1	intergenic_	MODIFIER	NONE		-1	COPS8-COL6A3			-1
15	19382449	T	C	1183.19	148	1/1	1/1	1/1	1/1	1/1	1/1	0/0	upstream_	MODIFIER	NONE	c.-1T>C	-1	MAP3K19	protein_coding	ENSSSCT0	-1
15	19382449	T	C	1183.19	148	1/1	1/1	1/1	1/1	1/1	1/1	0/0	intergenic_	MODIFIER	NONE		-1	RAB3GAP1-MAP3K19			-1
15	19761538	T	G	1835.16	162	1/1	1/1	1/1	1/1	1/1	1/1	0/0	intergenic_	MODIFIER	NONE		-1	ENSSSCG00000029118-ENSSSCG00000015			-1
15	37952226	A	AAAT	2149.4	231	1/1	1/1	0/1	1/1	1/1	0/1	0/0	intron_var	MODIFIER	NONE	c.1139-1263_1139-12	-1	ENSSSCG(	protein_coding	ENSSSCT0	10
15	1.43E+08	A	G	1239.38	110	1/1	1/1	1/1	0/1	0/1	0/1	0/0	intergenic_	MODIFIER	NONE		-1	U6-DNER			-1
15	1.5E+08	A	C	1754.64	176	1/1	1/1	1/1	1/1	1/1	0/1	0/0	intergenic_	MODIFIER	NONE		-1	ENSSSCG00000024859-ENSSSCG00000016			-1
15	1.52E+08	TA	T	1704.25	190	1/1	1/1	1/1	0/1	0/1	0/0	0/0	upstream_	MODIFIER	NONE	c.-1delA	-1	ENSSSCG(	protein_coding	ENSSSCT0	-1
15	1.52E+08	TA	T	1704.25	190	1/1	1/1	1/1	0/1	0/1	0/0	0/0	intergenic_	MODIFIER	NONE		-1	KLHL30-ENSSSCG00000016337			-1
15	1.52E+08	A	G	1069.65	142	1/1	1/1	1/1	0/1	0/1	0/1	0/0	intergenic_	MODIFIER	NONE		-1	ENSSSCG00000016338-ENSSSCG00000018			-1
16	59844155	G	C	1032.56	119	0/0	0/1	0/0	1/1	0/1	0/1	1/1	intergenic_	MODIFIER	NONE		-1	SLIT3-ENSSSCG00000026810			-1
16	2989313	A	G	2092.88	100	1/1	1/1	1/1	0/1	0/1	1/1	0/0	intergenic_	MODIFIER	NONE		-1	U6-ENSSSCG00000029792			-1
16	13581850	CT	C	4961.18	235	1/1	1/1	1/1	1/1	1/1	1/1	0/0	intergenic_	MODIFIER	NONE		-1	ENSSSCG00000029525-CDH9			-1
16	15552122	G	GT	5370.87	254	1/1	1/1	1/1	1/1	1/1	1/1	0/0	intergenic_	MODIFIER	NONE	n.null_nullinsT	-1	ENSSSCG00000018139-U6			-1
16	23627177	G	A	1113.76	128	1/1	1/1	1/1	1/1	1/1	0/0	0/0	intergenic_	MODIFIER	NONE		-1	C5orf42-NUP155			-1
16	76876718	T	C	1501.23	146	1/1	1/1	1/1	0/1	0/1	0/1	0/0	intergenic_	MODIFIER	NONE		-1	NMUR2-ENSSSCG00000017077			-1
17	36984058	C	CT	1772.88	238	0/0	0/0	0/0	0/1	1/1	1/1	1/1	intergenic_	MODIFIER	NONE	n.null_nullinsT	-1	OXT-MRPS26			-1
17	40342407	C	T	1218.62	126	0/0	0/1	0/1	1/1	1/1	1/1	1/1	upstream_	MODIFIER	NONE	c.-1C>T	-1	ENSSSCG(	protein_coding	ENSSSCT0	-1
17	40342407	C	T	1218.62	126	0/0	0/1	0/1	1/1	1/1	1/1	1/1	intron_var	MODIFIER	NONE	c.-172+1139C>T	-1	DUSP15	protein_coding	ENSSSCT0	3
17	40342423	A	T	1394.48	134	0/0	0/1	0/1	1/1	1/1	1/1	1/1	upstream_	MODIFIER	NONE	c.-1A>T	-1	ENSSSCG(	protein_coding	ENSSSCT0	-1
17	40342423	A	T	1394.48	134	0/0	0/1	0/1	1/1	1/1	1/1	1/1	intron_var	MODIFIER	NONE	c.-172+1155A>T	-1	DUSP15	protein_coding	ENSSSCT0	3
17	40342441	T	G	1631.21	147	0/0	0/1	0/1	1/1	1/1	1/1	1/1	upstream_	MODIFIER	NONE	c.-1T>G	-1	ENSSSCG(	protein_coding	ENSSSCT0	-1
17	40342441	T	G	1631.21	147	0/0	0/1	0/1	1/1	1/1	1/1	1/1	intron_var	MODIFIER	NONE	c.-172+1173T>G	-1	DUSP15	protein_coding	ENSSSCT0	3
17	43895202	T	A	1201.82	145	0/0	0/1	0/1	1/1	1/1	1/1	1/1	intron_var	MODIFIER	NONE	c.24+870A>T	-1	ENSSSCG(	protein_coding	ENSSSCT0	1
17	265816	G	A	1388.63	126	1/1	1/1	1/1	1/1	1/1	1/1	0/0	intergenic_	MODIFIER	NONE		-1	ENSSSCG00000024717-RPS19			-1
17	283821	C	T	1053.28	142	1/1	1/1	1/1	0/1	0/1	1/1	0/0	intergenic_	MODIFIER	NONE		-1	ENSSSCG00000024717-RPS19			-1
17	46362451	G	A	6937.55	295	1/1	1/1	1/1	1/1	1/1	1/1	0/0	intron_var	MODIFIER	NONE	c.121+26649G>A	-1	ENSSSCG(	protein_coding	ENSSSCT0	1
17	46362451	G	A	6937.55	295	1/1	1/1	1/1	1/1	1/1	1/1	0/0	intron_var	MODIFIER	NONE	c.360-5065C>T	-1	ENSSSCG(	protein_coding	ENSSSCT0	5
17	51225602	C	T	1283.86	145	1/1	1/1	1/1	1/1	1/1	1/1	0/0	intergenic_	MODIFIER	NONE		-1	U6-SRSF6			-1
17	52157271	C	T	1387.06	119	1/1	1/1	1/1	1/1	1/1	1/1	0/0	upstream_	MODIFIER	NONE	c.-1C>T	-1	GDAP1L1	protein_coding	ENSSSCT0	-1
17	52157271	C	T	1387.06	119	1/1	1/1	1/1	1/1	1/1	1/1	0/0	intergenic_	MODIFIER	NONE		-1	ENSSSCG00000025757-GDAP1L1			-1
17	52157272	A	G	1304.06	117	1/1	1/1	1/1	1/1	1/1	1/1	0/0	upstream_	MODIFIER	NONE	c.-1A>G	-1	GDAP1L1	protein_coding	ENSSSCT0	-1
17	52157272	A	G	1304.06	117	1/1	1/1	1/1	1/1	1/1	1/1	0/0	intergenic_	MODIFIER	NONE		-1	ENSSSCG00000025757-GDAP1L1			-1
17	53285059	C	A	1058.83	141	1/1	1/1	1/1	1/1	1/1	1/1	0/0	upstream_	MODIFIER	NONE	c.-292G>T	-1	ENSSSCG(	protein_coding	ENSSSCT0	-1
17	53285059	C	A	1058.83	141	1/1	1/1	1/1	1/1	1/1	1/1	0/0	intron_var	MODIFIER	NONE	c.255-6922G>T	-1	ENSSSCG(	protein_coding	ENSSSCT0	3
17	53560143	C	A	1164.32	104	1/1	1/1	1/1	1/1	1/1	1/1	0/0	intergenic_	MODIFIER	NONE		-1	ENSSSCG00000024548-SPINT4			-1
17	64717988	A	AT	1614.27	239	1/1	1/1	1/1	1/1	1/1	0/1	0/0	intergenic_	MODIFIER	NONE	n.null_nullinsT	-1	U6-BMP7			-1
17	67813563	C	T	1257.37	138	1/1	1/1	1/1	1/1	1/1	1/1	0/0	intergenic_	MODIFIER	NONE		-1	ENSSSCG00000020676-ENSSSCG00000026			-1
17	68303209	A	G	1365.64	137	1/1	1/1	1/1	1/1	1/1	0/1	0/0	intergenic_	MODIFIER	NONE		-1	ENSSSCG00000026743-ENSSSCG00000007			-1
18	46377954	A	G	1019.53	97	0/0	0/1	0/1	1/1	1/1	1/1	1/1	intergenic_	MODIFIER	NONE		-1	PAC1-GHRHR			-1
18	55245294	T	C	1405.56	122	0/0	0/1	1/1	0/0	0/1	1/1	1/1	intron_var	MODIFIER	NONE	c.273+8194A>G	-1	CCM2	protein_coding	ENSSSCT0	3
18	23831	A	C	1083.47	125	1/1	1/1	1/1	1/1	1/1	1/1	0/0	intron_var	MODIFIER	NONE	c.1634-1153A>C	-1	SARM1	protein_coding	ENSSSCT0	6
18	4708539	T	A	1445.3	126	1/1	1/1	1/1	1/1	1/1	1/1	0/0	intergenic_	MODIFIER	NONE		-1	ENSSSCG00000016424-XRCC2			-1
18	4975630	A	G	1546.02	132	1/1	1/1	1/1	0/1	0/1	1/1	0/0	intron_var	MODIFIER	NONE	c.39+38218A>G	-1	XRCC2	protein_coding	ENSSSCT0	1
18	4975630	A	G	1546.02	132	1/1	1/1	1/1	0/1	0/1	1/1	0/0	intron_var	MODIFIER	NONE	c.304-3153A>G	-1	ENSSSCG(	protein_coding	ENSSSCT0	3
18	9872906	C	T	4686.15	265	1/1	1/1	1/1	0/1	0/1	1/1	0/0	intergenic_	MODIFIER	NONE		-1	MKRN1-RAB19			-1
18	12561470	C	CA	2329.42	204	1/1	1/1	1/1	1/1	1/1	0/1	0/0	intergenic_	MODIFIER	NONE	n.null_nullinsA	-1	ENSSSCG00000022517-ENSSSCG00000025			-1
18	14272478	C	T	4476.21	201	1/1	1/1	1/1	0/1	0/1	1/1	0/0	intergenic_	MODIFIER	NONE		-1	ssc-mir-490-2-FAM180A			-1
18	14272507	A	G	4961.18	217	1/1	1/1	1/1	0/1	0/1	1/1	0/0	intergenic_	MODIFIER	NONE		-1	ssc-mir-490-2-FAM180A			-1
18	14290635	C	T	3422.16	227	1/1	1/1	1/1	0/1	0/1	1/1	0/0	intergenic_	MODIFIER	NONE		-1	ssc-mir-490-2-FAM180A			-1
18	22392605	G	A	1838.99	121	1/1	1/1	1/1	1/1	1/1	1/1	0/0	intergenic_	MODIFIER	NONE		-1	ENSSSCG00000022443-ENSSSCG00000025			-1
18	37847421	G	GT	4164.56	224	1/1	1/1	1/1	1/1	1/1	0/1	0/0	intergenic_	MODIFIER	NONE	n.null_nullinsT	-1	LRRN3-ENSSSCG00000026444			-1
18	42752356	GTA	G	5235.66	268	1/1	1/1	1/1	1/1	1/1	0/1	0/0	intergenic_	MODIFIER	NONE	n.null_nulldelTA	-1	DPY19L1-NPSR1			-1
18	46136656	T	TA	3959.55	221	1/1	1/1	1/1	1/1	1/1	1/1	0/0	intergenic_	MODIFIER	NONE	n.null_nullinsA	-1	PPP1R17-PAC1			-1
18	49533032	G	A	1031.01	159	1/1	1/1	1/1	1/1	1/1	1/1	0/0	upstream_	MODIFIER	NONE	n.-1C>T	-1	U6	snRNA	ENSSSCT0	-1
18	49533032	G	A	1031.01	159	1/1	1/1	1/1	1/1	1/1	1/1	0/0	intergenic_	MODIFIER	NONE		-1	U6-ENSSSCG00000016695			-1
18	51188236	T	C	1015.59	130	1/1	./.	1/1	1/1	1/1	1/1	0/0	intergenic_	MODIFIER	NONE		-1	NFE2L3-ssc-mir-148a			-1
18	53721711	CTTT	C	3183.71	206	1/1	1/1	1/1	0/1	1/1	0/1	0/0	intron_var	MODIFIER	NONE	c.-115+5088_-115+5(	-1	TNS3	protein_coding	ENSSSCT0	3

18	55489108	G	A	5875.09	244	1/1	1/1	1/1	1/1	1/1	0/1	0/0	intron_vari	MODIFIER	NONE	c.1712-19853G>A	-1	ENSSSCG(	protein_coding	ENSSSCT0	11
18	55489108	G	A	5875.09	244	1/1	1/1	1/1	1/1	1/1	0/1	0/0	intron_vari	MODIFIER	NONE	c.-418-2427C>T	-1	ENSSSCG(	protein_coding	ENSSSCT0	1
18	55798214	C	G	6902.53	269	1/1	1/1	1/1	1/1	1/1	0/1	0/0	intergenic_	MODIFIER	NONE		-1	NUDCD3-ENSSSCG00000028116			-1
18	56331394	CT	C	4487.71	214	1/1	1/1	1/1	1/1	1/1	1/1	0/0	intergenic_	MODIFIER	NONE		-1	5S_rRNA-HECW1			-1
18	57284292	C	T	6788.53	337	1/1	1/1	1/1	1/1	1/1	0/1	0/0	intergenic_	MODIFIER	NONE		-1	C7orf25-ENSSSCG00000016762			-1
18	58205306	CTT	C	3833.6	155	1/1	1/1	1/1	1/1	1/1	0/1	0/0	intergenic_	MODIFIER	NONE	n.null_nulldelTT	-1	INHBA-ENSSSCG00000016765			-1
18	58858477	A	G	1087.65	138	1/1	1/1	1/1	1/1	1/1	1/1	0/0	intergenic_	MODIFIER	NONE		-1	ENSSSCG00000016766-SUGCT			-1
18	58858485	C	T	1176.08	140	1/1	1/1	1/1	1/1	1/1	1/1	0/0	intergenic_	MODIFIER	NONE		-1	ENSSSCG00000016766-SUGCT			-1
18	59078489	C	G	6684.53	307	1/1	1/1	1/1	1/1	1/1	0/1	0/0	intergenic_	MODIFIER	NONE		-1	ENSSSCG00000016766-SUGCT			-1
18	59720279	T	C	4829.54	255	1/1	1/1	1/1	1/1	1/1	0/1	0/0	intergenic_	MODIFIER	NONE		-1	CDK13-U6			-1
X	6727207	TGC	T	3333.81	261	0/0	0/0	0/0	0/1	0/1	1/1	1/1	intron_vari	MODIFIER	NONE	c.317+3568_317+356	-1	SHROOM2	protein_coding	ENSSSCT0	2
X	7195511	T	G	2281.44	201	0/0	0/0	0/0	0/1	0/1	1/1	1/1	intron_vari	MODIFIER	NONE	n.634+28971T>G	-1	WWC3	processed_transcript	ENSSSCT0	1
X	16072712	G	A	2807.39	231	0/0	0/0	0/0	0/1	0/1	1/1	1/1	intron_vari	MODIFIER	NONE	c.-166+29841G>A	-1	CDKL5	protein_coding	ENSSSCT0	1
X	16832884	G	T	2050.16	215	0/0	0/0	0/0	0/1	0/1	0/0	1/1	intergenic_	MODIFIER	NONE		-1	GPR64-CH242-78D20.1			-1
X	1.26E+08	A	G	3689.76	221	0/0	0/1	0/0	0/1	0/1	1/1	1/1	intron_vari	MODIFIER	NONE	c.1163+5756T>C	-1	GPC3	protein_coding	ENSSSCT0	4
X	1.29E+08	T	A	2958.38	334	0/0	0/0	0/0	0/1	0/1	1/1	1/1	intergenic_	MODIFIER	NONE		-1	ZIC3-CH242-204G11.1			-1
X	1.29E+08	T	C	1319.7	177	0/0	0/0	0/0	0/1	0/1	1/1	1/1	intergenic_	MODIFIER	NONE		-1	ZIC3-CH242-204G11.1			-1
X	1.31E+08	C	T	2758.79	236	0/0	0/1	0/1	0/1	0/1	1/1	1/1	intron_vari	MODIFIER	NONE	c.435+4062G>A	-1	ATP11C	protein_coding	ENSSSCT0	5
X	4246456	G	A	5920.53	213	1/1	1/1	1/1	1/1	1/1	0/1	0/0	intron_vari	MODIFIER	NONE	c.1366+4636G>A	-1	STS	protein_coding	ENSSSCT0	10
X	39984625	G	A	2897.52	302	1/1	1/1	1/1	0/1	0/1	0/0	0/0	intergenic_	MODIFIER	NONE		-1	BCOR-U6			-1
X	1.11E+08	A	C	2480.36	183	1/1	1/1	1/1	0/1	0/1	0/1	0/0	intergenic_	MODIFIER	NONE		-1	U6-CH242-79N16.1			-1
X	1.26E+08	G	GT	3364.37	147	1/1	1/1	1/1	1/1	1/1	0/1	0/0	intergenic_	MODIFIER	NONE	n.null_nullinsT	-1	GPC4-GPC3			-1
