# Supplementary material for: No excessive mutations in transcription activator-like effector nuclease-mediated α-1,3-galactosyltransferase knockout Yucatan miniature pigs
Source: Asian-Australas J Anim Sci. 2019 Aug 23;33(2):360–72. doi: 10.5713/ajas.19.0480 (PMC6946973; doi:10.5713/ajas.19.0480)
Supplement: Supplementary file 7 [file ajas-19-0480-suppl7.pdf]

Supplementary Table 7. Differential expressed genes between TALEN-edited pigs and wild type strain control

Order	Tran	TranAcc	GeneName	Chr	Start	End	Strand	Type	Status	Desc	Num_of_Samples	EXP:CJ1:FPKM	EXP:CB1:FPKM	EXP:CB3:FPKM	EXP:WT:FPKM	CJ1/WT	CB1/WT	CB3/WT	Rseq-WGS (TranAcc)	Rseq-WGS (GeneName)
12851	TBIG01285	ENSSSCG0	MS4A8B	2	10572887	10573067	-	CODING	KNOWN	Uncharacte	7	993.712	2175.65	169.184	6486.59	-6.52764	-2.98145	-38.3404	-	-
25850	TBIG02585	XLOC_025	Brf1	GL892627.	61598	64554	+	CODING	NOVEL	Transcripti	7	5844.93	5147.78	596.764	14674.7	-2.51067	-2.85069	-24.5905	-	-
21241	TBIG02124	ENSSSCG0	SLA-5	7	24643495	24685566	+	CODING	NOVEL	Uncharacte	7	2469.65	1117.89	633.824	13588.6	-5.50224	-12.1556	-21.4391	-	-
26453	TBIG02645	XLOC_026	ALAD	GL893446.	1	256	+	CODING	NOVEL	Delta-amir	7	18092.1	11315.1	4975.5	94831.2	-5.24158	-8.38094	-19.0596	-	-
28902	TBIG02890	XLOC_028	Mark4	GL896419.	25788	25980	.	CODING	NOVEL	MAP/micro	7	1224.94	2334.19	543.951	9744.39	-7.95499	-4.17463	-17.9141	-	-
26029	TBIG02602	XLOC_026	Lrrc61	GL892911.	272446	272602	.	CODING	NOVEL	Leucine-ric	7	1343.4	12552.7	3278.29	52643.9	-39.1871	-4.19383	-16.0583	-	-
7248	TBIG00724	ENSSSCG0	USMG5	14	50545515	50545776	+	CODING	KNOWN	up-regulat	7	1735.83	4383.96	940.426	13295	-7.65916	-3.03265	-14.1372	-	-
28708	TBIG02870	XLOC_028	IL3RA	GL896347.	6795	7293	-	CODING	NOVEL	Interleukin	7	343.757	535.83	148.954	1996.01	-5.80646	-3.72508	-13.4002	-	-
3163	TBIG00316	XLOC_003	plxdc2	10	60033512	60033722	.	CODING	NOVEL	Plexin dom	7	466.216	413.47	89.2053	1100.18	-2.35981	-2.66085	-12.3331	-	-
26519	TBIG02651	XLOC_026	IL10RB	GL893534.	34	249	.	CODING	NOVEL	Interleukin	7	444.221	897.454	178.531	2135.31	-4.80686	-2.3793	-11.9604	-	-
1358	TBIG00135	ENSSSCG0	CSNK1G1	1	1.19E+08	1.19E+08	-	CODING	KNOWN	Uncharacte	7	185.684	28.1274	63.8625	621.448	-3.3468	-22.094	-9.73103	-	-
27071	TBIG02707	XLOC_027	Klc3	GL894198.	17666	17856	.	CODING	NOVEL	Kinesin ligl	7	1785.26	2045.74	876.138	7911.74	-4.4317	-3.86742	-9.03024	-	-
26368	TBIG02636	XLOC_026	PLEKHH1	GL893315.	20220	20387	.	CODING	NOVEL	Pleckstrin	7	1024.25	807.283	452.041	4043	-3.94728	-5.00816	-8.94388	-	-
25530	TBIG02553	XLOC_025	Dnm3	9	1.26E+08	1.26E+08	.	CODING	NOVEL	Dynamin-	7	236.256	93.8751	183.984	1641.89	-6.94962	-17.4902	-8.92409	-	-
3726	TBIG00372	XLOC_003	VWA8	11	26081870	26082015	.	CODING	NOVEL	von Willeb	7	9889.72	1107.1	556.956	4939.05	2.002353	-4.46125	-8.86794	-	-
28459	TBIG02845	XLOC_028	YBEY	GL896243.	11753	13386	-	CODING	NOVEL	Putative ril	7	11671.8	9993.06	3216.59	28354.6	-2.42933	-2.83743	-8.81511	-	-
3103	TBIG00310	XLOC_003	Rpl7	10	38814256	38814448	.	CODING	NOVEL	60S riboso	7	36830	28929.8	10280.7	83910	-2.27831	-2.90047	-8.1619	-	-
28677	TBIG02867	XLOC_028	UBAC1	GL896332.	34809	35168	-	CODING	NOVEL	Ubiquitin-	7	896.056	487.796	387.436	3118.89	-3.48069	-6.39384	-8.05008	-	-
26632	TBIG02663	XLOC_026	ALAD	GL893668.	1	205	.	CODING	NOVEL	Delta-amir	7	508.509	213.068	144.345	1032.23	-2.02991	-4.8446	-7.15113	-	-
29618	TBIG02961	ENSSSCG0	Inmt	JH118806.	24194	29094	+	CODING	NEW_AS	Uncharacte	7	51.2178	12.9122	19.7954	131.443	-2.56635	-10.1798	-6.64008	-	-
28310	TBIG02831	XLOC_028	Aspscr1	GL896186.	4539	5283	-	CODING	NOVEL	Tether con	7	111101	427287	133790	887772	-7.99068	-2.07769	-6.63556	-	-
28755	TBIG02875	XLOC_028	SLC2A4RG	GL896364.	17152	17420	-	CODING	NOVEL	SLC2A4 re	7	529.225	1680.01	684.833	4442.35	-8.39407	-2.64424	-6.48676	-	-
28760	TBIG02876	XLOC_028	RTEL1	GL896364.	44051	44631	-	CODING	NOVEL	Regulator	7	2544.08	5045.32	2144.24	13675.2	-5.3753	-2.71047	-6.37764	-	-
10113	TBIG01011	XLOC_010	NDUFAF2	16	42680451	42680654	.	CODING	NOVEL	Mimitin, m	7	1021.82	566.254	447.605	2677.44	-2.62027	-4.72834	-5.9817	-	-
29136	TBIG02913	XLOC_029	MUCM_RA	GL896545.	5	1258	-	CODING	NOVEL	Ig mu chai	7	1902.74	3482.87	2616.84	15150.9	-7.96267	-4.35012	-5.78977	-	-
26170	TBIG02617	XLOC_026	FCGR3	GL893101.	2	233	.	CODING	NOVEL	Low affinit	7	21882.7	20977.6	10363.2	54871.7	-2.50754	-2.61573	-5.29486	-	-
23022	TBIG02302	XLOC_023	SORCS2	8	3950581	3957905	+	CODING	NOVEL	VPS10 dor	6	141.638	30.0105	109.574	565.652	-3.99365	-18.8485	-5.16228	-	-
7003	TBIG00700	XLOC_007	Pde9a	13	2.16E+08	2.16E+08	.	CODING	NOVEL	High affini	7	6734.99	10161	6118.63	31554.9	-4.68522	-3.10549	-5.15718	-	-
28498	TBIG02849	XLOC_028	ANAPC2	GL896260.	5047	5903	-	CODING	NOVEL	Anaphase-	7	5632.6	4455.65	2653.28	13344.2	-2.3691	-2.99489	-5.02932	-	-
28944	TBIG02894	ENSSSCG0	LSM2	GL896442.	6429	6878	-	CODING	KNOWN	Sus scrofa	7	2764.95	2160.36	1509.04	7535.84	-2.72549	-3.48823	-4.9938	-	-
2436	TBIG00243	XLOC_002	SHB	1	2.67E+08	2.67E+08	.	CODING	NOVEL	SH2 doma	7	458.985	203.963	249.52	1207.42	-2.63063	-5.9198	-4.83897	-	-
7176	TBIG00717	ENSSSCG0	PLBD2	14	40832763	40853776	+	CODING	NEW_AS	phospholip	7	290.154	277.52	25.5526	114.423	2.535801	2.425387	-4.47794	-	-
11163	TBIG01116	ENSSSCG0	Inmt	18	46637157	46641921	+	CODING	KNOWN	Indolethyla	7	86.2352	30.2503	70.6645	315.744	-3.66143	-10.4377	-4.46821	-	-
28832	TBIG02883	XLOC_028	Id3	GL896386.	13540	13725	.	CODING	NOVEL	DNA-bindi	7	3118.86	7254.2	12690.4	53577.3	-17.1785	-7.38569	-4.22188	-	-
21952	TBIG02195	ENSSSCG0	TMP-CH24	7	24706220	24722563	-	CODING	NOVEL	-	7	44834.2	26013.1	30648.3	126113	-2.81287	-4.84806	-4.11484	-	-
25078	TBIG02507	ENSSSCG0	STEAP4	9	74981686	75048318	-	CODING	NEW_AS	Sus scrofa	7	534.424	630.743	55.8317	227.229	2.351918	2.775803	-4.06989	-	-
12695	TBIG01269	XLOC_012	gag-pol	2	742220	747473	-	CODING	NOVEL	Gag-Pol p	7	28.239	18.9851	16.1817	64.6756	-2.29029	-3.40665	-3.99684	-	-
28985	TBIG02898	ENSSSCG0	CCDC137	GL896467.	17073	19084	+	CODING	NOVEL	coiled-coil	7	45.3546	21.9816	27.73	107.236	-2.36439	-4.87844	-3.86715	-	-
9846	TBIG00984	XLOC_009	Lilrb3	16	86105298	86420144	+	CODING	NEW_AS	Leukocyte	7	15.815	18.5381	10.4448	38.1865	-2.41457	-2.05989	-3.65603	-	-
11666	TBIG01166	ENSSSCG0	Polr2l	2	142435	143584	+	CODING	KNOWN	Uncharacte	7	434.073	383.895	252.755	912.007	-2.10105	-2.37567	-3.60826	-	-
28298	TBIG02829	XLOC_028	CKB	GL896176.	1	516	-	CODING	NOVEL	Creatine ki	7	8803.22	15344.7	10522.1	37111.1	-4.21563	-2.4185	-3.52697	-	-
26282	TBIG02628	XLOC_026	TMEM50A	GL893200.	4	247	.	CODING	NOVEL	Transmem	7	250.389	125.967	197.585	687.195	-2.74451	-5.45536	-3.47797	-	-
14008	TBIG01400	XLOC_014	skp1	2	1.46E+08	1.46E+08	.	CODING	NOVEL	S-phase ki	7	495282	508346	388191	1309070	-2.64308	-2.57516	-3.37223	-	-
28427	TBIG02842	XLOC_028	Notch1	GL896225.	29279	29470	.	CODING	NOVEL	Neurogeni	7	341.54	4103.8	3043.43	9855.52	-28.8561	-2.40156	-3.23829	-	-
25707	TBIG02570	XLOC_025	ALAD	GL892357.	1	153	.	CODING	NOVEL	Delta-amir	7	4997.38	7484.45	4885.37	15294	-3.0604	-2.04344	-3.13057	-	-
29719	TBIG02971	XLOC_029	DHODH	JH118898.	117287	117660	-	CODING	NOVEL	Dihydroor	7	19.9777	39.4171	48.8822	152.807	-7.64888	-3.87667	-3.12603	-	-
11812	TBIG01181	ENSSSCG0	FTH1	2	8983277	8986090	+	CODING	KNOWN	Sus scrofa	7	2699.56	2302.27	1858.38	5776.74	-2.13988	-2.50915	-3.10848	-	-
26017	TBIG02601	XLOC_026	Atp6v0e2	GL892911.	249005	252536	+	CODING	NOVEL	V-type pro	7	1802.02	912.166	1340.96	4077.25	-2.2626	-4.46986	-3.04055	-	-
8245	TBIG00824	ENSSSCG0	UROS	14	1.47E+08	1.47E+08	-	CODING	NEW_AS	uroporphy	7	92.8593	37.3737	68.2353	203.717	-2.19382	-5.45081	-2.98551	-	-
28476	TBIG02847	XLOC_028	OGFR	GL896248.	17478	17712	.	CODING	NOVEL	Opioid gro	7	64.4559	424.353	320.956	945.201	-14.6643	-2.22739	-2.94496	-	-
14314	TBIG01431	ENSSSCG0	HBM	3	40948175	40948852	+	CODING	KNOWN	hemoglobi	7	123.249	1175.84	4474.71	13125.1	-106.493	-11.1623	-2.93317	-	-
18556	TBIG01855	XLOC_018	TPT1	5	16183353	16183667	.	CODING	NOVEL	Translation	7	4311.82	3349.63	4702.8	13772.9	-3.19422	-4.11177	-2.92866	-	-
18253	TBIG01825	ENSSSCG0	Itpr2	5	51068458	51115752	-	CODING	NEW_AS	Uncharacte	7	394.53	253.902	409.283	1196.59	-3.03295	-4.7128	-2.92362	-	-
14478	TBIG01447	ENSSSCG0	RNF181	3	62044672	62046534	+	CODING	NEW_AS	Uncharacte	7	12.0065	14.9899	11.8412	33.6444	-2.80218	-2.24447	-2.8413	-	-
28284	TBIG02828	XLOC_028	NAPRT1	GL896158.	3	235	.	CODING	NOVEL	Nicotinate	7	357.39	511.042	690.025	1916.85	-5.36347	-3.75087	-2.77794	-	-
19785	TBIG01978	ENSSSCG0	KARS	6	11913750	11914466	-	CODING	KNOWN	Uncharacte	7	73.3252	74.4938	77.6953	213.652	-2.91376	-2.86805	-2.74987	-	-
26332	TBIG02633	XLOC_026	Ube2m	GL893268.	2	136	.	CODING	NOVEL	NEDD8-co	7	6588.11	41615.5	5228.5	14337.8	-2.17631	2.902502	-2.74224	-	-
23878	TBIG02387	XLOC_023	Pol	8	64377465	64377626	.	CODING	NOVEL	Retrovirus-	6	3454.52	3828.55	411.104	1080.51	3.19712	3.54328	-2.62831	-	-
29556	TBIG02955	ENSSSCG0	C18orf8	JH118750.	12529	14226	-	CODING	KNOWN	Uncharacte	7	200.381	236.421	222.718	581.38	-2.90137	-2.45909	-2.61039	-	-
21950	TBIG02195	ENSSSCG0	SLA-5	7	24684421	24749212	-	CODING	NOVEL	Uncharacte	7	25.0326	22.7202	24.6405	64.2552	-2.56686	-2.82811	-2.60771	-	-
24971	TBIG02497	ENSSSCG0	SORL1	9	53829400	53908876	-	CODING	NEW_AS	Uncharacte	7	128.003	94.3783	14.1043	36.4657	3.51023	2.588139	-2.58543	-	-
4391	TBIG00439	ENSSSCG0	SMIM5	12	5699681	5705158	-	CODING	NEW_AS	small integ	7	24.0128	122.285	133.317	331.307	-13.7971	-2.7093	-2.48511	-	-
8699	TBIG00869	ENSSSCG0	CIR1	15	89532289	89536128	+	CODING	KNOWN	Corepresso	7	29.1863	25.638	47.9882	116.686	-3.99797	-4.55129	-2.43156	-	-
28792	TBIG02879	ENSSSCG0	C3AR1	GL896371.	20699	29534	+	CODING	NEW_AS	compleme	7	60.146	99.9426	10.6256	25.78	2.333049	3.876749	-2.42622	-	-
1903	TBIG00190	ENSSSCG0	ENG	1	3.02E+08	3.02E+08	-	CODING	NEW_AS	endoglin [	7	18.1697	15.7716	16.0796	38.9832	-2.14551	-2.47173	-2.42439	-	-
19979	TBIG01997	XLOC_019	BLVRB	6	44457026	44477140	-	CODING	NEW_AS	Flavin redu	7	160.884	109.162	233.828	555.721	-3.45417	-5.09079	-2.37662	-	-
8469	TBIG00846	XLOC_008	RRP12	14	1.18E+08	1.18E+08	.	CODING	NOVEL	RRP12-like	7	323.053	222.757	349.659	828.666	-2.56511	-3.72004	-2.36993	-	-
14287	TBIG01428	ENSSSCG0	UBALD1	3	38812335	38818560	+	CODING	NEW_AS	UBA-like d	7	15.6868	26.6676	49.1655	116.277	-7.41241	-4.36023	-2.36501	-	-
14826	TBIG01482	ENSSSCG0	ZCWPW1	3	7661579	7699029	-	CODING	NEW_AS	zinc finger	7	131.417	110.706	17.9181	42.1027	3.121344	2.629428	-2.34973	-	-
18598	TBIG01859	XLOC_018	gag-pol	5	32350148	32350387	.	CODING	NOVEL	Gag-Pol p	7	68.9601	33.943	67.8701	157.659	-2.28624	-4.64482	-2.32295	-	-
27805	TBIG02780	XLOC_027	ALAD	GL895334.	77	900	+	CODING	NOVEL	Delta-amir	7	162.623	84.0241	164.766	379.589	-2.33417	-4.51762	-2.30381	-	-
19216	TBIG01921	XLOC_019	PPP6R1	6	54395348	54421714	+	CODING	NEW_AS	Serine/thre	7	44.7303	63.9774	100.722	228.415	-5.10649	-3.57025	-2.26778	-	-
15033	TBIG01503	XLOC_015	TMEM8A	3	41094370	41108203	-	CODING	NEW_AS	Transmem	7	37.2642	51.6734	51.141	114.589	-3.07504	-2.21756	-2.24065	-	-
22282	TBIG02228	ENSSSCG0	UBL7	7	63686694	63700113	-	CODING	NEW_AS	Ubiquitin-l	7	18.509	21.5286	19.7632	43.7621	-2.36437	-2.03274	-2.21432	-	-
2871	TBIG00287	ENSSSCG0	TNNI1	10	28197734	28209956	-	CODING	NEW_AS	troponin I	7	70.2778	25.4848	122.434	266.794	-3.79628	-10.4688	-2.17908	-	-
22340	TBIG02234	ENSSSCG0	DHRS4	7	80512359	80525829	-	CODING	NEW_AS	Sus scrofa	7	79.9157	55.3596	89.7143	194.499	-2.4338	-3.51337	-2.16798	-	-
1084	TBIG00108	ENSSSCG0	SYTL3	1	10432694	10496126	-	CODING	NEW_AS	synaptota	7	22.9497	24.5132	36.7202	79.4562	-3.46219	-3.24136	-2.16383	-	-
3607	TBIG00360	ENSSSCG0	ATP4B	11	86513343	86518522	-	CODING	NEW_AS	Sus scrofa	7	26.0453	11.1356	27.7599	59.9243	-2.30077	-5.38133	-2.15866	-	-
24928	TBIG02492	ENSSSCG0	REXO2	9	46875564	46901646	-	CODING	NEW_AS	RNA exon	7	529.207	117.972	499.004	1076.55	-2.03427	-9.12547	-2.1574	-	-
29174	TBIG02917	ENSSSCG0	S100A6	JH118413.	7263	15145	-	CODING	NEW_AS	S100 calci	7	10461.1	7490.73	10287.9	22149	-2.11727	-2.95685	-2.15292	-	-
13178	TBIG01317	ENSSSCG0	PRDX2	2	66509636	66513280	-	CODING	KNOWN	peroxiredo	7	849.569	426.978	820.643	1730.73	-2.03719	-4.05344	-2.10899	-	-
15022	TBIG01502	ENSSSCG0	TCEB2	3	40632308	40636179	-	CODING	NEW_AS	transcriptic	7	254.561	395.576	382.365	794.852	-3.12244	-2.00935	-2.07878	-	-
2924	TBIG00292	ENSSSCG0	SPINK4	10	37503364	37512778	-	CODING	KNOWN	serine pep	7	281.989	83.7321	312.814	645.369	-2.28863	-7.70755	-2.06311	-	-
7481	TBIG00748	ENSSSCG0	IFIT2	14	1.1E+08	1.1E+08	+	CODING	NEW_AS	interferon-	7	384.09	461.912	56.4053	116.176	3.306105	3.975967	-2.05966	-	-
21440	TBIG02144	ENSSSCG0	CRISP3	7	50187971	50199180	+	CODING	NOVEL	cysteine-ri	7	133.94	37.1248	218.708	449.226	-3.35393	-12.1004	-2.054	-	-
28269	TBIG02826	ENSSSCG0	CCS	GL896152.	70909	82133	-	CODING	NEW_AS	Copper ch	7	32.8154	16.0419	41.857	85.874	-2.61688	-5.35311	-2.0516	-	-
2634	TBIG00263	ENSSSCG0	ISCA1	10	33107717	33123610	+	CODING	NEW_AS	iron-sulfur	7	272.106	85.7237	381.292	781.335	-2.87144	-9.11457	-2.04918	-	-
28759	TBIG02875	XLOC_028	RTEL1	GL896364.	40477	42337	-	CODING	NOVEL	Regulator	7	83.392	121.713	231.428	474	-5.684	-3.89441	-2.04815	-	-
11089	TBIG01108	XLOC_011	BPGM	18	15416420	15419032	+	CODING	NOVEL	Bisphosph	7	13.8695	12.4585	34.0701	68.8939	-4.9673	-5.52987	-2.02212	-	-
28364	TBIG02836	XLOC_028	RAI1	GL896203.	14061	14193	.	CODING	NOVEL	Retinoic ac	7	7578.12	3082.9	10861.3	21840.3	-2.88202	-7.08434	-2.01084	-	-
28694	TBIG02869	ENSSSCG0	FIS1	GL896338.	43698	47176	-	CODING	KNOWN	fission 1 (r	7	202.085	131.694	213.269	426.678	-2.11138	-3.23992	-2.00066	-	-
12857	TBIG01285	ENSSSCG0	MS4A2	2	10959670	10979661	-	CODING	KNOWN	membrane	7	77.424	68.0864	51.2438	25.3795	3.050651	2.682732	2.019102	-	-
16276	TBIG01627	ENSSSCG0	IL6R	4	1.04E+08	1.04E+08	+	CODING	KNOWN	Uncharacte	7	66.3547	48.778	38.4394	18.8905	3.512596	2.582144	2.034853	-	-
10406	TBIG01040	ENSSSCG0	TOP1	17	49135521	49194449	+	CODING	NEW_AS	topoisome	7	60.7374	61.8106	46.6505	22.8897	2.653482	2.700367	2.038056	-	-
24345	TBIG02434	ENSSSCG0	FLI1	9	61714991	61812938	+	CODING	NEW_AS	Fli-1 proto	7	41.0179	27.3612	27.1468	13.0927	3.132883	2.089806	2.07343	-	-
10533	TBIG01053	ENSSSCG0	ASAH1	17	6296100	6334435	-	CODING	NEW_AS	N-acylsphi	7	47.7563	38.5345	38.6066	18.5625	2.57273	2.075933	2.079817	-	-
8623	TBIG00862	ENSSSCG0	SARAF	15	62330409	62349565	+	CODING	KNOWN	store-oper	7	51.1853	51.9449	47.2794	22.5594	2.268912	2.302583	2.095774	-	-
15173	TBIG01517	XLOC_015	KACB_RAT	3	59720334	59890986	-	CODING	NEW_AS	Ig kappa c	7	2076.73	4167.65	1570.06	743.986	2.791356	5.601786	2.110335	-	-
14992	TBIG01499	XLOC_014	Ubn1	3	38622375	38625658	-	CODING	NOVEL	Ubinuclein	7	40.0433	30.4383	31.2423	14.6126	2.740327	2.083017	2.138038	-	-

26863	TBIG02686	XLOC_0268	SENP3	GL893964.	2	1393	+	CODING	NOVEL	Sentrin-spe	7	30.0942	28.351	30.6696	14.167	2.124246	2.0012	2.164862	-	-
8976	TBIG00897	ENSSSCG0	NMI	15	3600	29467	-	CODING	NEW_AS	N-myc (an	7	25.4716	25.908	26.6202	12.2474	2.079756	2.115388	2.173539	-	-
28672	TBIG02867	XLOC_0286	Kpna4	GL896327.	9685	13278	+	CODING	NOVEL	Importin su	7	10130.7	5223.81	4254.56	1957.14	5.176278	2.669104	2.173866	-	-
849	TBIG00084	ENSSSCG0	PRPF4	1	2.85E+08	2.85E+08	+	CODING	NEW_AS	pre-mRNA	7	53.261	41.1012	37.7856	17.3289	3.073536	2.37183	2.180496	-	-
26730	TBIG02673	ENSSSCG0	KDELR2	GL893800.	7054	17355	+	CODING	NEW_AS	KDEL (Lys-	7	45.8798	45.7896	42.4378	19.3699	2.368613	2.363956	2.190915	-	-
6156	TBIG00615	ENSSSCG0	COPB2	13	87895226	87930998	-	CODING	NEW_AS	coatomer p	7	74.2013	65.1686	67.5623	30.7361	2.414142	2.120262	2.198142	-	-
25095	TBIG02509	ENSSSCG0	SAMD9	9	79679765	79714213	-	CODING	NEW_AS	sterile alph	7	58.1417	61.0172	49.9107	22.6889	2.562561	2.689297	2.199785	-	-
23067	TBIG02306	ENSSSCG0	TBC1D1	8	30778696	30793855	+	CODING	NEW_AS	Uncharacte	7	45.979	48.4195	43.8218	19.8785	2.313001	2.435772	2.204482	-	-
9682	TBIG00968	ENSSSCG0	IL7R	16	22278436	22314407	+	CODING	NEW_AS	interleukin	7	103.834	64.531	65.7059	29.8034	3.483965	2.165223	2.204644	-	-
3957	TBIG00395	ENSSSCG0	NBR1	12	20093103	20126422	+	CODING	NEW_AS	neighbor o	7	36.1676	27.5106	29.4027	13.3027	2.718816	2.068046	2.210281	-	-
26515	TBIG02651	XLOC_0265	HSPA6	GL893523.	1	293	.	CODING	NOVEL	Heat shock	7	103.493	93.2129	82.0541	36.9707	2.799325	2.521264	2.219436	-	-
21900	TBIG02190	XLOC_0219	FAM65B	7	20752463	20990945	-	CODING	NEW_AS	Protein FA	7	142.59	124.416	119.218	53.5648	2.662009	2.322719	2.225678	-	-
19813	TBIG01981	ENSSSCG0	IST1	6	14464800	14489691	-	CODING	NEW_AS	Uncharacte	7	51.0404	38.8494	37.9261	16.9292	3.014933	2.294816	2.240277	-	-
24530	TBIG02453	ENSSSCG0	PDIA4	9	1.2E+08	1.2E+08	+	CODING	NEW_AS	protein dis	7	88.6251	115.53	83.4976	37.0335	2.393106	3.119608	2.254651	-	-
28296	TBIG02829	XLOC_0282	IGHG4	GL896171.	5	540	+	CODING	NOVEL	Ig gamma-	7	247.21	661.85	276.828	121.593	2.033094	5.443159	2.276677	-	-
7854	TBIG00785	ENSSSCG0	ANKRD13A	14	43479365	43506038	-	CODING	NEW_AS	Ankyrin rep	7	53.4606	38.9802	41.5515	18.2352	2.931725	2.137635	2.278642	-	-
21865	TBIG02186	ENSSSCG0	F13A1	7	3721329	4102833	-	CODING	NEW_AS	Uncharacte	7	349.897	277.55	213.936	93.4317	3.74495	2.970619	2.289758	-	-
19841	TBIG01984	ENSSSCG0	GOT2	6	18501596	18507494	-	CODING	NEW_AS	glutamic-o	7	34.05	29.3039	32.9471	14.3514	2.372591	2.041884	2.295741	-	-
5846	TBIG00584	ENSSSCG0	CMTM6	13	20261232	20287139	-	CODING	NEW_AS	CKLF-like M	7	48.8546	39.7929	38.4579	16.647	2.934739	2.390395	2.3102	-	-
26357	TBIG02635	ENSSSCG0	HA1B_BOV	GL893304.	28077	43963	+	CODING	NEW_AS	BOLA class	7	76.7646	55.8364	57.9475	25.0711	3.061876	2.227122	2.311327	-	-
8519	TBIG00851	ENSSSCG0	DARS	15	18269639	18344270	+	CODING	NEW_AS	aspartyl-tR	7	54.9954	43.8047	44.5019	19.1791	2.867465	2.283981	2.320333	-	-
28137	TBIG02813	ENSSSCG0	CTDSP2	GL895994.	29300	45360	+	CODING	NEW_AS	CTD (carbo	7	96.8499	75.802	83.4617	35.876	2.699574	2.112889	2.326394	-	-
5269	TBIG00526	ENSSSCG0	Nbeal2	13	33122342	33133683	+	CODING	NEW_AS	Uncharacte	7	83.186	65.6626	53.316	22.8995	3.632656	2.867425	2.32826	-	-
2766	TBIG00276	ENSSSCG0	KLF6	10	72992245	73001823	+	CODING	NEW_AS	Kruppel-lik	7	54.4766	36.1548	40.2427	17.2591	3.156399	2.094825	2.33168	-	-
22050	TBIG02205	ENSSSCG0	SLA-DRB*K	7	29113063	29125516	-	CODING	NEW_AS	Sus scrofa	7	145.019	176.295	136.157	58.0712	2.497262	3.035842	2.344656	-	-
10297	TBIG01029	XLOC_0102	gag-pro	17	36964339	36974782	+	CODING	NOVEL	Gag-Pro p	7	53.1501	43.9935	42.6527	18.0607	2.942859	2.435869	2.361631	-	-
29142	TBIG02914	ENSSSCG0	VPS35	GL896549.	6363	23585	-	CODING	NEW_AS	vacuolar p	7	40.8995	35.8611	38.9961	16.4955	2.479434	2.173993	2.364045	-	-
10710	TBIG01071	ENSSSCG0	NDRG3	17	45359657	45427454	-	CODING	NEW_AS	NDRG fam	7	29.3645	26.1356	28.4647	12.0392	2.439074	2.170875	2.364335	-	-
9804	TBIG00980	ENSSSCG0	RNF145	16	70068344	70121376	+	CODING	NEW_AS	ring finger	7	68.0868	48.5401	46.4501	19.524	3.487339	2.486176	2.379128	-	-
17806	TBIG01780	ENSSSCG0	LRRK2	5	74510800	74657314	+	CODING	NEW_AS	Sus scrofa	7	42.6593	32.0993	35.5483	14.8942	2.864155	2.155154	2.386721	-	-
19760	TBIG01976	XLOC_0197	Irf8	6	3330463	3340067	-	CODING	NOVEL	Interferon	7	51.2276	62.8791	33.162	13.8726	3.692718	4.532611	2.390468	-	-
11031	TBIG01103	ENSSSCG0	GIMAP4	18	6609767	6621208	+	CODING	NEW_AS	GTPase, IM	7	681.99	613.324	635.677	264.872	2.574791	2.315549	2.39994	-	-
28410	TBIG02841	ENSSSCG0	SURF4	GL896220.	574	4799	-	CODING	NEW_AS	surfeit 4 [S	7	53.658	62.036	59.3858	24.6938	2.172934	2.51221	2.404887	-	-
7831	TBIG00783	ENSSSCG0	SLC8B1	14	41244220	41251484	-	CODING	NOVEL	solute carr	7	95.9113	107.421	50.6975	20.9724	4.573215	5.122018	2.417344	-	-
10034	TBIG01003	ENSSSCG0	GM2A	16	77974389	77987652	-	CODING	NEW_AS	GM2 gang	7	84.9733	80.5477	62.3129	25.6403	3.314052	3.141449	2.430272	-	-
7292	TBIG00729	ENSSSCG0	MAPK1	14	53590167	53615840	+	CODING	NEW_AS	mitogen-a	7	94.8376	76.3225	76.2807	31.1684	3.042748	2.448714	2.447373	-	-
12463	TBIG01246	ENSSSCG0	REEP5	2	1.21E+08	1.22E+08	+	CODING	NEW_AS	receptor ac	7	107.834	79.594	97.9111	39.7888	2.71016	2.000412	2.46077	-	-
21799	TBIG02179	ENSSSCG0	PAPOLA	7	1.25E+08	1.25E+08	+	CODING	NEW_AS	poly(A) po	7	55.3282	43.4424	51.6498	20.9783	2.637402	2.070826	2.462058	-	-
669	TBIG00066	ENSSSCG0	PTPLAD2	1	2.26E+08	2.26E+08	+	CODING	NEW_AS	protein tyr	7	39.2649	33.3557	27.0746	10.9855	3.574248	3.036339	2.464576	-	-
283	TBIG00028	ENSSSCG0	NARS	1	1.17E+08	1.18E+08	+	CODING	NEW_AS	asparaginy	7	56.5282	41.5603	50.6949	20.5254	2.754061	2.024823	2.469862	-	-
26677	TBIG02667	ENSSSCG0	APMAP	GL893705.	64290	67208	-	CODING	KNOWN	Adipocyte	7	72.161	67.4088	50.6346	20.4636	3.52631	3.294083	2.474374	-	-
7800	TBIG00780	ENSSSCG0	ANAPC7	14	33669495	33718033	-	CODING	NEW_AS	anaphase p	7	26.7281	23.1358	27.8188	11.2345	2.379109	2.059353	2.476194	-	-
16321	TBIG01632	XLOC_0163	ARNT	4	1.08E+08	1.08E+08	+	CODING	NEW_AS	Aryl hydro	7	36.3501	25.9563	31.7854	12.8186	2.835731	2.024894	2.479631	-	-
29769	TBIG02976	ENSSSCG0	TXNDC11	JH118940.	20412	78632	-	CODING	NEW_AS	Uncharacte	7	28.6043	29.7805	27.3208	11.0172	2.596331	2.703092	2.479832	-	-
27165	TBIG02716	ENSSSCG0	GLB1	GL894277.	5160	12749	-	CODING	KNOWN	Uncharacte	7	75.0143	62.4706	75.7414	30.5424	2.456071	2.045373	2.479877	-	-
10611	TBIG01061	ENSSSCG0	ACSS1	17	34949431	34988723	-	CODING	NEW_AS	acyl-CoA s	7	41.1264	43.7668	45.6065	18.3511	2.241086	2.384969	2.485219	-	-
21149	TBIG02114	ENSSSCG0	BLOC1S5	7	5327730	5546412	+	CODING	NOVEL	biogenesis	7	105.903	220.49	125.448	50.0105	2.117615	4.408874	2.508433	-	-
2781	TBIG00278	ENSSSCG0	DCTN5	10	2661	17242	-	CODING	KNOWN	dynactin 5	7	44.4534	43.0362	51.9809	20.7122	2.146242	2.077819	2.509675	-	-
7201	TBIG00720	ENSSSCG0	MLEC	14	43069385	43084765	+	CODING	NEW_AS	malectin [S	7	24.1558	20.7292	25.9667	10.2856	2.348507	2.015361	2.524568	-	-
5193	TBIG00519	ENSSSCG0	UBP1	13	20784893	20832356	+	CODING	NEW_AS	upstream b	7	44.4955	31.7645	38.3249	15.1034	2.946059	2.103136	2.537501	-	-
5228	TBIG00522	ENSSSCG0	CTNNB1	13	27623074	27667302	+	CODING	NEW_AS	Sus scrofa	7	74.8559	55.7844	64.5247	25.4027	2.946769	2.196003	2.540073	-	-
1806	TBIG00180	ENSSSCG0	ROD1	1	2.84E+08	2.84E+08	+	CODING	NEW_AS	Sus scrofa	7	48.5353	37.1763	44.8866	17.5447	2.76638	2.118948	2.558414	-	-
9319	TBIG00931	ENSSSCG0	AGFG1	15	1.43E+08	1.43E+08	-	CODING	NEW_AS	ArfGAP wit	7	26.7001	27.9922	30.6422	11.9064	2.2425	2.351021	2.573591	-	-
28741	TBIG02874	XLOC_0287	Exoc3	GL896361.	60175	62637	+	CODING	NOVEL	Exocyst co	7	27.9099	30.1452	34.8522	13.5323	2.062465	2.227648	2.575482	-	-
9345	TBIG00934	ENSSSCG0	Inpp5d	15	1.48E+08	1.48E+08	-	CODING	NEW_AS	Uncharacte	7	61.8168	54.6138	68.8704	26.6813	2.316859	2.046894	2.581224	-	-
9153	TBIG00915	ENSSSCG0	WIPF1	15	89757199	89837471	-	CODING	NEW_AS	WAS/WAS	7	102.675	77.0715	92.5996	35.8474	2.864224	2.149989	2.583161	-	-
3323	TBIG00332	ENSSSCG0	ELF1	11	26327366	26385565	+	CODING	NEW_AS	E74-like fa	7	91.1587	66.9882	71.7486	27.7247	3.287996	2.416192	2.587895	-	-
30616	TBIG03061	ENSSSCG0	ARHGEF6	X	1.28E+08	1.28E+08	-	CODING	NEW_AS	Rac/Cdc42	7	36.0784	29.9378	31.1084	11.9858	3.010095	2.497772	2.595438	-	-
10713	TBIG01071	ENSSSCG0	SAMHD1	17	45556581	45582808	-	CODING	NEW_AS	Uncharacte	7	87.8642	60.2169	74.5792	28.5531	3.077221	2.108944	2.611948	-	-
19651	TBIG01965	ENSSSCG0	EPS15	6	1.48E+08	1.48E+08	+	CODING	NEW_AS	epidermal	7	31.4888	22.646	26.6061	10.1686	3.09667	2.227052	2.616496	-	-
27156	TBIG02715	ENSSSCG0	NLRC5	GL894265.	7198	10643	+	CODING	NEW_AS	Protein NL	7	214.497	167.438	180.653	68.9464	3.111069	2.428524	2.620195	-	-
15176	TBIG01517	ENSSSCG0	RPIA	3	59834045	59864868	-	CODING	NEW_AS	ribose 5-pl	7	45.2459	42.6705	55.3274	21.0839	2.145993	2.023843	2.624154	-	-
24949	TBIG02494	ENSSSCG0	ARCN1	9	50814157	50842328	-	CODING	NEW_AS	archain 1 [	7	61.1143	47.1671	57.5497	21.8832	2.79275	2.155402	2.629858	-	-
1392	TBIG00139	XLOC_0013	CCPG1	1	1.29E+08	1.29E+08	-	CODING	NOVEL	Cell cycle p	7	115.258	63.7906	71.5037	27.1424	4.246419	2.35022	2.634391	-	-
13480	TBIG01348	ENSSSCG0	Pja2	2	1.19E+08	1.19E+08	-	CODING	NEW_AS	E3 ubiquiti	7	28.3978	20.9376	27.5871	10.4131	2.727123	2.010698	2.649269	-	-
14226	TBIG01422	ENSSSCG0	PRKCB	3	22971181	23009237	+	CODING	NEW_AS	protein kin	7	51.4333	45.1624	54.7554	20.641	2.491803	2.187995	2.652749	-	-
9707	TBIG00970	ENSSSCG0	NNT	16	29705772	29786703	+	CODING	NEW_AS	NAD(P) tra	7	32.3799	25.6384	29.848	11.2491	2.878444	2.279151	2.653368	-	-
26608	TBIG02660	ENSSSCG0	MARK3	GL893654.	12	32731	-	CODING	NEW_AS	Uncharacte	7	81.9549	67.6409	85.469	32.1114	2.552206	2.106445	2.66164	-	-
11852	TBIG01185	ENSSSCG0	LPXN	2	12191484	12243400	+	CODING	KNOWN	leupaxin [S	7	53.9739	42.0414	51.5851	19.3675	2.786828	2.170719	2.663488	-	-
9664	TBIG00966	ENSSSCG0	FAM105A	16	4309762	4346624	+	CODING	NEW_AS	family with	7	54.4012	31.4626	35.0564	13.1441	4.13883	2.393667	2.667083	-	-
5735	TBIG00573	ENSSSCG0	GBE1	13	1.84E+08	1.84E+08	+	CODING	NEW_AS	Uncharacte	7	64.7699	52.4968	50.4549	18.8961	3.427686	2.778182	2.670122	-	-
30530	TBIG03053	ENSSSCG0	MORF4L2	X	98342576	98355097	-	CODING	NEW_AS	mortality fa	7	30.4664	25.9728	30.097	11.2629	2.705023	2.306049	2.672225	-	-
2661	TBIG00266	ENSSSCG0	AQP3	10	37307556	37313382	+	CODING	KNOWN	Sus scrofa	7	63.967	93.91	53.8616	20.0913	3.183816	4.674162	2.680842	-	-
12476	TBIG01247	XLOC_0124	HSD17B4	2	1.29E+08	1.29E+08	+	CODING	NEW_AS	Peroxisom	7	68.692	51.5696	68.1237	25.3193	2.713029	2.03677	2.690584	-	-
19520	TBIG01952	ENSSSCG0	RAB31	6	91609342	91647903	+	CODING	NEW_AS	RAB31, me	7	40.3675	32.5414	34.6946	12.8833	3.13332	2.525859	2.69299	-	-
11442	TBIG01144	ENSSSCG0	SNX10	18	50837877	50917746	-	CODING	NEW_AS	sorting nex	7	76.1467	65.7446	57.8349	21.4727	3.54621	3.061776	2.693415	-	-
18151	TBIG01815	ENSSSCG0	CS	5	23083204	23085626	-	CODING	KNOWN	Citrate syn	7	58.9414	54.6274	71.7837	26.5638	2.218862	2.05646	2.702313	-	-
25177	TBIG02517	ENSSSCG0	PIK3CG	9	1.18E+08	1.18E+08	-	CODING	NEW_AS	phosphatid	7	56.1736	46.5746	52.303	19.2167	2.923166	2.423652	2.721747	-	-
23159	TBIG02315	ENSSSCG0	POLR2B	8	59008839	59067819	+	CODING	NEW_AS	polymerase	7	41.2028	29.7103	34.1917	12.5489	3.283379	2.367562	2.724677	-	-
13529	TBIG01352	ENSSSCG0	C5orf15	2	1.42E+08	1.42E+08	-	CODING	KNOWN	chromosom	7	41.5471	29.3219	36.2363	13.225	3.141558	2.217157	2.739985	-	-
14483	TBIG01448	ENSSSCG0	TGOLN2	3	62299083	62309371	+	CODING	NEW_AS	trans-golg	7	45.4562	32.7505	37.458	13.6634	3.326859	2.396951	2.741485	-	-
28970	TBIG02897	ENSSSCG0	CDKN1B	GL896456.	60363	64021	+	CODING	KNOWN	cyclin-dep	7	59.8863	57.3193	70.3979	25.6331	2.336288	2.236144	2.746367	-	-
10535	TBIG01053	XLOC_0105	HGSNAT	17	9532025	9545186	-	CODING	NOVEL	Heparan-a	7	31.1589	24.583	27.8704	10.1372	3.073719	2.425029	2.749319	-	-
8684	TBIG00868	ENSSSCG0	CYBRD1	15	87032975	87079679	+	CODING	NEW_AS	Sus scrofa	7	48.3702	37.609	39.225	14.2619	3.391568	2.637026	2.750335	-	-
1507	TBIG00150	ENSSSCG0	KLF13	1	1.59E+08	1.59E+08	-	CODING	NEW_AS	Sus scrofa	7	69.4876	51.6382	61.6525	22.1307	3.139874	2.333329	2.785836	-	-
14582	TBIG01458	XLOC_0145	RTN4	3	91012599	91202777	+	CODING	NEW_AS	Reticulon-4	7	54.9626	40.1293	45.999	16.5114	3.328767	2.4304	2.785893	-	-
12002	TBIG01200	ENSSSCG0	Copb1	2	47986873	48009866	+	CODING	KNOWN	Sus scrofa	7	45.0853	38.7239	46.4329	16.6276	2.711474	2.328893	2.79252	-	-
11238	TBIG01123	ENSSSCG0	UBE3C	18	1571837	1621759	-	CODING	NEW_AS	Ubiquitin-p	7	51.0008	34.3378	38.9571	13.7557	3.707612	2.49626	2.83207	-	-
5531	TBIG00553	ENSSSCG0	MFSD1	13	1.07E+08	1.07E+08	+	CODING	NEW_AS	major facil	7	49.9696	40.9539	49.7913	17.5688	2.844224	2.331058	2.834075	-	-
27861	TBIG02786	ENSSSCG0	YIPF5	GL895451.	8356	11273	-	CODING	KNOWN	Yip1 doma	7	34.3046	31.9177	42.5058	14.8753	2.306145	2.145684	2.857475	-	-
5843	TBIG00584	ENSSSCG0	TGFBR2	13	18707605	18726303	-	CODING	NEW_AS	transformi	7	69.1291	56.0461	65.065	22.6102	3.05743	2.478797	2.877684	-	-
13378	TBIG01337	ENSSSCG0	HNRNPAB	2	81799737	81810816	-	CODING	NEW_AS	heterogen	7	161.164	129.772	176.028	60.8984	2.646441	2.130959	2.890519	-	-
8719	TBIG00871	ENSSSCG0	ITGA4	15	96518760	96605885	+	CODING	NEW_AS	Integrin, al	7	93.099	57.7489	70.28	24.299	3.831392	2.376596	2.8923	-	-
17412	TBIG01741	ENSSSCG0	DDX17	5	6766421	6785862	+	CODING	NEW_AS	DEAD (Asp	7	298.66	195.584	246.971	85.1272	3.508397	2.29755	2.9012	-	-
15174	TBIG01517	XLOC_015	KV206_HU	3	59749591	59963248	-	CODING	NEW_AS	Ig kappa c	7	757.91	1564.35	501.378	172.364	4.397148	9.075851	2.908832	-	-
27154	TBIG02715	XLOC_0271	NLRC5	GL894265.	1495	3839	+	CODING	NOVEL	Protein NL	7	62.2319	48.9499	60.518	20.6939	3.007258	2.365427	2.924437	-	-
16025	TBIG01602	ENSSSCG0	YWHAZ	4	38913852	38942797	+	CODING	NEW_AS	Uncharacte	7	341.654	255.785	344.492	117.321	2.91213	2.180215	2.93632	-	YWHAZ
5516	TBIG00551	ENSSSCG0	RAP2B	13	1.01E+08	1.01E+08	+	CODING	NEW_AS	RAP2B, me	7	51.8803	41.0348	58.8841	19.932	2.602865	2.05874	2.954249	-	-
8642	TBIG00864	ENSSSCG0	CYTIP	15	71188248	71214563	+	CODING	KNOWN	cytohesin 1	7	98.5347	69.3567	94.7491	31.945	3.084511	2.171129	2.966007	-	-
28412	TBIG02841	ENSSSCG0	SF3B3	GL896221.	4875	11725	+	CODING	NEW_AS	Splicing fa	7	81.3587	45.01	61.515	20.6755	3.935029	2.176973	2.975261	-	-
10331	TBIG01033	ENSSSCG0	TPX2	17	40431400	40452999	+	CODING	NEW_AS	Uncharacte	7	38.0264	26.1071	35.6647	11.9855	3.1727	2.178224	2.975654	-	-
14553	TBIG01455	ENSSSCG0	RAB1A	3	80675084	80714133	+	CODING	NEW_AS	RAB1A, me	7	61.3897	45.0444	60.8817	20.1327	3.049253	2.237375	3.024021	-	-
1364	TBIG00136	ENSSSCG0	RAB8B	1	1.2E+08	1.2E+08	-	CODING	NEW_AS	RAB8B, me	7	58.3217	36.0193	52.2682	17.2431	3.382321	2.088911	3.031253	-	-
30565	TBIG03056	ENSSSCG0	LAMP2	X	1.13E+08	1.13E+08	-	CODING	NEW_AS	lysosomal-	7	82.3309	63.8295	67.8469	22.2573	3.699052	2.867801	3.048299	-	-
14566	TBIG01456	ENSSSCG0	XPO1	3	84163385	84182474	+	CODING	KNOWN	exportin 1	7	31.0369	26.5938	37.4301	12.2218	2.53947	2.175932	3.062569	-	-

5736	TBIG00573	XLOC_005	GBE1	13	1.84E+08	1.84E+08	+		CODING	NOVEL	1,4-alpha-	7	74.1595	70.6589	78.2403	25.4132	2.918149	2.780402	3.078727	-	-
13370	TBIG01337	ENSSSCG0	CANX	2	80445609	80482509	-		CODING	KNOWN	calnexin [S	7	54.6557	40.8975	51.797	16.8076	3.251844	2.433274	3.081761	-	-
16477	TBIG01647	ENSSSCG0	GBP1	4	1.39E+08	1.4E+08	+		CODING	NEW_AS	Sus scrofa	7	191.634	131.65	180.467	57.8376	3.313312	2.276201	3.120237	-	-
20551	TBIG02055	ENSSSCG0	JAK1	6	1.36E+08	1.36E+08	-		CODING	NEW_AS	Janus kina	7	200.684	127.755	143.562	45.9455	4.367871	2.780577	3.124615	-	-
17433	TBIG01743	ENSSSCG0	IL2RB	5	8223890	8249820	+		CODING	NEW_AS	Uncharacte	7	83.5944	53.3671	79.5051	25.4298	3.287261	2.098605	3.126454	-	-
14174	TBIG01417	XLOC_014	GBAS	3	17205926	17219756	+		CODING	NOVEL	Protein Ni	7	57.5279	47.9816	62.7497	19.8058	2.904599	2.422603	3.168249	-	-
27593	TBIG02759	ENSSSCG0	ELP3	GL894948.	1037	6292	+		CODING	KNOWN	Uncharacte	7	36.4931	26.4841	37.4437	11.7946	3.094052	2.245443	3.174648	-	-
22505	TBIG02250	ENSSSCG0	TMED10	7	1.04E+08	1.04E+08	-		CODING	NEW_AS	transmemb	7	56.1532	42.405	55.4414	17.3964	3.227863	2.437573	3.186947	-	-
27159	TBIG02715	XLOC_027	Jak1	GL894267.	3	422	.		CODING	NOVEL	Tyrosine-p	7	148.38	95.8767	127.726	40.0643	3.703547	2.393071	3.188025	-	-
6206	TBIG00620	ENSSSCG0	CCNL1	13	1.05E+08	1.05E+08	-		CODING	NEW_AS	cyclin L1 [	7	42.4061	32.4785	43.9096	13.742	3.085875	2.363448	3.195285	-	-
15270	TBIG01527	ENSSSCG0	ACTR2	3	80487337	80525402	-		CODING	KNOWN	Sus scrofa	7	192.817	125.065	198.3	61.4546	3.137552	2.03508	3.226772	-	-
16714	TBIG01671	ENSSSCG0	RCSD1	4	90996577	91079847	-		CODING	NEW_AS	RCSD dom	7	69.7956	53.0808	79.5734	24.592	2.838142	2.158458	3.235743	-	-
14375	TBIG01437	ENSSSCG0	FAM120A	3	44107429	44330948	+		CODING	NEW_AS	family with	7	36.7947	26.4047	41.9108	12.9051	2.851175	2.046067	3.247615	-	-
1532	TBIG00153	ENSSSCG0	VPS4B	1	1.76E+08	1.76E+08	-		CODING	NEW_AS	vacuolar p	7	38.9495	28.78	45.7719	14.0411	2.773964	2.049697	3.259851	-	-
23467	TBIG02346	XLOC_023	TLR6	8	31625693	31660825	-		CODING	NEW_AS	Toll-like re	7	30.051	24.2306	35.6839	10.9276	2.750009	2.217376	3.265484	-	-
6363	TBIG00636	ENSSSCG0	ATP6V1A	13	1.56E+08	1.56E+08	-		CODING	NEW_AS	ATPase, H-	7	30.805	24.7878	34.899	10.6734	2.886147	2.32239	3.269717	-	-
23594	TBIG02359	ENSSSCG0	G3BP2	8	75410919	75499534	-		CODING	NEW_AS	GTPase ac	7	33.475	21.596	35.01	10.5393	3.176207	2.049092	3.321852	-	-
28212	TBIG02821	ENSSSCG0	YIPF5	GL896100.	827	9421	-		CODING	KNOWN	protein YI	7	41.5617	32.426	51.9762	15.5571	2.671558	2.084322	3.340995	-	-
30078	TBIG03007	ENSSSCG0	OGT	X	64962766	65000187	+		CODING	NEW_AS	Sus scrofa	7	111.033	75.2087	117.309	35.0493	3.167909	2.145797	3.346971	-	-
15358	TBIG01535	ENSSSCG0	PRKD3	3	1.09E+08	1.1E+08	-		CODING	NEW_AS	protein kin	7	44.5871	28.3631	39.2181	11.67	3.82066	2.430428	3.360591	-	-
1479	TBIG00147	ENSSSCG0	THBS1	1	1.47E+08	1.47E+08	-		CODING	NEW_AS	Sus scrofa	7	79.0614	46.4264	40.2191	11.9141	6.635952	3.896761	3.375756	-	-
9990	TBIG00999	XLOC_009	DOCK2	16	58744789	58751935	-		CODING	NOVEL	Dedicator	7	78.0226	63.5007	94.6406	27.8784	2.798676	2.277774	3.394764	-	-
27870	TBIG02787	ENSSSCG0	HIATL1	GL895478.	11241	49084	-		CODING	NEW_AS	Sus scrofa	7	47.1809	37.9099	34.7135	10.0602	4.689857	3.768305	3.450578	-	-
5459	TBIG00545	ENSSSCG0	SRPRB	13	82474461	82496616	+		CODING	KNOWN	signal reco	7	51.5088	40.1247	54.1702	15.4301	3.338203	2.600417	3.510684	-	-
2860	TBIG00286	ENSSSCG0	PTPRC	10	26246556	26287845	-		CODING	NEW_AS	Uncharacte	7	393.984	271.914	396.897	112.639	3.497758	2.414031	3.52362	-	-
3345	TBIG00334	ENSSSCG0	NDFIP2	11	56246092	56312418	+		CODING	NEW_AS	Nedd4 fan	7	54.5985	29.9039	49.7824	14.115	3.868119	2.11859	3.526915	-	-
9201	TBIG00920	ENSSSCG0	STK17B	15	1.11E+08	1.11E+08	-		CODING	NEW_AS	serine/thre	7	122.075	71.9184	115.152	32.107	3.80213	2.23996	3.586508	-	-
27266	TBIG02726	XLOC_027	Dync1h1	GL894475.	19	942	+		CODING	NOVEL	Cytoplasm	7	27.1342	20.8042	36.3496	10.0862	2.69023	2.06264	3.603894	-	-
29714	TBIG02971	XLOC_029	TM9SF2	JH118897.	8484	27691	+		CODING	NOVEL	Transmem	7	55.1815	44.7188	68.8485	19.0685	2.893856	2.345166	3.610588	-	-
873	TBIG00087	ENSSSCG0	RAB14	1	2.93E+08	2.94E+08	+		CODING	NEW_AS	RAB14, me	7	37.8024	30.173	44.1384	12.1287	3.116773	2.487736	3.63917	-	-
10459	TBIG01045	ENSSSCG0	CSE1L	17	57095159	57109228	+		CODING	KNOWN	CSE1 chro	7	79.6241	39.0217	59.4584	15.9775	4.983514	2.442291	3.721383	-	-
16083	TBIG01608	ENSSSCG0	TRAM1	4	70399155	70431004	+		CODING	NEW_AS	translocati	7	80.5535	63.1363	90.0989	24.0047	3.355739	2.630164	3.753386	-	-
5535	TBIG00553	ENSSSCG0	NLRP1	13	1.08E+08	1.08E+08	+		CODING	KNOWN	Uncharacte	7	46.6606	26.0405	48.6281	12.8257	3.638055	2.030338	3.791458	-	-
27758	TBIG02775	XLOC_027	Tbk1	GL895245.	669	795	.		CODING	NOVEL	Serine/thre	7	23969	31712.9	32182.5	8448.48	2.837078	3.753681	3.809265	-	-
15977	TBIG01597	ENSSSCG0	KIAA0196	4	15149903	15246609	+		CODING	NEW_AS	WASH con	7	37.1102	31.7119	44.7795	11.649	3.185698	2.722285	3.844064	-	-
30599	TBIG03059	ENSSSCG0	MST4	X	1.24E+08	1.24E+08	-		CODING	NEW_AS	Serine/thre	7	39.8961	20.2312	38.4758	10.0046	3.987776	2.02219	3.845811	-	-
10742	TBIG01074	ENSSSCG0	STK4	17	52872154	52878619	-		CODING	KNOWN	serine/thre	7	73.6392	51.4528	77.8127	19.9957	3.682752	2.573193	3.891472	-	-
23575	TBIG02357	ENSSSCG0	IGJ	8	71347284	71358511	-		CODING	KNOWN	immunogl	7	442.434	585.16	476.436	122.085	3.623983	4.793054	3.902494	-	-
26699	TBIG02669	XLOC_026	INTS12	GL893739.	1798	12539	-		CODING	NOVEL	Integrator	7	53.9285	59.7287	72.6546	18.2814	2.949911	3.267184	3.974236	-	-
20352	TBIG02035	ENSSSCG0	ARID1A	6	77607451	77612795	-		CODING	KNOWN	AT rich int	7	39.2487	24.6264	40.7517	10.186	3.8532	2.417671	4.000756	-	-
29283	TBIG02928	ENSSSCG0	RAB5A	JH118496.	1977	24283	-		CODING	KNOWN	RAB5A, me	7	34.2504	28.9413	54.9243	13.7102	2.498169	2.110932	4.00609	-	-
528	TBIG00052	XLOC_000	Zwilch	1	1.82E+08	1.82E+08	+		CODING	NOVEL	Protein zw	7	22.4381	21.2835	42.6459	10.5359	2.12968	2.020093	4.047675	-	-
16950	TBIG01695	ENSSSCG0	ST7L	4	1.18E+08	1.18E+08	-		CODING	NOVEL	suppressio	7	59.0845	28.5359	46.1817	11.266	5.244497	2.532922	4.09921	-	-
30491	TBIG03049	ENSSSCG0	MAGT1	X	70812442	70877254	-		CODING	NEW_AS	magnesiu	7	43.3067	32.2748	51.1111	12.4321	3.483458	2.596086	4.11122	-	-
29419	TBIG02941	ENSSSCG0	ZCCHC4	JH118638.	93888	288709	+		CODING	NEW_AS	Uncharacte	7	85.9682	12.3849	157.937	38.3551	2.241376	-3.09692	4.117757	-	-
29981	TBIG02998	ENSSSCG0	DDX3X	X	41161892	41177782	+		CODING	KNOWN	Uncharacte	7	51.7024	29.5464	60.9819	14.6258	3.535013	2.020156	4.169474	-	-
136	TBIG00013	ENSSSCG0	SERINC1	1	44519709	44540322	+		CODING	NEW_AS	Uncharacte	7	161.371	77.035	130.549	30.9315	5.217044	2.490503	4.220584	-	-
15750	TBIG01575	XLOC_015	EXOC6B	3	73146787	73147018	.		CODING	NOVEL	Exocyst co	7	259.786	156.403	312.54	73.951	3.512948	2.114954	4.226312	-	-
27152	TBIG02715	XLOC_027	Nomo1	GL894264.	4444	10080	-		CODING	NOVEL	Nodal mo	7	62.8315	51.9085	92.2525	21.3114	2.948258	2.435715	4.328786	-	-
17904	TBIG01790	XLOC_017	PPP1R12A	5	1.06E+08	1.07E+08	+		CODING	NOVEL	Protein ph	7	70.0987	44.0703	71.446	15.8748	4.415722	2.776117	4.500592	-	-
620	TBIG00062	ENSSSCG0	HIF1A	1	2.12E+08	2.12E+08	+		CODING	NEW_AS	Sus scrofa	7	51.869	33.759	57.0442	12.5835	4.121985	2.682799	4.533254	-	-
1266	TBIG00126	ENSSSCG0	CD164	1	84719535	84736132	-		CODING	NEW_AS	Sialomucin	7	85.3405	63.603	120.15	25.3557	3.365732	2.50843	4.738579	-	-
13463	TBIG01346	ENSSSCG0	ERAP2	2	1.07E+08	1.07E+08	-		CODING	NEW_AS	Uncharacte	7	57.3708	32.2542	58.4791	12.2971	4.665393	2.622911	4.75552	-	-
10150	TBIG01015	XLOC_010	Dock2	16	58655196	58655489	.		CODING	NOVEL	Dedicator	7	77.1889	59.9841	103.385	21.7224	3.553424	2.761394	4.759373	-	-
25929	TBIG02592	XLOC_025	MCM6	GL892768.	76	196	.		CODING	NOVEL	DNA replic	7	200063	361204	135128	28158.2	7.104964	12.82767	4.798886	-	-
15175	TBIG01517	ENSSSCG0	IGKV1-5	3	59753693	59754231	-		CODING	KNOWN	Uncharacte	7	292.246	311.217	338.211	69.6683	4.19482	4.467125	4.85459	-	-
24877	TBIG02487	XLOC_024	TMEM123	9	37031495	37080859	-		CODING	NOVEL	Porimin [S	7	85.9356	43.0955	105.712	21.3824	4.018988	2.015466	4.943879	-	-
18202	TBIG01820	ENSSSCG0	GNS	5	32134175	32173480	-		CODING	KNOWN	glucosami	7	43.081	37.3886	55.5237	11.1391	3.867548	3.356519	4.984577	-	-
11082	TBIG01108	XLOC_011	Mtpn	18	14099503	14151591	+		CODING	NOVEL	Myotrophi	7	45.4978	29.1578	73.1138	14.3838	3.163128	2.027128	5.083066	-	-
2977	TBIG00297	ENSSSCG0	ITGB1	10	61450695	61521893	-		CODING	NEW_AS	integrin, b	7	96.9823	52.3312	89.0997	16.9243	5.730358	3.092075	5.264602	-	-
9208	TBIG00920	ENSSSCG0	SF3B1	15	1.12E+08	1.13E+08	-		CODING	NEW_AS	splicing fa	7	108.572	59.6028	131.111	24.4846	4.434297	2.434297	5.354835	-	-
7577	TBIG00757	ENSSSCG0	ADD3	14	1.31E+08	1.31E+08	+		CODING	NEW_AS	adducin 3	7	76.7192	41.1507	85.1385	15.7562	4.869144	2.611715	5.403492	-	-
10070	TBIG01007	XLOC_010	Golph3	16	18722534	18722999	.		CODING	NOVEL	Golgi pho	7	76.0818	53.2894	106.874	19.6476	3.87232	2.71226	5.439545	-	-
26701	TBIG02670	XLOC_026	LBR	GL893745.	6	316	.		CODING	NOVEL	Lamin-B re	7	283.05	126.411	280.792	46.0386	6.148102	2.745761	6.099056	-	-
23321	TBIG02332	ENSSSCG0	NFKB1	8	1.28E+08	1.28E+08	+		CODING	NEW_AS	Sus scrofa	7	1396.76	966.043	2246.45	313.896	4.449754	3.077589	7.15667	-	-
14840	TBIG01484	XLOC_014	Cux1	3	9011740	9052253	-		CODING	NOVEL	Homeobo	7	950.021	522.404	136.317	16.0006	59.37409	32.64903	8.519493	-	-
12988	TBIG01298	ENSSSCG0	Cstf3	2	30415041	30415587	-		CODING	KNOWN	Uncharacte	7	44.2239	37.5594	103.651	11.8821	3.721893	3.161007	8.72329	-	-
10714	TBIG01071	ENSSSCG0	SAMHD1	17	45605094	45611771	-		CODING	NEW_AS	Uncharacte	7	96.5363	61.8119	160.782	18.262	5.286184	3.384728	8.804184	-	-
27042	TBIG02704	XLOC_027	CD163	GL894145.	1	259	.		CODING	NOVEL	Scavenger	7	279.277	421.821	1311.79	135.942	2.054383	3.102948	9.64963	-	-
21195	TBIG02119	ENSSSCG0	HIST1H1D	7	22072600	22073540	+		CODING	NEW_AS	Sus scrofa	7	105.837	86.3732	214.229	22.1402	4.780309	3.901193	9.676019	-	-
28965	TBIG02896	XLOC_028	NOTCH1	GL896453.	1876	2559	+		CODING	NOVEL	Neurogeni	7	34.5991	49.7613	169.766	17.2188	2.009379	2.88994	9.85934	-	-
719	TBIG00071	XLOC_000	MAMDC2	1	2.5E+08	2.5E+08	+		CODING	NOVEL	MAM dom	7	137.121	178.83	445.262	35.0109	3.916523	5.107838	12.71781	-	-
28583	TBIG02858	XLOC_028	VPS35	GL896287.	1247	12415	-		CODING	NOVEL	Vacuolar p	7	327.301	138.502	816.888	64.1871	5.099171	2.157786	12.72667	-	-
26047	TBIG02604	XLOC_026	mau2	GL892930.	21928	24963	-		CODING	NOVEL	MAU2 chr	7	419.07	257.043	1189.71	92.9927	4.506483	2.76412	12.79358	-	-
17905	TBIG01790	XLOC_017	Ppp1r12a	5	1.06E+08	1.07E+08	+		CODING	NOVEL	Protein ph	7	83.1259	33.393	142.039	10.1766	8.168337	3.281351	13.95741	-	-
2403	TBIG00240	XLOC_002	MAMDC2	1	2.5E+08	2.5E+08	.		CODING	NOVEL	MAM dom	7	59.7257	86.3194	226.35	14.676	4.069617	5.881671	15.42314	-	-
6015	TBIG00601	XLOC_006	Slmap	13	43454339	43455163	-		CODING	NOVEL	Sarcolemn	7	70.5552	50.6421	272.511	16.0732	4.389617	3.150717	16.95437	-	-
2736	TBIG00273	XLOC_002	Camk1d	10	65034437	65157840	+		CODING	NOVEL	Calcium/ca	7	29.5895	20.8105	177.457	10.3941	2.846759	2.002145	17.07286	-	-
7276	TBIG00727	XLOC_007	LAC_PIG	14	52541414	52542893	+		CODING	NOVEL	Ig lambda	7	136911	214473	97621.9	961.79	142.3502	222.9936	101.5002	-	-
